# Strategies for Controlling the Spatial Orientation of Single Molecules Tethered on DNA Origami Templates Physisorbed on Glass Substrates: Intercalation and Stretching

**DOI:** 10.3390/ijms23147690

**Published:** 2022-07-12

**Authors:** Keitel Cervantes-Salguero, Austin Biaggne, John M. Youngsman, Brett M. Ward, Young C. Kim, Lan Li, John A. Hall, William B. Knowlton, Elton Graugnard, Wan Kuang

**Affiliations:** 1 Micron School of Materials Science and Engineering, Boise State University, Boise, ID 83725, USA; austinbiaggne@u.boisestate.edu (A.B.); johnyoungsman@boisestate.edu (J.M.Y.); brettward@u.boisestate.edu (B.M.W.); lanli@boisestate.edu (L.L.); bknowlton@boisestate.edu (W.B.K.); eltongraugnard@boisestate.edu (E.G.); 2 Materials Science and Technology Division, U.S. Naval Research Laboratory, Code 6300, Washington, DC 20375, USA; youngchan.kim@nrl.navy.mil; 3 Center for Advanced Energy Studies, Idaho Falls, ID 83401, USA; 4 Division of Research and Economic Development, Boise State University, Boise, ID 83725, USA; johnhall440@boisestate.edu; 5 Department of Electrical and Computer Engineering, Boise State University, Boise, ID 83725, USA

**Keywords:** DNA origami, nanoarchitectonics, single molecules, orientation control, dipolar imaging, super-resolution microscopy, DNA-PAINT, intercalation, mechanical stretching, cyanine, Cy5

## Abstract

Nanoarchitectural control of matter is crucial for next-generation technologies. DNA origami templates are harnessed to accurately position single molecules; however, direct single molecule evidence is lacking regarding how well DNA origami can control the orientation of such molecules in three-dimensional space, as well as the factors affecting control. Here, we present two strategies for controlling the polar (***θ***) and in-plane azimuthal (***ϕ***) angular orientations of cyanine Cy5 single molecules tethered on rationally-designed DNA origami templates that are physically adsorbed (physisorbed) on glass substrates. By using dipolar imaging to evaluate Cy5′s orientation and super-resolution microscopy, the absolute spatial orientation of Cy5 is calculated relative to the DNA template. The sequence-dependent partial intercalation of Cy5 is discovered and supported theoretically using density functional theory and molecular dynamics simulations, and it is harnessed as our first strategy to achieve ***θ*** control for a full revolution with dispersion as small as ±4.5°. In our second strategy, ***ϕ*** control is achieved by mechanically stretching the Cy5 from its two tethers, being the dispersion ±10.3° for full stretching. These results can in principle be applied to any single molecule, expanding in this way the capabilities of DNA as a functional templating material for single-molecule orientation control. The experimental and modeling insights provided herein will help engineer similar self-assembling molecular systems based on polymers, such as RNA and proteins.

## 1. Introduction

Functional materials and techniques for precise positioning [1,2,3,4,5] and orientational control [3,4,5,6,7,8,9,10] of single molecules are highly desirable for engineering a new generation of systems and devices that exploit the intrinsic properties of molecules with nanoarchitectural control [11,12]. Although nature uses proteins as templates to orchestrate the directional arrangement of molecules [13], scientists have harnessed DNA as a programmable polymer [1], with specific self-assembly via Watson-Crick hydrogen base-pairing rules, to fabricate templates of nanometric spacing. Single molecules and single nanoparticles have been spatially arranged using DNA for applications including tissue engineering [14], cellular delivery [15], single molecule biosensing [16,17], nanomemories [18], nanophotonics [19,20,21], and excitonics [22,23,24]. 

DNA templates in two- and three-dimensions are fabricated using the DNA origami method [25,26,27], in which a long circular DNA, called a scaffold, and hundreds of short DNA strands, called staples, self-assemble into nanostructures with a high yield close to 100% [28]. Single molecules can be placed on the DNA origami template by tethering them via covalent bonds to selected staples. Fluorescence single molecules, dyes hereinafter, are extensively used mainly for exploiting their optical properties in arrangements of individual dyes on DNA origami constructs [29]. In one application, the separation of two dyes with a distance resolution of ~0.04 nm was achieved using a hinged construct [30]. Although dyes can be precisely positioned on the DNA origami template under cryogenic temperature conditions [31], in the last few years the impact of the dye’s surrounding environment [32]—i.e., DNA breathing [33,34], DNA molecular structure [35], dye-DNA interactions [36,37,38,39], and linker type [40]—has been recognized as a crucial factor governing dye orientation relative to the DNA molecule and, consequently, affecting the performance of the particular applications [32,35,36,37,38,39,40]. All these efforts indicate that much remains to be learned about the DNA molecule [41], particularly when using it as a functional material for molecular organization, including orientational control. Nevertheless, towards achieving precise DNA nanoarchitectures [42,43,44], a physicochemical understanding of the environment combined with quantification of its impacts on the orientation of single molecules is required.

Historically, ensemble measurements have provided insights into the orientation of single dyes covalently tethered to DNA constructs free in solution [3,36,45,46]. A general strategy is relying on the dye’s transition dipole moment (TDM), which is associated with a harmonic oscillator typically positioned along the long axis of the dye when the dye is optically excited. A typical example of this strategy is fitting the bulk Förster resonance energy transfer (FRET) between the TDMs of two dyes templated on a DNA duplex to obtain the relative orientation of the dyes [3,45,46]. As a result, singly tethered cyanine Cy3 and Cy5 dyes have been shown to stack to the DNA terminal bases [45,46], which was supported by NMR studies [47]. Recently, using fluorescence lifetime experiments in solution, Mathur et al. found that a DNA origami constrains a Cy3 dye more than the simple DNA duplex counterpart [36]. Simulations using molecular dynamics (MD) suggest that this constraint is possible due to major groove binding for a single phosphodiester dye attachment [36].

Directly measuring the orientation of the TDM of the dye on a surface at the single molecule level is of particular interest, and it is often achieved through two methods that are based on absorption/emission polarization measurements and anisotropic point spread function (PSF) fitting [48,49]. As an instance of the first method, Hübner et al. recently pioneered a combined emission polarization and super-resolution microscopy strategy to measure Cy5′s in-plane orientation relative to a DNA origami fixed on a protein-passivated surface [37]. In the second method, the distinct emission pattern of a fixed dye located near a dielectric interface can be captured by dipolar imaging, and this pattern can be fitted by anisotropic PSF to obtain the TDM orientation in spherical coordinates. This latter method has been used to calculate the relative orientation between two color dyes that were distanced on a DNA duplex physisorbed on a polylysine-passivated glass [49]. In single molecule experiments, passivation allows fixing target molecules while at the same time limiting non-specific binding. This comes, however, at the expense of increasing surface roughness—whose heterogeneity can lead to height-related artifacts of ~24° inclination angle when using passivating proteins for immobilizing DNA constructs [50]—as well as potential fouling and autofluorescence due to non-specific interactions arising from an additional number of functionalization steps [51]. In this regard, a plasma-treatment approach for firmly immobilizing flat DNA origami constructs on the glass surface has been demonstrated offering direct interfacing with the surface, while keeping a low background noise suitable for super-resolution microscopy [18,52]. All in all, although the orientation of dyes has been characterized by ensemble, single molecule measurements, and molecular simulations [36,37,45,46,53], little direct single molecule evidence has been reported on the absolute precision and control with which DNA—and the DNA origami template by extension—orients dyes in three-dimensional space and the actual molecular mechanisms through which the surrounding environment affects the orientation.

Here, we directly calculate the spatial orientation of fluorescent single molecules and systematically examine the factors affecting such orientations by using a rationally designed DNA origami template. The dye Cy5 (non-sulfonated cyanine 5, diIC2(5)) was selected as a model single molecule dye member of the cyanine family, which has broad applicability in the biophysical [54], bioimaging [55], super-resolution microscopy [56], optoelectronics [57], electrochemical [58], and DNA nanotechnology fields. In our work, a single Cy5 was positioned along a DNA double helix in the template, which was subsequently physisorbed onto a surface as reported previously [52]. Measurements of the dye orientations relative to the origami’s coordinate axes were obtained through the combination of two single-molecule optical techniques: single dipole imaging and DNA points accumulation in nanoscale topography (DNA-PAINT). We first introduce the design of the DNA origami template, the spatial coordinates, and the single molecule measurements. Then, we demonstrate the dependence of stable Cy5 orientations on the surrounding DNA bases, and harness these stable orientations for polar (***θ***) angle control. We propose the partial intercalation of Cy5 as the most likely molecular mechanism for interpreting our experimental data based on the molecular geometry of DNA, performed MD simulations, and first-principles density functional theory (DFT) calculations. We also describe additional experiments designed to assess the interaction of the surface on the dye orientation, which further confirm the partial intercalation mechanism. Finally, we establish the mechanical stretching of Cy5 from its two linkers to achieve in-plane (azimuthal, ***ϕ***) angle control. Experimental results were further investigated using the molecular geometry of DNA, and MD simulations. Our combined experimental and theoretical approach provides profound insight into the impact of the surrounding environment on the orientation of single molecules. This work demonstrates control over a range of stable spatial orientations of single molecules chemically organized on functional DNA origami templates.

## 2. Results and Discussion

### 2.1. Template Design and Orientation Measurements

**Figure 1:**
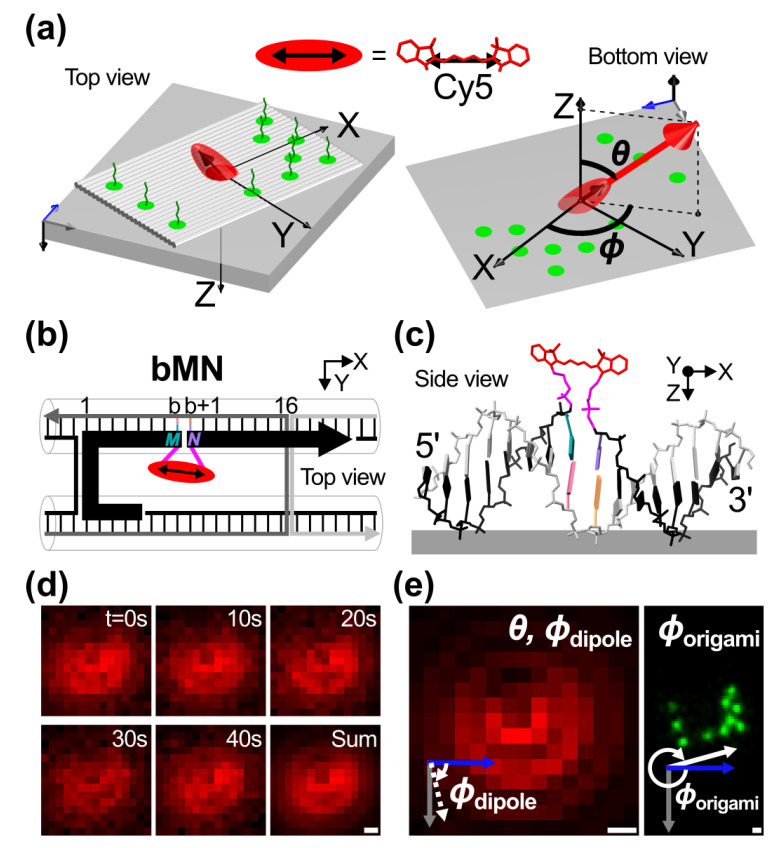
Spatial orientation of a single Cy5 relative to the DNA origami template using dipolar imaging and DNA-PAINT. (**a**) Left: schematic of the template on the glass substrate. Red ellipsoid and green circles represent Cy5 and docking sites for DNA-PAINT imagers, respectively. The TDM of the dye is depicted as the double-headed arrow inside the ellipsoid. Right: Bottom view through the glass. The TDM orientation is given by the polar (***θ***) and azimuthal (***ϕ***) angles relative to the template. (**b**) Inset of the design of the template. A segment of the scaffold (thin black strand) is replaced by a Cy5-internally-labeled DNA (thick black strand) that is complementary to the dark grey staple. Cy5 is placed b number of bases from a common crossover. The position of the Cy5 was changed by tuning the sequences of the dark and light grey staples while keeping the same bases, including the **M** and **N** flanking bases. A general sample was named **bMN**. The replaced segment of the scaffold is not shown. (**c**) Schematics of the molecular structure of the dye tethered to the DNA duplex on glass. Both tethers are shown in pink. Neighboring bases are shown in different colors. (**d**) Representative frames of the dipole radiation pattern of **8TT** (**b** = **8**, **M** = **T**, **N** = **T**). Each frame was acquired for 2 s. Frames were summed to improve the signal-to-noise ratio. (**e**) Left: Simulated dipole radiation pattern with ***θ***_dipole_ (=35°) and ***ϕ***_dipole_ (=77°) at 550 nm defocused distance obtained after fitting the summed frame in (**d**). Right: DNA origami orientation, ***ϕ***_origami_ (= 345°), was obtained relative to the camera coordinates after DNA-PAINT reconstruction. The dye orientation was obtained as ***θ*** = ***θ***_dipole_ and ***ϕ*** = ***ϕ***_dipole_-***ϕ***_origami_. The Cartesian coordinates of the origami and glass are the XYZ axes and the unlabeled blue, grey, and black arrows. Scale bars for the images of dipole radiation patterns and DNA-PAINT reconstruction are 200 nm and 10 nm, respectively.

We designed a DNA template, building upon a recently published sheet-like, rectangular DNA origami [18] (see Methods). The template was designed to allow independent super-resolution microscopy (SRM) in the green channel and defocused dipole imaging (DDI) Cy5 in the red channel (Figure 1a). For the SRM design, docking sites for DNA-PAINT using Cy3B imagers were arranged in an asymmetrical pattern (green protruding single-stranded DNAs). When physisorbed onto a glass surface, the origami laid flat with the docking sites exposed to the buffer solution.

For the DDI design, a single Cy5 and its TDM are represented by a red ellipsoid and a double-headed arrow, respectively. The Cy5 was covalently attached via two tethers to the backbone of a single-stranded DNA, simply called strand hereinafter, belonging to a DNA duplex in the origami. These two tethers are defined in this work as connecting the Cy5′s nitrogen atoms to the carbon atoms of the sugars. Tethers consist of three-carbon C3 length linkers used for attachment, phosphates, and an extra carbon at the 3′ end (see the two tethers in pink in Figure 1c and Figure S1). Hereinafter, the spherical coordinate system used to represent the dye orientation is defined with respect to the Z-axis protruding through the bottom of the glass as viewed by the objective of the inverted microscope (Figure 1a, right).

A 16 base pairs DNA duplex, where the dye was tethered, was chosen in the vicinity of the seam of the DNA origami nanostructure (Figure 1b). The seam is the place in the middle of the origami where the scaffold crossovers of the origami are “touching” each other and staples are “bridging” over those crossovers [25]. In our design, a segment of the scaffold in the seam was replaced by a DNA strand internally functionalized with a Cy5 (black thick strand, whereas the scaffold is depicted as the black thin lines in Figure 1b. The replaced segment of the scaffold is not shown). We ensured that the replaced segment of the scaffold did not share sequences with the Cy5-tethered strand to prevent strand displacement. Dye positions were tuned by changing the sequence composition of two staple strands (dark and light grey staples in Figure 1b). In this way, different tethering positions along the helical structure of a DNA duplex were compared (side view in Figure 1c), while keeping the same nucleic acid bases surrounding the dye constant for any position.

The identity of the neighboring bases and the interference due to the glass, crossovers, or duplexes in the vicinity of the dye are potential factors that may affect the orientation of the dye as suggested by our preliminary experiments (see Text S1). To reduce such potential factors, we maintained the first neighboring bases surrounding the dye and selected positions far from the glass and in the middle of the duplex to begin our study. In general, six different positions spaced a distance **b** (= **5**, **6**, **7**, **8**, **9**, **10**) number of base pairs from a common crossover were selected as they represent a half-turn of the DNA duplex. In these positions, the effect of the glass on the dye attached to the flat origami was considered negligible. We used the nomenclature **bMN**, where **M** and **N** were the first neighboring bases or flanking bases of the dye at the 5′ and 3′ ends, respectively while maintaining the second, third, fourth, and subsequent neighboring bases (Figure 1b,c). In our experiments, only **TT**, **GC**, and **AA** cases were characterized; T, G, C, and A are the thymine, guanine, cytosine, and adenine bases. These three cases provide a minimal set among the 16 possible cases for the first neighboring bases. The three cases were chosen because they provided different Watson-Crick base pairing energies (A·T base pairing is thermodynamically weaker than G·C), and **AA** had the complementary bases of **TT**. In this way, the environment surrounding the dye was controlled for characterization of its effect on dye orientation control. 

Obtaining the dye’s spatial orientation relative to the origami coordinates required three steps. As an example of these steps, data for **8TT** (**b** = **8**, **M** = **T**, **N** = **T**) are shown in Figure 1d,e. First, the dipole radiation pattern of Cy5 was obtained through defocused fluorescence images taken with an acquisition time of 2 s. We hypothesized that stable orientations can be extracted from measurements within that acquisition time; however, different molecular systems than the ones reported in the present work might require different acquisition times. The time series of a representative radiation pattern (Figure 1d) shows that the dipole maintained its orientation, and, because of this, the dipole radiation pattern could be integrated over time. Frames in the time series were summed from the first frame until before the dye was bleached or before a change in orientation occurred; if a change in orientation occurred, the frames with the new orientation were separately summed (see Figure S3 for a representative time series of a dye changing orientation). This step helped to increase the signal-to-noise ratio. The summed frame was used to reconstruct the TDM in spherical coordinates in the frame reference system (***θ*** and ***ϕ***_dipole_ in Figure 1e). Note that the frames captured using an inverted microscope were “mirror” images of what was observed through the glass substrate. Second, the in-plane orientation of the origami relative to the frame (***ϕ***_origami_ in Figure 1e) was measured from the reconstructed DNA-PAINT pattern. Third, the TDM’s arrow coordinates were transformed into the spherical coordinates (***θ***, ***ϕ***) relative to the origami (Figure 1a), where ***ϕ*** = ***ϕ***_dipole_-***ϕ***_origami_. The mean orientation and standard deviation (dispersion hereinafter) of each sample was calculated using the framework of the Fisher-Bingham or Kent distribution for spherical data (see Text S2) [59,60,61].

### 2.2. **θ** Orientation Control via Intercalation and Attachment Position

#### 2.2.1. Effect of the Neighboring Bases

**Figure 2:**
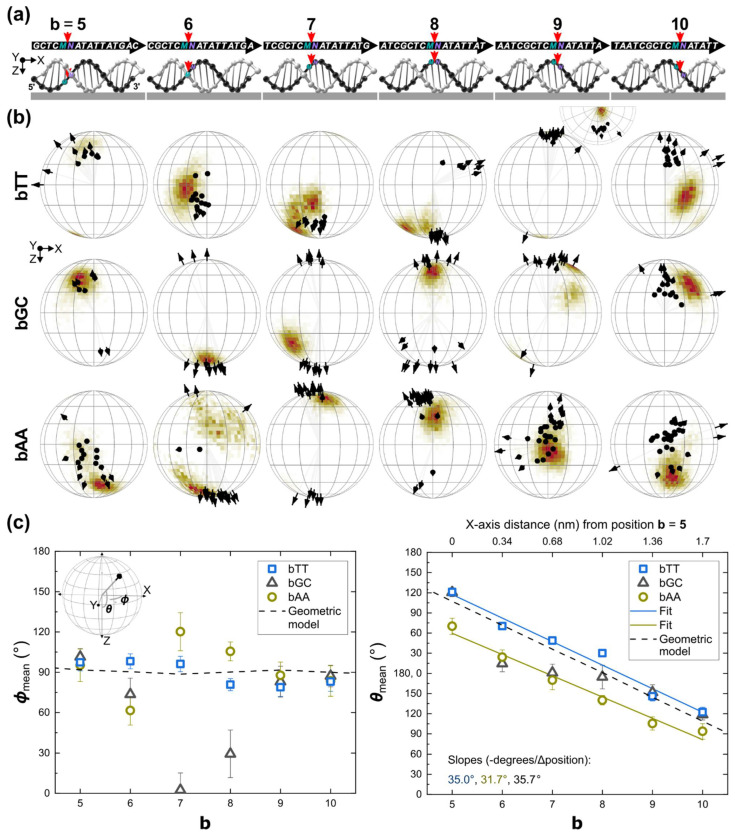
The effect of the neighboring bases on the spatial orientation of Cy5, as a function of the attachment position **b**. (**a**) Schematics of DNA duplex on the glass substrate indicating **b** locations used in the study. DNA sequences are shown on top. Samples are labeled as **bMN**, where **M** and **N** were the dye’s flanking bases at the 5′ and 3′ ends, respectively. The 5′-to-3′ direction of the Cy5-tethered strand runs along the positive X-axis (see Figure 1a). (**b**) Results for **bTT** (top row), **bGC** (middle row) and **bAA** (bottom row). Each column of spheres corresponds to the schematics in (**a**). Experimental orientations are plotted on the unit hemisphere as black arrows. MD simulation results are plotted as histograms on the surface of the hemisphere. (**c**) Plots of ***ϕ***_mean_ vs. **b** and ***θ***_mean_ vs. **b**. The inset on the left shows again the coordinates for convenience. A geometric model (dash lines) was constructed based on the neighboring base pairs in a static DNA duplex (see Text S4.1). Error bars are $\sigma =\pm \sqrt{\sigma_{x*}\sigma_{y*}}$, where $\sigma_{x*}$ and $\sigma_{y*}$ are the standard deviations of the elliptical cone with the center in the mean direction in the framework of the Kent (Fisher-Bingham) distribution [59].

The schematics of the **bMN** samples are shown in Figure 2a illustrating the dye attachment positions **b**, which are far from the glass, and all the bases in the DNA strand. Experimental orientations for **bTT**, **bGC**, and **bAA** are shown as black arrows on the unit hemisphere in Figure 2b top, middle, and bottom rows, respectively. Simulated orientations using MD are shown as colored histograms on the surface. 

We first explain the features in the experimental data of **bTT** (Figure 2b, top row). ***ϕ***_mean_ of **bTT** was close to 90° (empty squares in Figure 2c left. Statistical results are also shown in Table S1), i.e., ***ϕ***_mean_ was approximately perpendicular to the DNA axis. ***θ***_mean_ changed as a function of the attachment position at 35.0° per base (Figure 2c right and Table S2), which was close to the experimental DNA twist angle of 35.4° [62], indicating that **bTT** followed the helical and periodical nature of DNA in its B-form. As a result, ***θ*** orientation control was achieved in a stepwise manner for a full revolution within a distance of 1.7 nm as determined by the range of the different attachment positions in the X-axis (from **b** = **5** to **10**. Figure 2c right). We compared **bTT** to a geometric model constructed from the molecular coordinates of B-form DNA (see Text S4). In this model, the orientation of each of the two neighboring base pairs was calculated by using the carbons of the respective sugars, and then the mean orientation was calculated and given as ***θ***_model_ = 285.7° − 35.7° × **b** and ***ϕ***_model_ ≈ 90° (plotted as black dash lines in Figure 2c). The linear fit of ***θ***_mean_ was shifted at most 13.9° (**b** = **10**) towards the north of ***θ***_model_, whereas ***ϕ*_bTT_** = 89.1° ± 9.1° and ***ϕ***_model_ were both ~90°. 

The results for **bGC** and **bAA**, i.e., results with different first neighboring base pairs, had the following features. The mean orientations of **bGC** and **bAA** were perpendicular to the DNA axis (empty triangle and empty circle in Figure 2C, left). Note that the orientations that are close to the poles, i.e., ***θ*** = 0° or 180°, can lead to ***ϕ***_mean_ values that are far from 90°, as obtained for **7GC**, **8GC**, **6AA**, and **7AA** (Figure 2B, middle and bottom rows). **bGC** had a trend that resembles **5TT**, **6AA**, **7AA**, **9TT**, and **10TT**, and it had the features of both **8TT** and **8AA**. **bAA** had a periodical trend similar to **bTT** (empty circles in Figure 2c, right), but with a rotational shift of 40.8° towards the south of **bTT**. ***θ***_mean_ of **bAA** changed as a function of the attachment position at 31.7° per base (see Table S2), which was again close to the DNA twist angle and the slope of **θ**_mean_ of **bTT**. The fact that **bTT** was exceptionally close to the geometric model strongly suggested that the dye orientation depended on the orientation of the first neighboring base pairs. 

Dispersion in the orientation is a measure of the extent of orientation control for a particular measured orientation. Both **bAA** and **bGC** were in general more dispersed than **bTT**. The smallest dispersions (σ) were ±4.5° (for the south pole cluster with orientation ***θ*** = 30.0° ± 4.5°, ***ϕ*** = 80.8° ± 4.5° in **8TT**), ±6.2° (**5GC**) and ±7.0° (**8AA**), and the largest dispersions were ±17.6° (**8GC**) and ±14.2° (**7AA**). The calculation of orientation dispersion included dyes changing orientation, i.e., the dispersion accounted for orientation stability. Although **bTT** and **bGC** had occasionally dyes changing orientation in real time, **bAA** had clearly more dyes changing orientation. For instance, a single dye in **6AA** had four different orientations throughout the experiment (time series is shown in Figure S3). 

We theoretically studied the structural interactions and energetics between Cy5 and DNA bases. The closeness of **bTT** to the geometric model led us to speculate that the neighboring bases allowed the dye to remain intercalated in the tight nanoscale environment between the flanking bases, whereas **bAA** was also intercalated but with a different angle towards the south. As the only difference between **bTT** and **bAA** were the first neighboring bases, we theoretically investigated the interaction between Cy5 and the first neighboring bases using DFT calculations (see Methods). DFT showed that the indole of Cy5 stacked in parallel to T and A bases with interaction or stacking energies of −13.66 kcal mol^−1^ and −14.93 kcal mol^−1^, respectively; however, other stacking configurations different than parallel stacking were favored by C and G bases with stacking energies of −19.31 kcal mol-1 and −18.41 kcal mol^−1^, respectively (see Figure S4 and Table S3). The parallel stacking of the indole with A and T bases suggested that the dye might be “sandwiched” and trapped into stable microstates depending on the spatial arrangement of the T and A bases, leading to the different orientations observed for **bTT** and **bAA**. As dyes occasionally changed orientation, we speculated that the transition between stable microstates would be promoted by DNA breathing [33,63], base flipping [64], or a mechanism in which the dye and the neighboring bases stack and unstack.

To further investigate the dynamic interaction of Cy5 and neighboring bases for comparison with experimental observations, MD simulations were performed. As the DNA sequences, except for the first neighboring bases, and the attachment positions of the dye were the same, the number of parameters to consider for MD was minimal. Due to the structural limitations imposed by the tethers, MD simulations were initialized with the 5′ ends of Cy5 partially intercalated. In addition, Cy5 was arranged either with its nitrogen atoms pointing to the front or the back of the side view of the DNA helix (see Figure S6). The best matching simulation results are plotted as histograms on the surface of the unit hemispheres in Figure 2b (see Figure S7 for both front and back results plotted on the same hemisphere). In general, excellent agreement was obtained between MD simulations and experiments. The indoles and the neighboring bases are also stacked as shown by the DFT calculations (see representative MD angles and distances in Figure S8). The simulated orientations when the Cy5 nitrogen atoms were in front and back agreed with **bTT** and **bAA**, respectively. Whereas most of the **bGC** were matched by front nitrogen intercalations, **6GC** was matched by back nitrogen intercalations, and **8GC** was matched by both front and back nitrogen intercalations (only back nitrogen intercalation is shown on the respective hemisphere). Interestingly, both **bTT** and **bAA** might have a small amount of back and front intercalation, respectively (see Figure S7). These results suggested that two independent initialization configurations, i.e., front and back nitrogen intercalations, were needed to cover the space of experimental orientations because of the large number of degrees of freedom and trapping microstates between the moieties in the dye-DNA system. These results further support the idea that, in addition to DNA breathing, the identity of the neighboring bases might preferentially favor the orientation of Cy5 in the tight sub-nano-metrical DNA environment.

#### 2.2.2. Effect of the Surface

**Figure 3:**
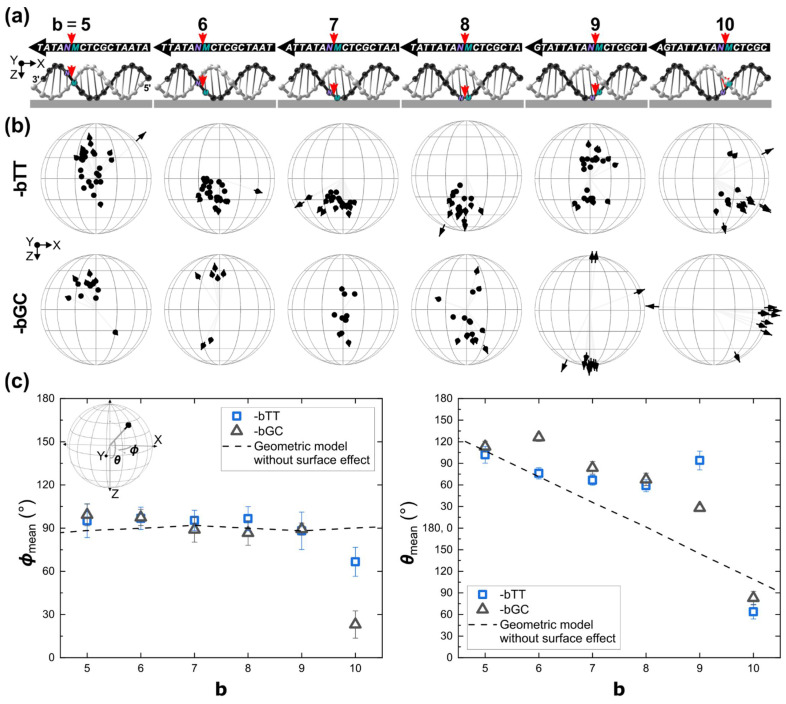
The effect of the glass surface on the spatial orientation of Cy5, as a function of the attachment position **b**. (**a**) Schematics of DNA duplex on the glass substrate indicating **b** locations used in the study. Samples are labeled as **-bMN**, where **M** and **N** were the dye’s flanking bases at the 5′ and 3′ ends, respectively. The negative sign indicates that the 5′-to-3′ direction of the Cy5-tethered strand runs along the negative X-axis (see Figure 1a). (**b**) Results for **-bTT** (top row) and **-bGC** (bottom row). Each column of spheres corresponds to the schematics in (**a**). Experimental orientations are plotted on the unit hemisphere as black arrows. (**c**) Plots of ***ϕ***_mean_ vs. **b** and ***θ***_mean_ vs. **b**. The inset on the left shows again the coordinates for convenience. A geometric model (dash lines) was constructed based on the neighboring base pairs in a static DNA duplex without any effect from the surface (see Text S4.1). Error bars are $\sigma =\pm \sqrt{\sigma_{x*}\sigma_{y*}}$, where $\sigma_{x*}$ and $\sigma_{y*}$ are the standard deviations of the elliptical cone with the center in the mean direction in the framework of the Kent (Fisher-Bingham) distribution [59].

We also evaluated the effect of the surface on the orientation of the dye by taking advantage of the modularity of our DNA origami design and putting the Cy5 in the complementary strand while keeping the same first neighboring bases (see Figure 3 and Figure S9 for the design of the template). These positions are labeled as **-bMN**, where the negative sign indicates that the 5′-to-3′ direction of the Cy5-tethered strand runs along the negative X-axis. Schematics of **-bMN** depicting the dye attachment positions near the glass and all the nucleic acid bases in the DNA strand are shown in Figure 3a. 

Experimental data of **-bTT** and **-bGC** are shown in Figure 3b. ***ϕ***_mean_ was close to 90° for **b** = **5** to **9** and in agreement with a geometric model that did not consider any effect from the surface (dash lines in Figure 3b. Statistical results are also shown in Table S1). These results strongly confirmed the intercalation of Cy5 as demonstrated in Section 2.2.1 for positions far from the glass; however, contrary to positions far from the glass, ***θ***_mean_ vs. **b** did not resemble the characteristic helical trend (see Figure 2c, right) except for **-5TT**, **-6TT** and **-5GC** that were close to the geometric model. ***θ***_mean_ mostly shifted towards the north of the model depending on the value of **b**. As the dye proximity to the surface was also a function of **b**, the shift of ***θ***_mean_ from the model was attributed to a conformational change in the orientation of the intercalated dye induced by the direct physical interaction with the surface. For **-9TT** and **-9GC**, the shift of ***θ***_mean_ was 129.5° and 63.7° respectively. Interestingly, position **b** = **10** had different behavior. **-10TT** and **-10GC** had orientations towards the southeast, indicating a more drastic effect from the surface. Moreover, as ***ϕ***_mean_ was far from 90° (Figure 3c, left), it was possible that the dye was not intercalating between the neighboring base pairs but interacting with the surface of the DNA duplex.

To the best of our knowledge, the partial intercalation of Cy5 within a DNA duplex has not been reported before. Our present results in Section 2.2.1 and Section 2.2.2 were consistent with a previous contribution assessing the in-plane orientation of Cy5 singly tethered (via a C6 linker) to the terminal end of the DNA backbone [37]. In this case, the distribution of the in-plane ***ϕ*** was found to be uniform unless two bases were removed in the vicinity of the dye, in which case the in-plane ***ϕ*** was ~90° [37]. Moreover, the partial intercalation we found was consistent with the theoretical prediction of the intercalation of a doubly tethered Cy3 dye into the space produced by placing one unpaired adenine in the complementary strand [65]. Additionally, the partial intercalation of Cy5 is compatible with a proposed half-intercalation mechanism in which half of a molecule intercalates but the other half interacts with the minor groove [66]. For the tight sub-nanometer environment between the flanking bases in the present work, it is an exceptional finding that the Cy5 orientation was constrained by intercalation. 

### 2.3. **ϕ** Orientation Control via Mechanical Stretching

Given that our previous results described in the preceding sections showed that the orientation of the Cy5 dye was predominantly perpendicular to the DNA axis (for instance, ***ϕ*_bTT_** = 89.1° ± 9.1°), we engineered our DNA template to achieve different ***ϕ*** values. We hypothesized that by mechanically stretching Cy5 via its two tethers, we could direct the in-plane orientation of Cy5. This stretching can be achieved by fixing one end of the Cy5-tether and pulling from the other end. Hereinafter, the subsystem consisting of Cy5 and its two tethers is called Cy5-tether. With a suitable stretching level, the Cy5-tether would behave as a stretched polymer in which the number of microstates decreases, i.e., entropy decreases, and consequently the orientation of Cy5 is controlled by the direction of stretching. 

**Figure 4:**
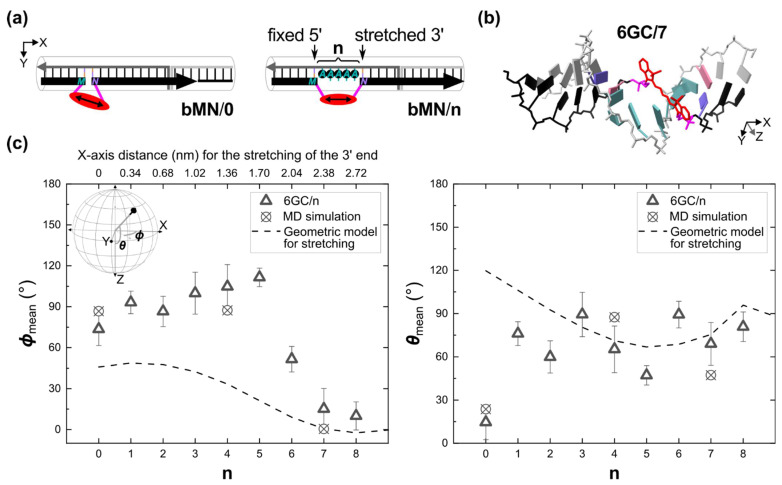
The effect of mechanical stretching on the spatial orientation of Cy5, as a function of the stretching level **n**. (**a**) Schematics for the mechanical stretching using the same DNA template depicted in Figure 1**b**. Stretching was done in a stepwise manner by introducing **n** number of unpaired adenine bases. Samples are labeled as **bMN**/**n**, where **M** and **N** were the dye’s flanking bases at the 5′ and 3′ ends, respectively. The positive sign indicates that the 5′-to-3′ direction of the Cy5-tethered strand runs along the positive X-axis (see Figure 1a). Left: Structure without stretching, **bMN**/**0**. Right: Structure with stretching. (**b**) MD simulation frame of **6GC**/**7**. (**c**) Plots of ***ϕ***_mean_ vs. **n** and ***θ***_mean_ vs. **n** for **6GC**/**n**. The inset on the left shows again the coordinates for convenience. A geometric model (dash lines) was constructed based on the neighboring base pairs in a static DNA duplex (see Text S4.2). MD simulations for **n** = **0**, **4**, and **7** are shown as circles with x. Error bars are $\sigma =\pm \sqrt{\sigma_{x*}\sigma_{y*}}$, where $\sigma_{x*}$ and $\sigma_{y*}$ are the standard deviations of the elliptical cone with the center in the mean direction in the framework of the Kent (Fisher-Bingham) distribution [59].

The Cy5-tether can directly be stretched using our DNA origami template by increasing the distance between the two flanking base pairs by a fixed number of bases (Figure 4). This separation was achieved by letting the Cy5-tether occupy the space of **n** bases and placing **n** unpaired adenine bases in the complementary strand, directly opposing the dye (scheme in Figure 4a). As a proof of concept, we chose position 6GC (data shown in Figure 2. Statistical results are also shown in Table S1). The position of the flanking base pairs in the 5′ end was fixed, while Cy5-tether was stretched by placing the flanking base pairs in the 3′ end at a distance given by n adenines, where **n** = **1** to **8** are the stretching levels. The general notation for the stretching experiment is **6GC**/**n**, where **6GC**/**0** is equivalent to **6GC**. A representative molecular structure for **n** = **7** is shown in Figure 4B, which is a frame from the MD simulation.

The results for different stretching levels are shown in Figure 4c, and the stretching distance along the X-axis is shown on the top axis of Figure 4c, left. The maximum stretching distance was 2.72 nm (**n** = **8**). As expected, stretching directed the orientation of the Cy5 molecule. In general, ***ϕ***_mean_ increased constantly until **n** = **5**, whereas ***θ***_mean_ oscillated. This ***θ***_mean_ vs. **n** oscillation was attributed to the 3‘ end of the Cy5-tether being moved in a step-wise manner along the helical structure of DNA. The tighter dispersion of **6GC**/**1** (***θ*** = 76.0° ± 8.3°, ***ϕ*** = 273.1° ± 8.3°) in the range **n** = **0** to **4** might be because the neighboring bases, which consisted of G:C base pairs, allowing room to accommodate Cy5. A similar **6GC**/**1** case was characterized by Mathur et al. in bulk using fluorescence lifetime and MD for a Cy3 dye [36] (smaller dye than Cy5) obtaining constrained orientations, as in our results, but with a different orientation. In our experiments, the change of orientation between the accommodated **6GC**/**1** and the intercalated **6GC**/**0** (***θ*** = 14.5° ± 12.1°, ***ϕ*** = 253.6° ± 12.1°) was mainly Δ***θ*** = 61.5°, i.e., ***θ*** angle orientation control from the south pole of the hemisphere to the equator was achieved for a stretching distance of ~0.3 nm in the X-axis (from **n** = **0** to **1**). Moreover, we observed an increase in the dispersion of the orientation until **n** = **4**, which can be due to a decrease in the interaction between Cy5 and the flanking bases and simultaneous interaction of Cy5 with the unpaired adenines (see Section 2.2.1 for discussions on similar Cy5-base interactions), whereas **6GC**/**5** had the smallest dispersion (***θ*** = 47.2° ± 6.8°, ***ϕ*** = 291.5° ± 6.8°) among all stretching levels 

***ϕ***_mean_ appeared to be decreasing asymptotically for large stretching levels **n** ≥ **6**, which agreed well with the trend of a geometric model, particularly for **n** = **7** and **8** (dashed lines in Figure 4c left). In this model, the orientation vector starting from the 5‘ end attachment point of Cy5-tether, i.e., a carbon of the respective sugar, to the other 3‘ end attachment point was calculated. For large stretching levels in the model, the ***ϕ***_model_ approached 0° and the ***θ***_model_ remained close to 90°, i.e., the orientation was along the DNA axis (X-axis). These experimental results suggest that for large stretching levels the orientation was governed by the direction of the 3‘ attachment point of Cy5-tether, as intended. The change from the accommodated **6GC**/**1** to the fully stretched **6GC**/**8** (***θ*** = 80.8° ± 10.3°, ***ϕ*** = 190.0° ± 10.3°) was Δ***ϕ*** = −83.1° and Δ***θ*** = 4.8°, i.e., ***ϕ*** angle orientation control from the center of the hemisphere to the east was obtained for a stretching distance of ~2.4 nm in the X-axis (from **n** = **1** to **8**). MD simulations for **n** = **0**, **4**, and **7** (circles in Figure 4c) agreed with our stretching experiments and provided theoretical support to the experimental results.

## 3. Materials and Methods

### 3.1. DNA Origami Fabrication

DNA origami was designed based on a previously published sheet-like rectangular structure (~90 × 70 nm) [18]. One side of the structure had an asymmetric pattern made of docking sites for super-resolution imaging using DNA-PAINT. Docking sites were spaced at least 10 nm apart. In the context of the DNA origami method, short staples fold a long circular scaffold. Docking sites were extended staple strands complementary to the Cy3B imager strand. DNA origami was fabricated by mixing 22  nM M13mp18 scaffold (Bayou Biolabs) with 10× unmodified staples (Integrated DNA nanotechnologies, IDT), 50× extended staples for PAINT (IDT), 50× tunable staples for Cy5 (IDT), 100× Cy5-tethered strand (IDT), 1× TAE (Tris-Acetate-EDTA, Thermo Fisher Scientific), and 18 mM MgCl_2_ (MilliporeSigma, Burlington, MA, USA) in nuclease-free water (Nanopure, Thermo Scientific, Waltham, MA, USA) with a 40 µL total volume. The mixture was annealed in a Mastercycler nexus thermal cycler (Eppendorf, Hamburg, Germany). The annealing protocol involved heating at 90 °C for 1 min, then 2 min at 80 °C, and then cooling from 80 to 25 °C over the course of 12 h. Origami was purified by gel electrophoresis using 1% agarose gel containing 0.5× TBE (Tris-borate-EDTA, Thermo Fisher Scientific) and 8 mM MgCl_2_. Single sharps bands were excised and crushed, and the exudate was collected. Purified origami samples were stored in the dark at 4 °C until use. Table S4 shows all the DNA strands used in the present work.

### 3.2. Glass Substrate Preparation

Borosilicate glass coverslips (22 × 22 mm, #1 Gold Seal Coverglass) were first cleaned by sonication in 0.1% (*v*/*v*) Liquinox (Pollardwater, Inc.) for 1 min, then rinsed and sonicated in ultra-pure water for 1 min, and finally spin centrifuged to remove excess water. Coverslips were kept at 40 °C for at least 30 min. One surface of the coverslip was scratched with a diamond pen. Fiducial markers (0.2 pM AuNPs in methanol, NanoPartz) were deposited onto the coverslips for 10 min at room temperature. Coverslips were rinsed with methanol and ultra-pure water and stored at 40 °C until use.

### 3.3. Chamber Preparation and DNA Origami Physisorption onto Glass Substrate

DNA origami in buffer solution was physisorbed onto glass substrates following a glow-discharge method previously demonstrated [18,52] using a custom glow discharge vacuum chamber [67]. Briefly, glow discharge was applied to prepared coverslips using an electrode coupled 115 V Electro-Technic BD-10A High-Frequency Generator under 2 Torr of vacuum for 75 s. A chamber was made by putting the coverslip on top of a glass slide using double-sided tape to glue them together. The glass slide was rinsed with isopropyl alcohol beforehand. Then, 80 µL of ~10 pM origami diluted in deposition buffer (0.5× TBE, 75 mM MgCl_2_) was injected inside the chamber and incubated for 10 min at ambient temperature. After incubation, the chamber was rinsed three times with 80 µL of deposition buffer. Finally, the chamber was refilled with the imaging buffer containing 0.5× TBE, 75 mM MgCl_2_, 1 mM (±) −6-hydroxy-2,5,7,8-tetra-methylchromane-2-carboxylic acid (Trolox, MilliporeSigma), 100 nM Protocatechuate 3,4-dioxygenase pseudomonas (PCD, MilliporeSigma), 5 mM 3,4-dihydroxybenzoic acid (PCA), and 3 nM of Cy3B PAINT imagers (Bio-Synthesis, Inc., Lewisville, TX, USA). The chamber was sealed and ready for imaging.

### 3.4. Fluorescence Microscopy for Single Dipole Imaging

Single-molecule dipolar imaging was carried out on a Nikon Eclipse Ti2 inverted microscope (NY, USA) with a Nikon CFI Apochromat TIRF 100× oil immersion objective (NA = 1.49). All laser excitation wavelengths were sourced from a Nikon Laser Univ model LUN-F. Focus on the surface was achieved using gold nanoparticles excited at 488 nm, which prevented photobleaching the Cy5 molecules. The stage was then stepped toward the objective using the built-in piezo movement controls for defocused imaging. Cy5 molecules were excited at 640 nm in total internal reflection fluorescence (TIRF) mode (0.26 mW measured after the objective). A quad band excitation filter and beam splitter were used to clean up the excitation source and a quad band emission filter isolated the emission from Cy5 molecules, which was then collected by a Princeton Instruments ProEM HS: 512B-N EMCCD (Trenton, NJ, USA) with an EM gain of 100×. The integration time for each captured frame was 2 s. Frames were acquired for 10 min.

### 3.5. Fluorescence Microscopy for Super-Resolution

Immediately following single-molecule dipole imaging, the DNA origami of the same area were imaged below the diffraction limit of light via DNA-PAINT using the same fluorescence microscope with TIRF illumination. The optical system was reconfigured to image Cy3B PAINT imagers [68] with excitation at 561 nm (0.24 mW measured after the objective). All super-resolution imaging was performed at 300 ms per frame for at least 5000 frames and up to 10,000 frames with an EM gain of 100× and recorded into a stack using the Nikon NIS-Elements (version 5.30.01, Nikon Instruments, Melville, NY, USA) prior to processing and analysis.

### 3.6. Data Processing

The signals emitted by the Cy3B imager (transient binding to the origami’s docking sites) in the DNA-PAINT stack were localized and identified using the ImageJ ThunderSTORM plugin [69]. The localizations were rendered, drift corrected with fiducial markers, and the image reconstructed using the same plugin. The reconstructed image was saved and used for color merging with a master dipole image. The master dipole image template was created by summing up initial frames from the dipole stack. Once merged, the dipoles and respective origami were identified using gold nanoparticles as fiducial markers. Origami orientations were calculated from reconstructed PAINT images, and respective dipoles were extracted by selecting 51 pixels × 51 pixels region of interest and summing up frames until before the dye was bleached or before a change in orientation occurred. Due to the intrinsic difficulty of finding the focal plane of the surface, obtaining the actual defocused distance was an issue. Therefore, the defocused distance and dipole fitting simulation were obtained using a library of dipole images generated from the simulation software (steerableDipoleDetector MATLAB algorithm) developed by Aguet and coworkers [48]. All the dipole fitting simulations are shown in Figures S10–S47 in the Supplementary Materials file.

### 3.7. Density Functional Theory (DFT)

To study the stacking of the DNA bases with the Cy5 dye and calculate complexation energies, a first-principles density functional theory (DFT) calculation was deployed using the Gaussian16 software package [70]. Initial dye-base complexes were constructed using UCSF ChimeraX molecular visualization software [71]. To reduce computational load and focus only on the interaction between the dye’s indolenine and base, dimers were formed that consisted of a single base and a Cy5 dye. Similar methods have been employed in other studies to elucidate the stacking configurations and energies of DNA bases and small molecules [72,73,74,75]. For each complex, the base was initially placed above one indolenine at a distance of about 3.5 Å. To test multiple different dye-base orientations, the dye was rotated in plane to four different positions in a roughly 60° arc. For each dye position, the dye was also flipped along its long axis, resulting in eight different dye-base configurations for each base (32 total structures). Each complex was fully relaxed in the ground state to a residual force of 4.5 × 10^−4^ Hartree Bohr^−1^ with the ωB97XD functional [76] and 6-31 + g(d,p) basis set. Ground state frequency calculations were performed to verify that the structures were at true minima by ensuring a lack of imaginary frequencies. The calculations of complexation energies accounted for basis set superposition error by employing the counterpoise method [77,78].

### 3.8. Molecular Dynamics (MD)

MD simulations of the dye-DNA structure were conducted using the GROMACS 2020.3 software package [79]. Structures were constructed using the UCSF ChimeraX [71]. The OL15 force-field [80] with non-bonded modifications [81] was used for the DNA with parameters from GAFF for the Cy5 dye [82]. Atomic partial charges for Cy5 were calculated using the HF/6-31+G* theory level [83]. A truncated 16 base pairs DNA duplex was used to approximate the local environment around the dye. The dye-DNA structures were solvated in TIP3P water in a triclinic box with 1.2 nm between the dye-DNA and box edges. A salt concentration of 75 mM MgCl_2_ was used (the same concentration as in the experiments). Nearest-neighbor searching was used with a 1.2 nm cutoff and Van der Waals interactions were limited to 1.2 nm. Particle Mesh Ewald electrostatics were used with a cutoff of 1.2 nm. Covalent bonds involving hydrogen atoms were constrained with the LINCS algorithm [84] and a timestep of 2 fs was used. See Figure S6 for the initial dye-DNA structures. The dye-DNA structures were first energy minimized using the steepest decent method for 10,000 steps. After energy minimization, the systems were equilibrated in two steps for 1 ns each in the NVT ensemble with harmonic restraints of 1000 kJ mol^−1^ nm^−2^ imposed on heavy atoms for the first step and 100 kJ mol^−1^ nm^−2^ for the second. A third equilibration step was conducted in which all harmonic restraints were removed, except for those on terminal C1′ atoms, which were kept at 50 kJ mol^−1^ nm^−2^ to simulate the DNA being restrained to the rest of the DNA origami construct (similar to a previous study [37]). After equilibration, 500 ns production simulations were run in the NPT ensemble with 50 kJ mol^−1^ nm^−2^ restraints on the terminal C1′ atoms. The velocity-rescale thermostat [85] was used to keep the temperature at 300 K with a coupling time of 0.1 ps. The pressure was kept at 1 atm using the Parrinello-Rahman [86] barostat with a pressure coupling time of 1.0 ps.

## 4. Conclusions

Our work extended the toolbox of DNA nanotechnology towards enhanced nanoarchitectural control of single molecules. By rationally designing DNA origami templates, through which the molecular environment was tuned, we devised two strategies to achieve control over the spatial orientation of Cy5 single molecules. In the first strategy, polar angle (***θ***) control with dispersion as small as ±4.5° (**8TT**) was demonstrated by placing Cy5 along a DNA half-turn, i.e., within a distance of 1.7 nm along the DNA double helix, and limiting the effect of the surrounding environment, e.g., by maintaining the same neighboring bases and avoiding the interaction with the immobilizing surface and nearby DNA molecules. We found that the orientations were stable, and this permitted us control over a full revolution in ***θ***. As the in-plane angle (***ϕ***) was perpendicular to the DNA axis (for instance, ***ϕ*** = 89.1° ± 9.1° for **bTT**), we proposed the partial intercalation of Cy5, which was supported by MD and DFT. Interestingly, we found that changing the identity of the first neighboring bases from **TT** to **AA** neighboring bases did affect ***θ*** and change the dispersion. We further confirmed the partial intercalation of Cy5 by the impact of the immobilizing surface on the ***θ*** angle orientation. In the second strategy, in-plane angle (***ϕ***) control with a dispersion of ±10.3° (**n** = **8**) was directed by the mechanical stretching of Cy5 via its two linkers for a maximum stretching distance of 2.72 nm.

Both intercalation and stretching strategies we presented for orientation control are of general applicability. For the intercalation strategy, the molecule would need to have a moiety such as an indole to allow for intercalation with the flanking bases. For the stretching strategy, any doubly tethered single molecule can be used. Future work will be on building circuits [87] of single molecules organized in diverse configurations, in which orientation control and small dispersion, as demonstrated here or in future studies addressing different molecular structures, are paramount for the performance of applications, including exciton delocalization [88,89,90,91] for quantum information science [11]. Another direction can be on optically monitoring the conformational dynamics [92] of natural and artificial nanostructures, e.g., DNA-based structures for molecular robotics [93,94,95]. Moreover, the present insights and methodologies can be useful for understanding molecular interactions. For instance, biomolecular recognition events using DNA aptasensors [16], and the interaction between single molecules positioned near a functional surface [96,97] could be investigated. Finally, our experimental and modeling methodology can be useful for investigating the interaction of molecular components tethered to soft matter systems based on polymers such as in the emerging fields of RNA [98] and protein [99] origami.

## Data Availability

The data presented in this study are contained in the Main Text and Supplementary Materials.

## Supplementary Materials

The following supporting information can be downloaded at: https://www.mdpi.com/article/10.3390/ijms23147690/s1.
