# Supplementary material for: Strategies for Controlling the Spatial Orientation of Single Molecules Tethered on DNA Origami Templates Physisorbed on Glass Substrates: Intercalation and Stretching
Source: Int J Mol Sci. 2022 Jul 12;23(14):7690. doi: 10.3390/ijms23147690 (PMC9323263; doi:10.3390/ijms23147690)
Supplement: Supplementary file 1 [file ijms-23-07690-s001.zip › ijms-1796782-supplementary.pdf]

Supplementary Materials:
Strategies for Controlling the Spatial Orientation of Single Molecules Tethered on DNA Origami Templates Physisorbed on Glass Substrates: Intercalation and Stretching

Keitel Cervantes-Salguero [1,*] *Austin Biaggne* [1] *John M. Youngsman* [1] *Brett M. Ward* [1] *Young C. Kim* [2] *Lan Li* [1,3] *John A. Hall* [4] *William B. Knowlton* [1,5] *Elton Graugnard* [1,3] *Wan Kuang* [5,*]

[1] Micron School of Materials Science and Engineering, Boise State University, Boise, ID 83725, USA
[2] Materials Science and Technology Division, U.S. Naval Research Laboratory, Code 6300, Washington, D.C. 20375, USA
[3] Center for Advanced Energy Studies, Idaho Falls, ID, USA
[4] Division of Research and Economic Development, Boise State University, Boise, ID, USA
[5] Department of Electrical and Computer Engineering, Boise State University, Boise, ID, USA
* Correspondence: keitelcervantess@boisestate.edu, wankuang@boisestate.edu

Keywords: *DNA origami, nanoarchitectonics, single molecules, orientation control, dipolar imaging, super resolution microscopy, DNA-PAINT, intercalation, mechanical stretching, cyanine, Cy5*

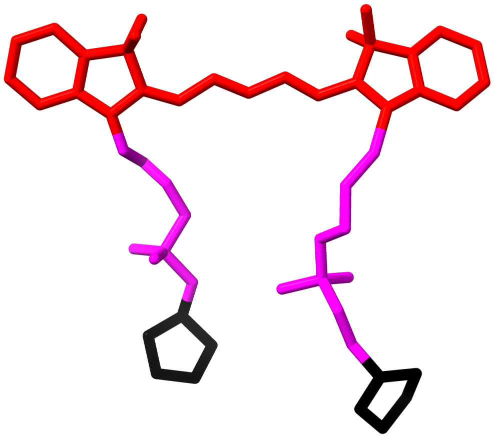

Figure S1: The molecular structure of Cy5 and the two tethers. Cy5 dye (red), tethers (pink) and DNA sugars (black). Whereas both tethers consisted of one C3 linker and one phosphate, the tether on the 3' end of the Cy5 (the tether on the right) had an additional carbon.

Text S1 Rationale of the design: orientation-dependence, neighboring bases and surface

We discuss a thought experiment on the position-dependent orientation of a single molecule attached to a long rigid DNA duplex frozen in space. In particular, the single molecule is attached between two consecutive DNA bases in the center of the duplex. It is assumed that all the bases are the same. Here, we further assume that the single molecule will have a range of orientations with a mean angle with respect to the DNA duplex axis (relative angle). This assumption implies that a local minimum exists for the interaction of the single molecule and DNA. If the single molecule is placed one base after, its relative angle will remain the same; the same happens if the single molecule is placed one base before. As a result, the relative angle of the single molecule is independent of the attachment position on the DNA. Because an angle twist per base ($\sim 35.4°$ in B-form)[1] is present relative to the DNA axis, the position-dependent orientations must follow a helical trend.

The previous analysis assumed that all the neighboring bases were exactly the same and no effect resulted from the immobilizing substrate, which is not the case in reality. We did an experiment that suggested that the neighboring bases had an effect on the orientation. In this experiment, Cy5 was simply attached to one staple strand of the origami, i.e. the DNA sequence was fixed and therefore the neighboring bases of the Cy5 were always changing (see S2).

In the results, $\boldsymbol{\phi}_{\text{mean}}$ was also approximately perpendicular to the DNA axis, which strongly suggested intercalation; however, $\boldsymbol{\theta}_{\text{mean}}$ had a trend that did not follow a clear pattern but oscillated around a geometrical model (see Main Text and Text S4.1). This range of orientations suggested a dependence on the neighboring bases. Moreover, the effect of the immobilizing surface might be observed for $\mathbf{b} = \mathbf{7}$ to $\mathbf{10}$ but this effect was not completely clear as the neighboring bases were different. Nevertheless, these results prompted us to design the DNA platform to keep the same neighboring bases and prevent any effect due to the surface.

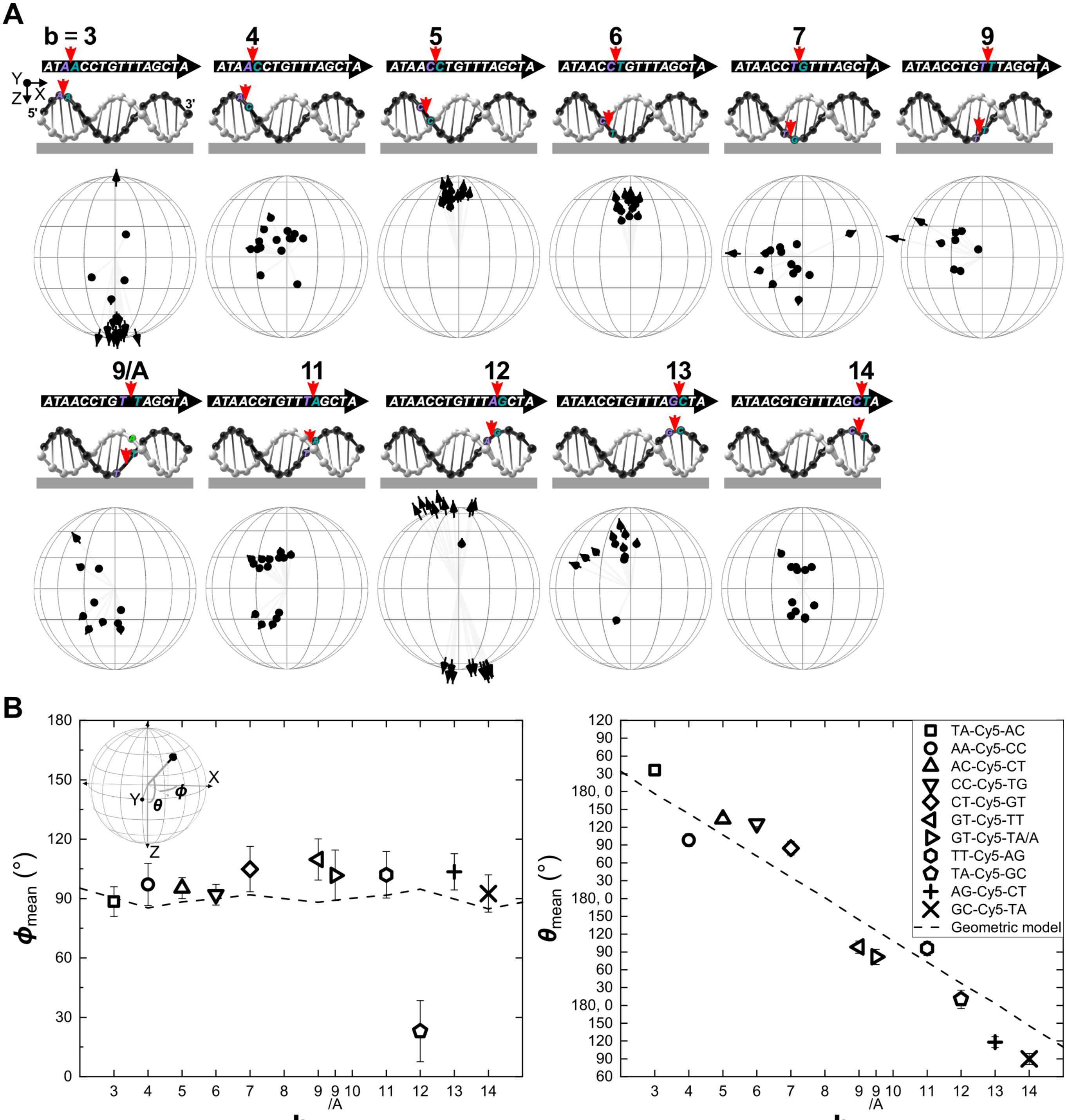

Figure S2: Spatial orientation of Cy5 when attached to a DNA duplex with a fixed sequence. Neighboring bases were different at each attachment position **b**. A) Experimental orientations are plotted on the unit hemisphere as black arrows. Schematics of DNA duplex indicating **b** are plotted on top of each hemisphere. The sequence of the strand in black was 5'-ATAACCTGTTTAGCTA-3'. B) Plots of $\boldsymbol{\phi}_{\mathrm{mean}}$ vs **b** and $\boldsymbol{\theta}_{\mathrm{mean}}$ vs **b**. The inset on the left shows the coordinates for convenience. A geometric model (dash lines) was constructed based on the neighboring base pairs in a static DNA duplex without any effect due to the surface (see Text S2). Error bars are $\sigma = \pm\sqrt{\sigma_{x^*}\sigma_{y^*}}$, where σ_{x^*} and σ_{y^*} are the standard deviations of the elliptical cone with center in the mean direction in the statistical framework of the Kent (Fisher-Bingham) distribution.

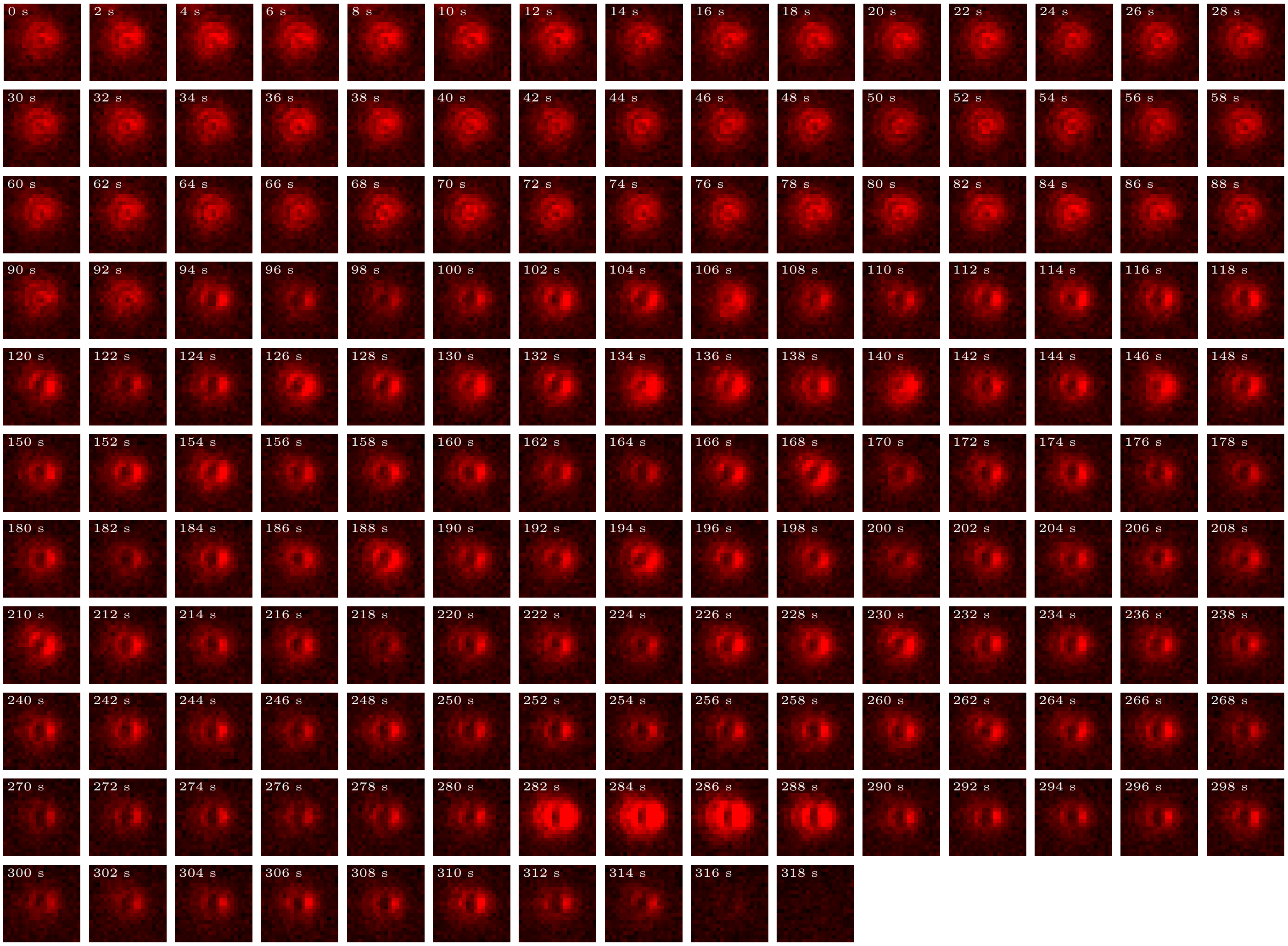

Figure S3: Representative time series of a dipole changing orientation in sample **6AA**. Time evolution runs from the top-left frame to the bottom-right frame. Each frame was captured for 2 sec. The different orientations are observed at 0 s (until 92 s), 104 s (again at 138 s), 112 s, 272 s (until 276 s, and again at 282 s). Dye was bleached completely in the last frame.

Text S2 Kent distribution

The spherical data were analyzed in the framework of the Kent (Fisher-Bingham) distribution[2, 3] which is a spherical analogue of the general bivariate normal distribution in the plane. The Kent distribution can model asymmetric data as oval contours. A confidence region can be obtained as an elliptical cone to represent the dispersion of the data. The Kent distribution is defined by the parameters κ, β, and Γ, which represents the concentration, describes the ovalness, and represents the orientation and axial components of the Kent distribution. These parameters can be estimated by the moment estimates $\tilde{\kappa}$, $\tilde{\beta}$ and $\tilde{\Gamma}$ using an iterative method. In particular, $\tilde{\Gamma}$ is used to find the elliptical cone. The mathematical treatment was described in the original article by Kent[2] but a simplified description in the context of our measurements is described as follows.

Consider the Cartesian and Spherical coordinates as in Figure 1A in the main text. The sample mean vector of the transition dipole moments $\boldsymbol{\mu}_i = [x_i, y_i, z_i]$ ($i = 1$ to n) was calculated as $\bar{\boldsymbol{\mu}} = n^{-1} \sum \boldsymbol{\mu}_i$. The spherical coordinates of the unit vector $\hat{\boldsymbol{\mu}} = \bar{\boldsymbol{\mu}}/\|\bar{\boldsymbol{\mu}}\| = [x_{\text{mean}}, y_{\text{mean}}, z_{\text{mean}}]$ were obtained from:

$$x_{\text{mean}} = cos(\boldsymbol{\phi}_{\text{mean}})sin(\boldsymbol{\theta}_{\text{mean}})$$
$$y_{\text{mean}} = sin(\boldsymbol{\phi}_{\text{mean}})sin(\boldsymbol{\theta}_{\text{mean}})$$
$$z_{\text{mean}} = cos(\boldsymbol{\theta}_{\text{mean}})$$

The sample dispersion matrix was calculated as $\mathbf{S} = n^{-1} \sum \boldsymbol{\mu}_i \boldsymbol{\mu}_i^T$.

The general idea was to properly rotate all the $\boldsymbol{\mu}_i$ vectors along the $\bar{\boldsymbol{\mu}}$, which became a new axis. And then determining variances related to the two orthogonal axes perpendicular to the new axis. This was done in three steps.

The first step was a transformation that rotated all $\boldsymbol{\mu}_i$ in such a way that $\bar{\boldsymbol{\mu}}$ was aligned to a new Z* axis. This transformation was done by the orthogonal rotational matrix H:

$$H = \begin{bmatrix} cos(\boldsymbol{\theta}_{\text{mean}})cos(\boldsymbol{\phi}_{\text{mean}}) & -sin(\boldsymbol{\phi}_{\text{mean}}) & cos(\boldsymbol{\theta}_{\text{mean}})cos(\boldsymbol{\phi}_{\text{mean}}) \\ cos(\boldsymbol{\theta}_{\text{mean}})sin(\boldsymbol{\phi}_{\text{mean}}) & cos(\boldsymbol{\phi}_{\text{mean}}) & sin(\boldsymbol{\theta}_{\text{mean}})sin(\boldsymbol{\phi}_{\text{mean}}) \\ -sin(\boldsymbol{\theta}_{\text{mean}}) & 0 & cos(\boldsymbol{\theta}_{\text{mean}}) \end{bmatrix} \tag{1}$$

The second step was a transformation that rotated all $\boldsymbol{\mu}_i$ around Z* and aligned the axes of large and small dispersion to the new X* and new Y* axis, respectively. This transformation was done by diagonalizing the upper submatrix B_U (2×2) of $B = H^T S H$:

$$B_U = \begin{bmatrix} b_{11} & b_{12} \\ b_{21} & b_{22} \end{bmatrix} \tag{2}$$

ψ was chosen so $\psi = arctan(2b_{12}/(b_{11} - b_{22}))/2$, and the rotation matrix K along the Z* was:

$$K = \begin{bmatrix} cos\psi & -sin\psi & 0 \\ sin\psi & cos\psi & 0 \\ 0 & 0 & 1 \end{bmatrix} \tag{3}$$

Then $\tilde{\Gamma} = HK$.

The third step was determining an ellipse-like region on the unit sphere. This ellipse was defined by the sample moments σ_{x*} and σ_{y*}:

$${\sigma_{x^*}}^2 = n^{-1} \sum x_i^{*2}$$
$${\sigma_{y^*}}^2 = n^{-1} \sum y_i^{*2}$$

Where the coordinates in the new X*Y*Z* axes were transformed: $[x_i^*, y_i^*, z_i^*] = \tilde{\Gamma}^T [x_i, y_i, z_i]^T$.

Finally, $\sigma = \sqrt{\sigma_{x^*} \sigma_{y^*}}$ was the radius of a circular cone with the same area as the elliptical cone in [4, 2]. In our calculation of $\hat{\boldsymbol{\mu}}$ and σ, outliers were not considered.

Text S3 Cartesian coordinate system

To calculate the Cartesian coordinate system (XYZ axes) for the 16 base pairs DNA duplex, the following criteria were used. Calculating the X axis was done by obtaining the center axis of the DNA duplex helix. To calculate the Y axis, we considered the fact that the plane containing the flat DNA origami also contained the DNA duplex and, therefore, the Y axis belonged to this plane and was perpendicular to the center axis of the DNA duplex. Moreover, the DNA origami structure imposed a positional limitation at the two crossovers of both terminal ends of the DNA duplex. These two crossovers were at the 5' end of one strand and at the 5' end of the complementary strand. In addition, the DNA duplex could rotate around the center axis of the DNA duplex but this rotation was limited by the two crossovers; as a result, the DNA duplex was symmetrically positioned relative to the two crossovers, i.e., to the 5' ends of the DNA duplex. The previous structural considerations meant that, in our DNA duplex system, to calculate the Y axis we needed to find the plane that passed through the center axis of the DNA duplex and was satisfying a symmetrical relationship with the terminal ends of the DNA duplex, as seen in Figure 1C in the main text. Once both X and Y axes were defined, calculating the Z axis was simply achieved by obtaining the vector perpendicular to both the X and Y axis. The preceding criteria for finding the XYZ axes was applied to our geometrical model and molecular dynamic simulations. In our calculations, the DNA bases were represented by the C1' carbon atoms of the DNA duplex. C1' is the carbon connecting the DNA base to the sugar of the backbone. In the molecular dynamic simulations, the XYZ axes were recalculated at every simulation step.

Text S4 Geometric models

Text S4.1 Model for position-dependence

A DNA model was built based on a 16 base pairs DNA in B-form. The atomic positions were obtained from Protein Data Bank (PDB) data. In this model, the positions of the bases were simplified by representing them by the position of the C1' carbon atoms of the respective sugars of the DNA backbone. The Cartesian coordinate system for the DNA duplex was calculated as in Text S3 (see Figure 1A in the main text for schematics of the coordinates). In the model, the vector orientation for a flanking base pair was calculated by connecting the respective C1' carbons of each of the two bases in the base pair. The vector orientations were obtained for both two flanking base pairs. Then, the mean orientation of the two vectors was calculated. The orientation of the model as a function of b was given by $\boldsymbol{\theta}_{\mathrm{model}} = 285.7^\circ - 35.7^\circ \times \mathbf{b}$, and $\boldsymbol{\phi}_{\mathrm{model}} \approx 90^\circ$ (perpendicular to the DNA axis for any position).

Text S4.2 Model for stretching

In similar way to Text S4.1, a DNA model was built based on a 16 base pairs DNA in B-form. This time, the vector orientation from one flanking base to the other flanking base was calculated for all stretching levels.

Table S1: Statistical results for all samples in the Main Text: **bTT**, **bGC**, **bAA**, **-bTT**, **-bGC**, and **6GC/n**

Sample	θ_{mean}	ϕ_{mean}	σ_{x^*}	σ_{y^*}	σ
**5TT** (5'-TCT-Cy5-TAT-3')	121.2°	97.4°	7.6°	5.1°	6.2°
**6TT** (5'-TCT-Cy5-TAT-3')	70.4°	98.2°	7.0°	4.4°	5.6°
**7TT** (5'-TCT-Cy5-TAT-3')	48.8°	96.1°	8.2°	4.0°	5.7°
**8TT** (5'-TCT-Cy5-TAT-3')	30.0°	80.8°	5.5°	3.6°	4.5°
**9TT** (5'-TCT-Cy5-TAT-3')	145.8°	78.8°	9.4°	5.0°	6.9°
**10TT** (5'-TCT-Cy5-TAT-3')	122.3°	83.0°	9.8°	5.3°	7.2°
**5GC** (5'-TCG-Cy5-CAT-3')	120.4°	101.4°	8.5°	4.5°	6.2°
**6GC** (5'-TCG-Cy5-CAT-3')	14.5°	73.6°	14.9°	9.8°	12.1°
**7GC** (5'-TCG-Cy5-CAT-3')	1.0°	2.5°	24.0°	6.8°	12.7°
**8GC** (5'-TCG-Cy5-CAT-3')	174.5°	29.3°	23.9°	13.0°	17.6°
**9GC** (5'-TCG-Cy5-CAT-3')	151.5°	83.0°	14.2°	9.3°	11.5°
**10GC** (5'-TCG-Cy5-CAT-3')	118.7°	87.2°	11.4°	5.6°	8.0°
**5AA** (5'-TCA-Cy5-AAT-3')	70.1°	95.2°	15.0°	9.71°	12.1°
**6AA** (5'-TCA-Cy5-AAT-3')	24.1°	61.5°	15.9°	7.4°	10.8°
**7AA** (5'-TCA-Cy5-AAT-3')	169.9°	120.2°	21.2°	9.5°	14.2°
**8AA** (5'-TCA-Cy5-AAT-3')	140.0°	105.5°	8.6°	5.7°	7.0°
**9AA** (5'-TCA-Cy5-AAT-3')	105.4°	87.7°	16.1°	6.2°	10.0°
**10AA** (5'-TCA-Cy5-AAT-3')	93.8°	83.5°	14.9°	8.7°	11.4°
**-5TT** (3'-TAT-Cy5-TCT-5')	101.8°	95.0°	16.2°	8.3°	11.6°
**-6TT** (3'-TAT-Cy5-TCT-5')	76.2°	96.8°	9.6°	6.2°	7.7°
**-7TT** (3'-TAT-Cy5-TCT-5')	66.5°	95.4°	8.7°	5.7°	7.0°
**-8TT** (3'-TAT-Cy5-TCT-5')	59.0°	96.6°	10.6°	6.7°	8.4°
**-9TT** (3'-TAT-Cy5-TCT-5')	94.0°	88.1°	20.2°	8.4°	13.0°
**-10TT** (3'-TAT-Cy5-TCT-5')	63.8°	66.6°	10.6°	9.7°	10.1°
**-5GC** (3'-TAC-Cy5-GCT-5')	113.2°	99.2°	8.4°	6.9°	7.6°
**-6GC** (3'-TAC-Cy5-GCT-5')	126.0°	97.4°	8.1°	3.9°	5.6°
**-7GC** (3'-TAC-Cy5-GCT-5')	83.7°	88.9°	16.1°	4.7°	8.7°
**-8GC** (3'-TAC-Cy5-GCT-5')	67.6°	86.6°	11.6°	6.3°	8.6°
**-9GC** (3'-TAC-Cy5-GCT-5')	28.1°	89.3°	5.8°	3.1°	4.3°
**-10GC** (3'-TAC-Cy5-GCT-5')	82.8°	23.0°	13.1°	6.8°	9.4°
**6GC/0** (=**6GC**)	14.5°	73.6°	14.9°	9.8°	12.1°
**6GC/1**	76.0°	93.1°	10.0°	6.8°	8.3°
**6GC/2**	59.9°	86.5°	15.6°	7.9°	11.1°
**6GC/3**	89.3°	100.0°	17.0°	13.9°	15.4°
**6GC/4**	65.2°	104.7°	21.6°	12.1°	16.2°
**6GC/5**	47.2°	111.5°	8.8°	5.2°	6.8°
**6GC/6**	89.3°	51.6°	13.9°	6.2°	9.3°
**6GC/7**	69.0°	15.2°	18.9°	11.8°	14.9°
**6GC/8**	80.8°	10.0°	11.9°	8.9°	10.3°

Table S2: Fitting parameters for the linear fittings in Figure 2 of the Main Text.

Sample	Intercept	Slope	Pearson's r	R-square (COD)	Adj. R-Square
**bTT**	292.6187 ± 22.5619	-35.0495 ± 2.9332	-0.98628	0.97275	0.96594
**bAA**	217.9975 ± 18.3673	-31.6574 ± 2.3879	-0.98881	0.97775	0.97219

Table S3: DFT results of optimized structures shown in Figure S4. Distances and angles were calculated based on the structural definitions in S5. The interaction energy in kcal/mol was calculated as $E_{int} = E_{system} - E_{base} - E_{dye}$, where E was the energy of the fully relaxed molecule. Angles and distances were measured as in Figure S5.

DNA base (structure)	Interaction energy (kcal/mol)	Dye-base distance (nm)	Stack angle (°)	Planar angle (°)	Orientation
A (A1)	−14.93	0.37	96.46	3.5	Parallel stacking
A (A2)	−13.66	0.36	107.26	10.44	Parallel stacking
A (A3)	−13.49	0.38	117.36	8.56	Parallel stacking
T (T1)	−13.05	0.37	111.05	10.57	Parallel stacking
T (T2)	−12.13	0.36	91.65	16.81	Parallel stacking
T (T3)	−11.98	0.38	114.31	11.07	Parallel stacking
T (T4)	−10.57	0.38	114.31	11.07	Parallel stacking
C (C1)	−14.09	0.35	99.05	4.18	Parallel stacking
C (C2)	−16.60	0.49	76.53	81.17	T stacking
C (C3)	−19.31	0.48	79.22	88.78	T stacking
G (G1)	−18.37	0.42	114.07	7.67	Parallel stacking
G (G2)	−17.09	0.38	112.44	6.13	Parallel stacking
G (G3)	−18.41	0.46	67.44	49.65	Oblique stacking
G (G4)	−19.08	0.64	32.38	36.59	Stacking with the Cy5 axis

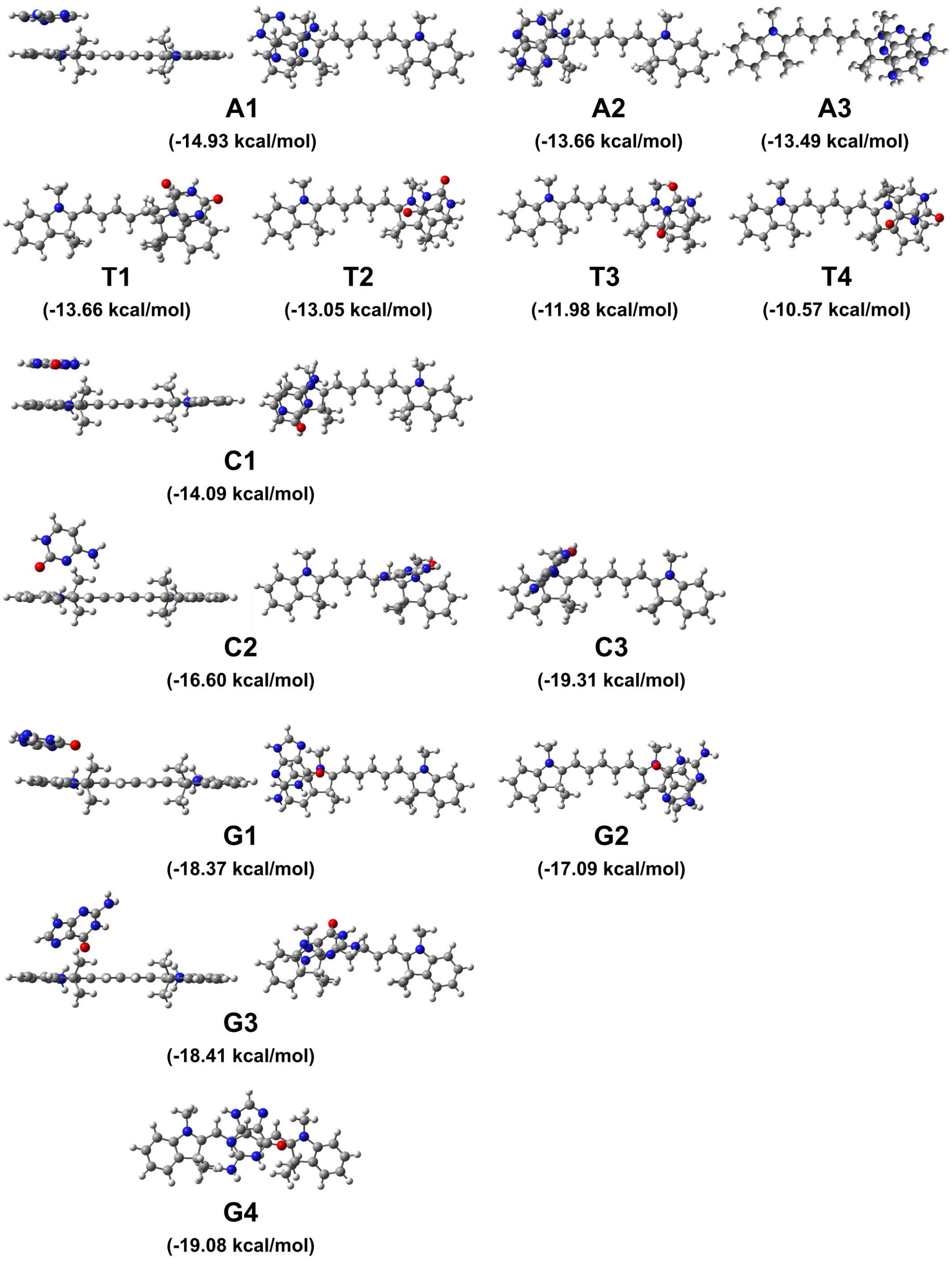

Figure S4: Molecular structures of the interaction between Cy5 and the DNA bases optimized using density functional theory (DFT). These structures represent all the different 32 optimized structures. See Table S3 for structural parameters related to stacking.

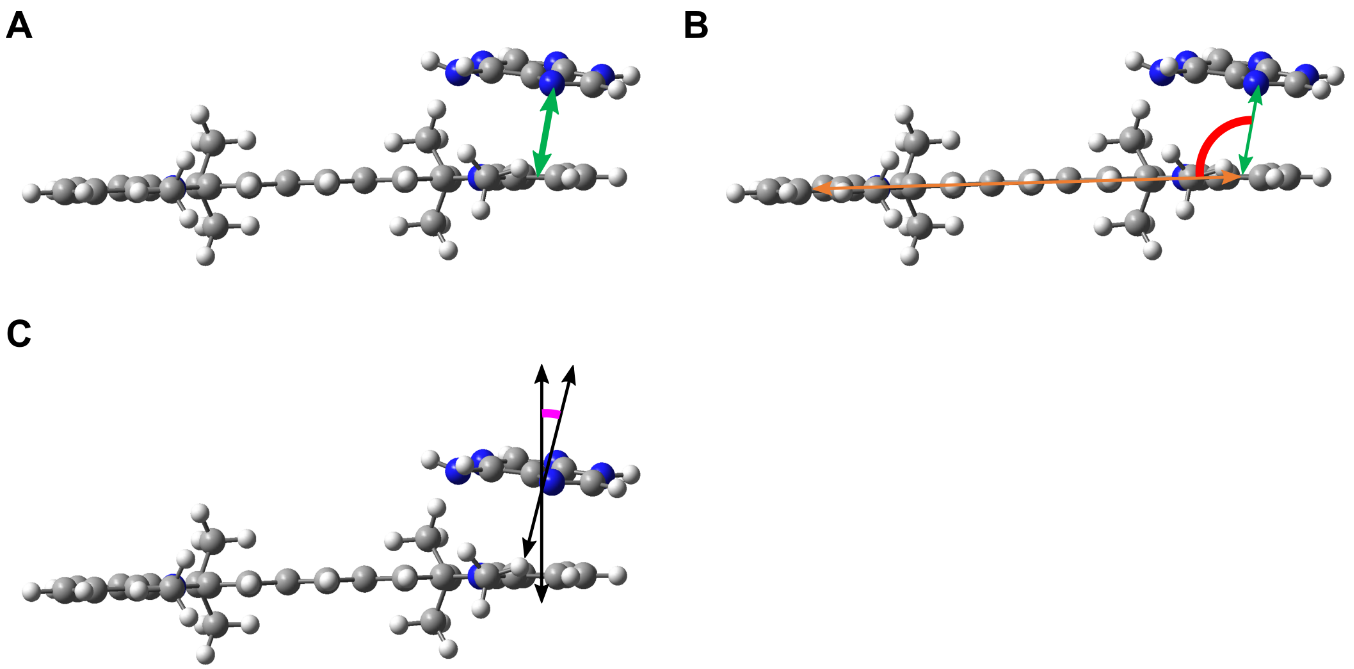

Figure S5: Definition of distances and angles for the interaction between the indole of Cy5 and the DNA bases. A) The dye-base distance was defined as the norm of the vector (green arrow) from the indole center to the base center. B) The stack angle between the indole and the base was defined as the angle angle (shown in red) between the long dye axis and the dye-base vector. C) The planar angle was defined as the angle (shown in pink) between the normal planes of the indole and the base.

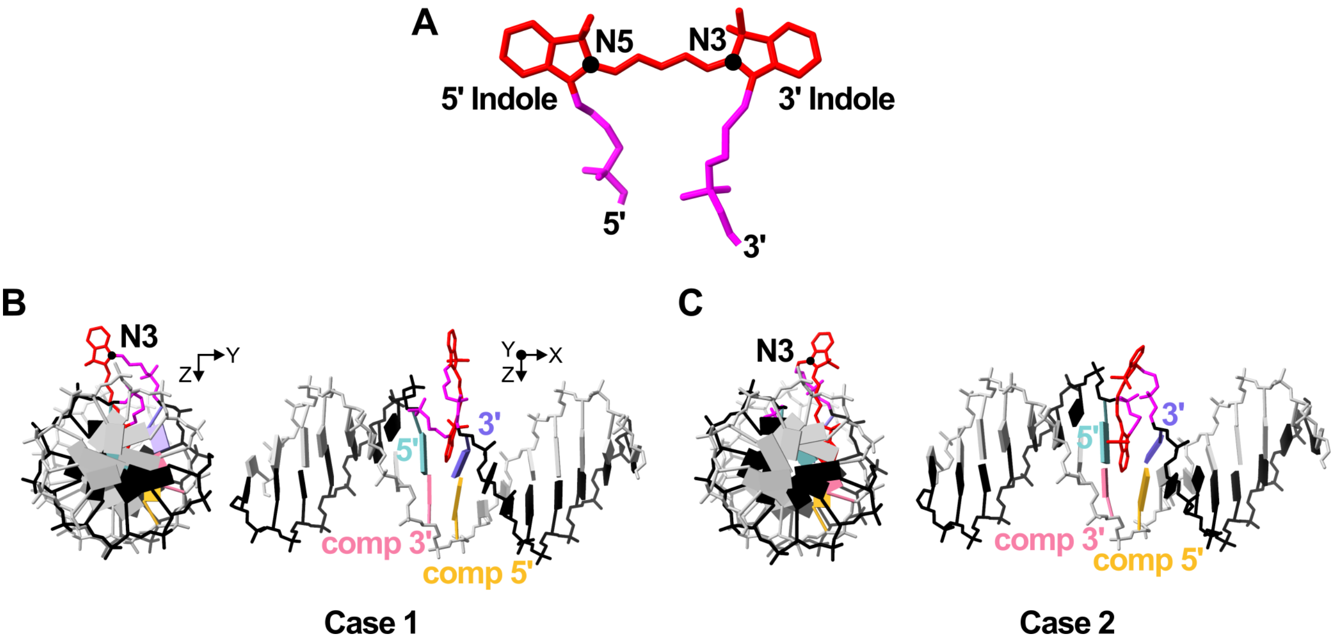

Figure S6: Initialization cases for MD simulations. A) Cy5 and the two tethers. 5' and 3' end indoles are labeled as 5' and 3' Indole. 5' and 3' end nitrogen atoms are labeled as N5 and N3. B) Initialization with 5' indole intercalated. Nitrogens are facing towards the front. C) Initialization with 5' indole intercalated. Nitrogens are facing towards the back. The 5' and 3' end flanking bases are labeled as 5' and 3' bases. The complementary 5' and 3' end flanking bases are labeled as complementary 5' and complementary 3' bases.

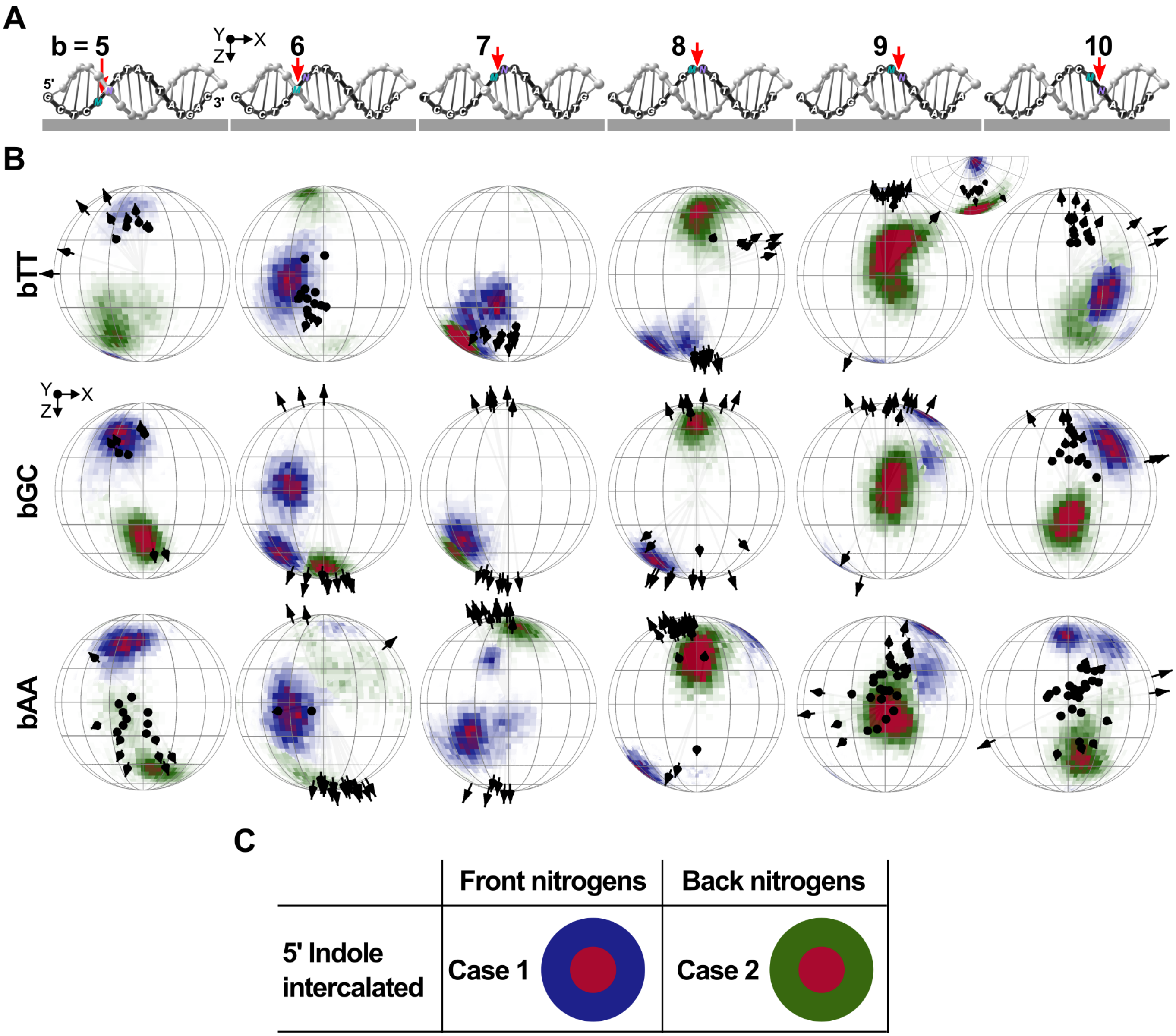

Figure S7: Simulation results when the 5' end of the Cy5 indole was intercalated.

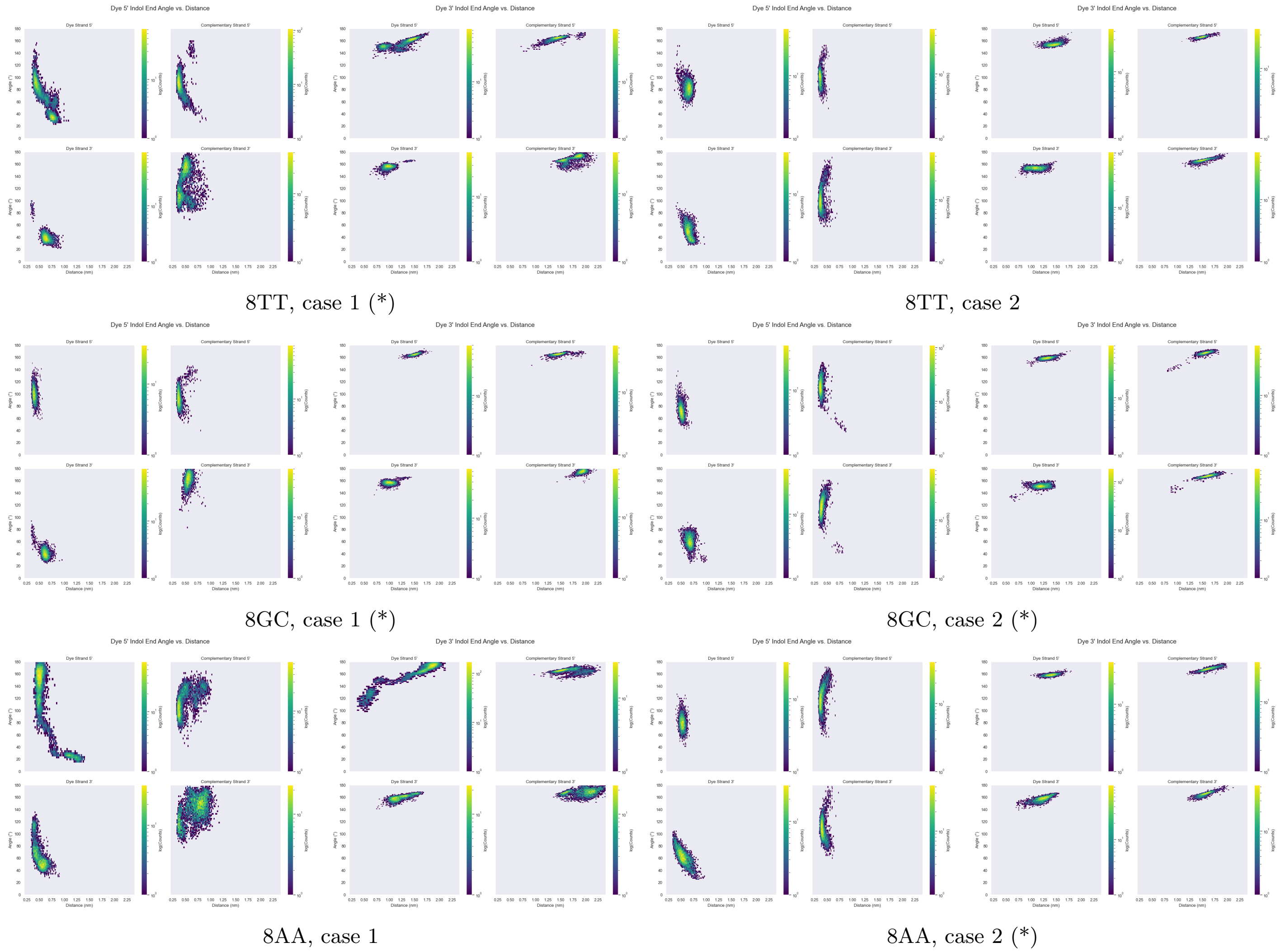

Figure S8: Interaction of the neighboring bases with the 5' and 3' end indoles of Cy5. The nomenclature conversion between these plots and the schematics in Figure S6 is as follows. Dye Strand 5' is 5' base, Dye Strand 3' is 3' base, Complementary Strand 5' is comp 5', and Complementary Strand 3' is comp 3'. The measurements in these plots are given as in the schematics in Figure S5 as follows. Angle is the stack angle, and distance is the indole center to base center distance. (*) indicates the cases that agree with the experimental data.

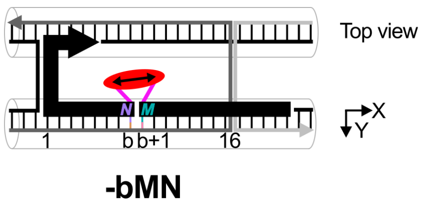

Figure S9: The design of the template for investigating the effect of the surface.

Table S4: DNA strands used for making the different DNA origami samples. As stated in the Main Text, the DNA origami was designed based on [5]. The staple strands for the DNA origami are divided in edges, body and docking sites. Staples for the DNA origami edges have polythymine sequences for passivation. The sequence of the M13mp18 scaffold can be obtained from www.bayoubiolabs.com/productsheets/M13mp18_DNA_Sequence_BayouBiolabs.pdf.

Name	Sequence	Role
Ptt-1LR2-1LR1-Ptt	TTTTCACGTTGAAAATCTCGCGAATAATAATTTTTTTT	DNA origami edges
Ptt-1LR4-1LR3-Ptt	TTTAGGAAGTTTCCATTAATAAAGACTTTTTCATGTTT	DNA origami edges
Ptt-1LR6-1LR5-Ptt	TTTCAGGCGCATAGGCTGGTGAACGGTGTACAGACTTT	DNA origami edges
Ptt-1LR8-1LR7-Ptt	TTTGGTAGAAAGATTCATCGAACAACATTATTACATTT	DNA origami edges
Ptt-1LR10-1LR9-Ptt	TTTTGACCATAAATCAAAAGTTCAGAAAACGAGAATTT	DNA origami edges
Ptt-1LR12-1LR11-Ptt	TTTGTGTCTGGAAGTTTCAATGCAACTAAAGTACGTTT	DNA origami edges
Ptt-1LR14-1LR13-Ptt	TTTTTTTGCGGGAGAAGCCTATGACCCTGTAATACTTT	DNA origami edges
Ptt-1LR16-1LR15-Ptt	TTTGTCAATCATATGTACCATCGTAAAACTAGCATTTT	DNA origami edges
Ptt-1LR18-1LR17-Ptt	TTTGTGTAGATGGGCGCATGGGATAGGTCACGTTGTTT	DNA origami edges
Ptt-1LR20-1LR19-Ptt	TTTAGTGCCAAGCTTGCATTTGTAAAACGACGGCCTTT	DNA origami edges
Ptt-1LR22-1LR21-Ptt	TTTTATTGGGCGCCAGGGTGGAGAGGCGGTTTGCGTTT	DNA origami edges
Ptt-1LR24-1LR23-Ptt	TTTTGGCCCACTACGTGAACCGTCTATCAGGGCGATTT	DNA origami edges
Ptt-RR1-RR2-Ptt	TTTCAGAACCGCCACCCTCTCAGAACCGCCACCCTTTT	DNA origami edges
Ptt-RR3-RR4-Ptt	TTTATACAGGAGTGTACTGTACATGGCTTTTGATGTTT	DNA origami edges
Ptt-RR5-RR6-Ptt	TTTCGTTTGCCATCTTTTCATAGCCCCCTTATTAGTTT	DNA origami edges
Ptt-RR7-RR8-Ptt	TTTCAAAGACAAAAGGGCGTATGGTTTACCAGCGCTTT	DNA origami edges
Ptt-RR9-RR10-Ptt	TTTAGAGCAAGAAACAATGGTTAAGCCCAATAATATTT	DNA origami edges
Ptt-RR11-RR12-Ptt	TTTCAATTTTATCCTGAATATTTTGCACCCAGCTATTT	DNA origami edges
Ptt-RR13-RR14-Ptt	TTTTATCCCATCCTAATTTTGAACAAGAAAAATAATTT	DNA origami edges
Ptt-RR15-RR16-Ptt	TTTCATAATTACTAGAAAAGAATAAACACCGGAATTTT	DNA origami edges
Ptt-RR17-RR18-Ptt	TTTAATCCTTGAAAACATAATTAATTTTCCCTTAGTTT	DNA origami edges
Ptt-RR19-RR20-Ptt	TTTAGATGAATATACAGTATTTCAGGTTTAACGTCTTT	DNA origami edges
Ptt-RR21-RR22-Ptt	TTTAGACTTTACAAACAATAGGATTTAGAAGTATTTTT	DNA origami edges
Ptt-RR23-RR24-Ptt	TTTAAAAATACCGAACGAACTAAAACATCGCCATTTTT	DNA origami edges
A2-A4	AGGCTCCAGAGGCTTTGAGGACACGGGTAA	DNA origami body
A4-A6	AATACGTTTGAAAGAGGACAGACTGACCTT	DNA origami body
A6-A8	CATCAAGTAAAACGAACTAACGAGTTGAGA	DNA origami body
A8-A10	TTTAGGACAAATGCTTTAAACAATCAGGTC	DNA origami body
A10-A12	TTTACCCCAACATGTTTTAAATTTCCATAT	DNA origami body
A12-A14	AACAGTTTTGTACCAAAAACATTTTATTTC	DNA origami body
A14-A16	AACGCAAAATCGATGAACGGTACCGGTTGA	DNA origami body
A16-A18	TAATCAGCGGATTGACCGTAATCGTAACCG	DNA origami body
A18-A20	TGCATCTTTCCCAGTCACGACGGCCTGCAG	DNA origami body
A20-A22	GTCGACTTCGGCCAACGCGCGGGGTTTTTC	DNA origami body
A22-A24	TTTTCACTCAAAGGGCGAAAAACCATCACC	DNA origami body
A24-B23	CAAATCAAGTTTTTTGGGGTCGAAACGTGGA	DNA origami body
B1-A2	AGAAAGGAACAACTAAAGGAATTCAAAAAAA	DNA origami body
B3-B1	ACGGCTACAAAAGGAGCCTTTAATGTGAGAAT	DNA origami body
B5-B3	GACCAACTAATGCCACTACGAAGGGGGTAGCA	DNA origami body
B7-B5	TACGTTAAAGTAATCTTGACAAGAACCGAACT	DNA origami body
B9-B7	ATCCCCCTATACCACATTCAACTAGAAAAATC	DNA origami body
B11-B9	CTGTAGCTTGACTATTATAGTCAGTTCATTGA	DNA origami body
B13-B11	TAAATCGGGATTCCCAATTCTGCGATATAATG	DNA origami body
B15-B13	AACAAGAGGGATAAAAATTTTTAGCATAAAGC	DNA origami body
B17-B15	ACAAACGGAAAAGCCCCAAAAACACTGGAGCA	DNA origami body
B19-B17	CCAGGGTTGCCAGTTTGAGGGGACCCGTGGGA	DNA origami body
B21-B19	TTAATGAACTAGAGGATCCCCGGGGGGTAACG	DNA origami body
B23-B21	CTCCAACGCAGTGAGACGGGCAACCAGCTGCA	DNA origami body
C2-C4	TTTATCAGGACAGCATCGGAACGACACCAACC	DNA origami body
C6-C8	TTCATTACGTCAGGACGTTGGGAAATGCAGAT	DNA origami body
C8-C10	ACATAACGGGAATCGTCATAAATAAAGCAAAG	DNA origami body
C10-C12	CGGATTGCAGAGCTTAATTGCTGAAACGAGTA	DNA origami body
C12-C14	GATTTAGTCAATAAAGCCTCAGAGAACCCTCA	DNA origami body
C14-C16	TATATTTTGTCATTGCCTGAGAGTGGAAGATT	DNA origami body
C16-C18	GTATAAGCCAACCCGTCGGATTCTGACGACAG	DNA origami body
C18-C20	TATCGGCCGCAAGGCGATTAAGTTTACCGAGC	DNA origami body
C20-C22	TCGAATTCGGGAAACCTGTCGTGCAGCTGATT	DNA origami body
C22-C24	GCCCTTCAGAGTCCACTATTAAAGGGTGCCGT	DNA origami body

Continued on next page

Name	Sequence	Role
C24-D23	AAAGCACTAAATCGGAACCCTAATCCAGTT	DNA origami body
D1-C2	ACAACTTTCAACAGTTTCAGCGGATGTATCGG	DNA origami body
D3-D1	CAGCGAAACTTGCTTTCGAGGTGTTGCTAA	DNA origami body
D5-D3	GCGCAGACAAGAGGCAAAAGAATCCCTCAG	DNA origami body
D7-D5	TTATACCACCAAATCAACGTAACGAACGAG	DNA origami body
D9-D7	AATACTGCCCAAAAGGAATTACGTGGCTCA	DNA origami body
D11-D9	GATGGCTTATCAAAAAGATTAAGAGCGTCC	DNA origami body
D13-D11	AAATTAAGTTGACCATTAGATACTTTTGCG	DNA origami body
D15-D13	GCTATCAGAAATGCAATGCCTGAATTAGCA	DNA origami body
D17-D15	GCGAGTAAAAATATTTAAATTGTTACAAAG	DNA origami body
D19-D17	GATGTGCTTCAGGAAGATCGCACAATGTGA	DNA origami body
D21-D19	TTCCAGTCGTAATCATGGTCATAAAAGGGG	DNA origami body
D23-D21	TGGAACAACCGCCTGGCCCTGAGGCCCGCT	DNA origami body
E2-E4	AAACAGCTTTTTGCGGGATCGTCAACACTAAA	DNA origami body
E4-E6	ACACTCATCCATGTTACTTAGCCGAAAGCTGC	DNA origami body
E6-E8	TCATTCAGATGCGATTTTAAGAACAGGCATAG	DNA origami body
E8-E10	TAAGAGCAAATGTTTAGACTGGATAGGAAGCC	DNA origami body
E10-E12	CGAAAGACTTTGATAAGAGGTCATATTTCGCA	DNA origami body
E12-E14	AATGGTCAACAGGCAAGGCAAAGAGTAATGTG	DNA origami body
E14-E16	TAGGTAAACTATTTTTGAGAGATCAAACGTTA	DNA origami body
E16-E18	ATATTTTGGCTTTCATCAACATTATCCAGCCA	DNA origami body
E18-E20	GCTTTCCGATTACGCCAGCTGGCGGCTGTTTC	DNA origami body
E20-E22	CTGTGTGATTGCGTTGCGCTCACTAGAGTTGC	DNA origami body
E22-E24	AGCAAGCGTAGGGTTGAGTGTTGTAGGGAGCC	DNA origami body
F1-E2	TAAATGAATTTTCTGTATGGGATTAATTTCTT	DNA origami body
F3-F1	AAGGCCGCTGATACCGATAGTTGCGACGTTAG	DNA origami body
F5-F3	GACCTGCTCTTTGACCCCCAGCGAGGGAGTTA	DNA origami body
F7-F5	ATTACCTTTGAATAAGGCTTGCCCAAATCCGC	DNA origami body
F9-F7	AATAGTAAACACTATCATAACCCTCATTGTGA	DNA origami body
F11-F9	TTGCTCCTTTCAAATATCGCGTTTGAGGGGGT	DNA origami body
F13-F11	TAAATCATATAACCTGTTTAGCTAACCTTTAA	DNA origami body
F15-F13	GAGGGTAGGATTCAAAAGGGTGAGACATCCAA	DNA origami body
F17-F15	TGTAGCCATTAAAATTCGCATTAAATGCCGGA	DNA origami body
F19-F17	TCTTCGCTGCACCGCTTCTGGTGCGGCCTTCC	DNA origami body
F21-F19	CACATTAAAATTGTTATCCGCTCATGCGGGCC	DNA origami body
F23-F21	GCCCGAGAGTCCACGCTGGTTTGCAGCTAACT	DNA origami body
E24-F23	CCCGATTTAGAGCTTGACGGGGAAAAAGAATA	DNA origami body
G2-G4	TGACAACTCGCTGAGGCTTGCATTATACCA	DNA origami body
G4-G6	AGCGCGATGATAAATTGTGTCGTGACGAGA	DNA origami body
G6-G8	AACACCAAATTTCAACTTTAATCGTTTACC	DNA origami body
G8-G10	AGACGACAAAGAAGTTTTGCCATAATTCGA	DNA origami body
G10-G12	GCTTCAATCAGGATTAGAGAGTTATTTTCA	DNA origami body
G12-G14	TTTGGGGATAGTAGTAGCATTAAAAGGCCG	DNA origami body
G14-G16	GAGACAGCTAGCTGATAAATTAATTTTTGT	DNA origami body
G16-G18	TAAATCAAAATAATTCGCGTCTCGGAAACC	DNA origami body
G18-G20	AGGCAAAGGGAAGGGCGATCGGCAATTCCA	DNA origami body
G20-G22	CACAACAGGTGCCTAATGAGTGCCCAGCAG	DNA origami body
G22-G24	GCGAAAAATCCCTTATAAATCAAGCCGGCG	DNA origami body
G24-I24	AACGTGGCGAGAAAGGAAGGGAAACCAGTAA	DNA origami body
H1-G2	TCTAAAGTTTTGTCGTCTTTCCAGCCGACAA	DNA origami body
H3-I2	ATATTCGGAACCATCGCCCACGCAGAGAAGGA	DNA origami body
H5-I4	TCATCGCCAACAAAGTACAACGGACGCCAGCA	DNA origami body
H7-I6	GATGGTTTGAACGAGTAGTAAATTTACCATTA	DNA origami body
H9-I8	CTTTTGCAGATAAAAACCAAAATAAAGACTCC	DNA origami body
H11-I10	CCAACAGGAGCGAACCAGACCGGAGCCTTTAC	DNA origami body
H13-I12	TTCTACTACGCGAGCTGAAAAGGTTACCGCGC	DNA origami body
H15-I14	CAACCGTTTCAAATCACCATCAATTCGAGCCA	DNA origami body
H17-I16	GCCATCAAGCTCATTTTTTAACCACAAATCCA	DNA origami body
H19-I18	CAACTGTTGCGCCATTCGCCATTCAAACATCA	DNA origami body
H21-I20	AAGCCTGGTACGAGCCGGAAGCATAGATGATG	DNA origami body
H23-I22	TCGGCAAATCCTGTTTGATGGTGGACCCTCAA	DNA origami body
I2-H3	TTAGGATTGGCTGAGACTCCTCAATAACCGAT	DNA origami body
I4-H5	TTGACAGGCCACCACCAGAGCCGCGATTTGTA	DNA origami body

Continued on next page

Name	Sequence	Role
I6-H7	GCAAGGCCTCACCAGTAGCACCATGGGCTTGA	DNA origami body
I8-H9	TTATTACGAAGAACTGGCATGATTGCGAGAGG	DNA origami body
I10-H11	AGAGAGAAAAAAATGAAAATAGCAAGCAAACT	DNA origami body
I12-H13	CCAATAGCTCATCGTAGGAATCATGGCATCAA	DNA origami body
I14-H15	GTAATAAGTTAGGCAGAGGCATTTATGATATT	DNA origami body
I16-H17	ATCGCAAGTATGTAAATGCTGATGATAGGAAC	DNA origami body
I18-H19	AGAAAACAAAGAAGATGATGAAACAGGCTGCG	DNA origami body
I20-H21	GCAATTCACATATTCCTGATTATCAAAGTGTA	DNA origami body
I22-H23	TCAATATCGAACCTCAAATATCAATTCCGAAA	DNA origami body
I24-J23	TAAAAGGGACATTCTGGCCAACAAAGCATC	DNA origami body
J1-H1	TCCACAGACAGCCCTCATAGTTAGCGTAACGA	DNA origami body
J3-J1	TATTAAGAAGCGGGGTTTTGCTCGTAGCAT	DNA origami body
J5-J3	CACCAGAAAGGTTGAGGCAGGTCATGAAAG	DNA origami body
J7-J5	CAGCAAAAGGAAACGTCACCAATGAGCCGC	DNA origami body
J9-J7	ATACCCAACAGTATGTTAGCAAATTAGAGC	DNA origami body
J11-J9	TTAACGTCTAACATAAAAACAGGTAACGGA	DNA origami body
J13-J11	TTTTATTTAAGCAAATCAGATATTTTTTGT	DNA origami body
J15-J13	CATGTAATAGAATATAAAGTACCAAGCCGT	DNA origami body
J17-J15	TATAACTAACAAAGAACGCGAGAACGCCAA	DNA origami body
J19-J17	CTGAGCAAAAATTAATTACATTTTGGGTTA	DNA origami body
J21-J19	ATTATCATTCAATATAATCCTGACAATTAC	DNA origami body
J23-J21	ACCTTGCTTGGTCAGTTGGCAAAGAGCGGA	DNA origami body
K2-K4	GCGGATAACCTATTATTCTGAAACAGACGATT	DNA origami body
K4-K6	GGCCTTGAAGAGCCACCACCCTCAGAAACCAT	DNA origami body
K6-K8	CGATAGCATTGAGCCATTTGGGAACGTAGAAA	DNA origami body
K8-K10	ATACATACCGAGGAAACGCAATAAGAAGCGCA	DNA origami body
K10-K12	TTAGACGGCCAAATAAGAAACGATAGAAGGCT	DNA origami body
K12-K14	TATCCGGTCTCATCGAGAACAAGCGACAAAAG	DNA origami body
K14-K16	GTAAAGTAATCGCCATATTTAACAAAACTTTT	DNA origami body
K16-K18	TCAAATATAACCTCCGGCTTAGGTAACAATTT	DNA origami body
K18-K20	CATTTGAAGGCGAATTATTCATTTTTGTTTGG	DNA origami body
K20-K22	ATTATACTAAGAAACCACCAGAAGTCAACAGT	DNA origami body
K22-K24	TGAAAGGAGCAAATGAAAAATCTAGAGATAGA	DNA origami body
K24-L23	ACCCTTCTGACCTGAAAGCGTAAGACGCTGAG	DNA origami body
L1-K2	TCACCAGTACAAACTACAACGCCTAGTACCAG	DNA origami body
L3-L1	TTTCGGAAGTGCCGTCGAGAGGGTGAGTTTCG	DNA origami body
L5-L3	CCACCCTCTATTCACAAACAAATACCTGCCTA	DNA origami body
L7-L5	TCACCGACGCACCGTAATCAGTAGCAGAACCG	DNA origami body
L9-L7	AAGGAAACATAAAGGTGGCAACATTATCACCG	DNA origami body
L11-L9	ATCCCAATGAGAATTAACTGAACAGTTACCAG	DNA origami body
L13-L11	GTACCGCAATTCTAAGAACGCGAGTATTATTT	DNA origami body
L15-L13	AATTGAGAATTCTGTCCAGACGACTAAACCAA	DNA origami body
L17-L15	ACCTTTTTATTTTAGTTAATTTCATAGGGCTT	DNA origami body
L19-L17	CGCGCAGATTACCTTTTTTAATGGGAGAGACT	DNA origami body
L21-L19	GCGGAACATCTGAATAATGGAAGGTACAAAAT	DNA origami body
L23-L21	AGCCAGCAATTGAGGAAGGTTATCATCATTTT	DNA origami body
M2-M4	GTATAGCAAACAGTTAATGCCCAATCCTCA	DNA origami body
M4-M6	TTAAAGCCAGAGCCGCCACCCTCGACAGAA	DNA origami body
M6-M8	TCAAGTTTCATTAAAGGTGAATATAAAAGA	DNA origami body
M8-M10	AACGCAAAGATAGCCGAACAAACCCTGAAC	DNA origami body
M10-M12	AAAGTCACAAAATAAACAGCCAGCGTTTTA	DNA origami body
M12-M14	GCGAACCTCCAAGAACGGGTATGACAATAA	DNA origami body
M14-M16	ACAACATGCCAACGCTCAACAGTCTTCTGA	DNA origami body
M16-M18	CCTAAATCAAAATCATAGGTCTAAACAGTA	DNA origami body
M18-M20	CATAAATCTTTGAATACCAAGTGTTAGAAC	DNA origami body
M20-M22	CTACCATAGTTTGAGTAACATTTAAAATAT	DNA origami body
M22-M24	CTTTAGGGCCTGCAACAGTGCCAATACGTG	DNA origami body
N1-M2	AGGAACCCATGTACCGTAACACTTGATATAA	DNA origami body
N3-N1	GCCCGTATCCGGAATAGGTGTATCAGCCCAAT	DNA origami body
N5-N3	GCCTCCCTCAGAATGGAAAGCGCAGTAACAGT	DNA origami body
N7-N5	GAAATTATTGCCTTTAGCGTCAGACCGGAACC	DNA origami body
N9-N7	AAGTAAGCAGACACCACGGAATAATATTGACG	DNA origami body
N11-N9	GCCAGTTAGAGGGTAATTGAGCGCTTTAAGAA	DNA origami body

Continued on next page

Name	Sequence	Role
N13-N11	CTTATCATTCCCGACTTGCGGGAGCCTAATTT	DNA origami body
N15-N13	AGTATAAAGTTCAGCTAATGCAGATGTCTTTC	DNA origami body
N17-N15	GAATTTATTTAATGGTTTGAAATATTCTTACC	DNA origami body
N19-N17	CCTGATTGCAATATATGTGAGTGATCAATAGT	DNA origami body
N21-N19	ATTTTAAAATCAAAATTATTTGCACGGATTCG	DNA origami body
N23-N21	TTAACACCAGCACTAACAACTAATCGTTATTA	DNA origami body
M24-N23	GCACAGACAATATTTTTGAATGGGGTCAGTA	DNA origami body
O2-O4	CAGGAGGTGGGGTCAGTGCCTTGAGTCTCTGA	DNA origami body
O4-O6	ATTTACCGGGAACCAGAGCCACCACTGTAGCG	DNA origami body
O6-O8	CGTTTTCAAGGGAGGGAAGGTAAAGTTTATTT	DNA origami body
O8-O10	TGTCACAATCTTACCGAAGCCCTTTAATATCA	DNA origami body
O10-O12	GAGAGATAGAGCGTCTTTCCAGAGGTTTTGAA	DNA origami body
O12-O14	GCCTTAAACCAATCAATAATCGGCACGCGCCT	DNA origami body
O14-O16	GTTTATCAATATGCGTTATACAAACCGACCGT	DNA origami body
O16-O18	GTGATAAAAAGACGCTGAGAAGAGATAACCTT	DNA origami body
O18-O20	GCTTCTGTTCGGGAGAAACAATAACGTAAAAC	DNA origami body
O20-O22	AGAAATAAAAATCCTTTGCCCGAAAGATTAGA	DNA origami body
O22-O24	GCCGTCAAAAAACAGAGGTGAGGCCTATTAGT	DNA origami body
O24-P23	CTTTAATGCGCGAACTGATAGCCCCACCAG	DNA origami body
P1-O2	CCACCCTCATTTTCAGGGATAGCAACCGTACT	DNA origami body
P3-P1	GTTTTAACTTAGTACCGCCACCCAGAGCCA	DNA origami body
P5-P3	AAATCACCTTCCAGTAAGCGTCAGTAATAA	DNA origami body
P7-P5	ACCGATTGTCGGCATTTTCGGTCATAATCA	DNA origami body
P9-P7	AATAGCTATCAATAGAAAATTCAACATTCA	DNA origami body
P11-P9	ACGCTAACACCCACAAGAATTGAAAATAGC	DNA origami body
P13-P11	TGTAGAAATCAAGATTAGTTGCTCTTACCA	DNA origami body
P15-P13	TTAGTATCACAATAGATAAGTCCACGAGCA	DNA origami body
P17-P15	CTTAGATTTAAGGCGTTAAATAAAGCCTGT	DNA origami body
P19-P17	CTTTTACAAAATCGTCGCTATTAGCGATAG	DNA origami body
P21-P19	CTCGTATTAGAAATTGCGTAGATACAGTAC	DNA origami body
P23-P21	CAGAAGATTAGATAATACATTTGTCGACAA	DNA origami body
A2-A4-zPS3	AGGCTCCAGAGGCTTTGAGGACACGGGTAATTGGGAGGA	DNA origami docking sites
A10-A12-zPS3	TTTACCCCAACATGTTTTAAATTTCCATATTTGGGAGGA	DNA origami docking sites
A18-A20-zPS3	TGCATCTTTCCCAGTCACGACGGCCTGCAGTTGGGAGGA	DNA origami docking sites
G2-G4-zPS3	TGACAACTCGCTGAGGCTTGCATTATACCATTGGGAGGA	DNA origami docking sites
K2-K4-zPS3	GCGGATAACCTATTATTCTGAAACAGACGATTTTGGGAGGA	DNA origami docking sites
M6-M8-zPS3	TCAAGTTTCATTAAAGGTGAATATAAAAGATTGGGAGGA	DNA origami docking sites
M14-M16-zPS3	ACAACATGCCAACGCTCAACAGTCTTCTGATTGGGAGGA	DNA origami docking sites
O2-O4-zPS3	CAGGAGGTGGGGTCAGTGCCTTGAGTCTCTGATTGGGAGGA	DNA origami docking sites
O10-O12-zPS3	GAGAGATAGAGCGTCTTTCCAGAGGTTTTGAATTGGGAGGA	DNA origami docking sites
O18-O20-zPS3	GCTTCTGTTCGGGAGAAACAATAACGTAAAACTTGGGAGGA	DNA origami docking sites
PS3	TCCTCCC/Cy3b/	DNA-PAINT imager strand
bAA-Cy5	ATATAATCGCTCA/Cy5/AATATTATGACTG	Cy5-tethered strand
bGC-Cy5	ATATAATCGCTCG/Cy5/CATATTATGACTG	Cy5-tethered strand
bTT-Cy5	ATATAATCGCTCT/Cy5/TATATTATGACTG	Cy5-tethered strand
5AA-seam	CCATCAATGATTATATGTAATAAGGTCATAATATTTGAGCATG ATATTCAACCGTTTCAAATCA	Replaces H13-I12 and I12-H13
5AA-staple	TATAACTAACAAAGAACGCGAGAACGCCAACATGTACAAGAAT ATAAAGTACCAAGCCGT	Replaces J15-J13 and J13-J11
6AA-seam	CCATCAATATTATATAGTAATAAGTCATAATATTTGAGCGATG ATATTCAACCGTTTCAAATCA	Replaces H13-I12 and I12-H13
6AA-staple	TATAACTAACAAAGAACGCGAGAACGCCAACATGTCAGAGAAT ATAAAGTACCAAGCCGT	Replaces J15-J13 and J13-J11
7AA-seam	CCATCAATTTATATCAGTAATAAGCATAATATTTGAGCGAATG ATATTCAACCGTTTCAAATCA	Replaces H13-I12 and I12-H13
7AA-staple	TATAACTAACAAAGAACGCGAGAACGCCAACATGCAGTAGAAT ATAAAGTACCAAGCCGT	Replaces J15-J13 and J13-J11
8AA-seam	CCATCAATTATATCCAGTAATAAGATAATATTTGAGCGATATG ATATTCAACCGTTTCAAATCA	Replaces H13-I12 and I12-H13
8AA-staple	TATAACTAACAAAGAACGCGAGAACGCCAACATCAGTCAGAAT ATAAAGTACCAAGCCGT	Replaces J15-J13 and J13-J11
9AA-seam	CCATCAATATATGCCAGTAATAAGTAATATTTGAGCGATTATG ATATTCAACCGTTTCAAATCA	Replaces H13-I12 and I12-H13

Continued on next page

Table S4 – *Continued from previous page*

Name	Sequence	Role
9AA-staple	TATAACTAACAAAGAACGCGAGAACGCCAACACAGTCAAGAAT ATAAAGTACCAAGCCGT	Replaces J15-J13 and J13-J11
10AA-seam	CCATCAATTATAGCCAGTAATAAGAATATTTGAGCGATTAATG ATATTCAACCGTTTCAAATCA	Replaces H13-I12 and I12-H13
10AA-staple	TATAACTAACAAAGAACGCGAGAACGCCAACCAGTCATAGAAT ATAAAGTACCAAGCCGT	Replaces J15-J13 and J13-J11
5GC-seam	CCATCAATGATTATATGTAATAAGGTCATAATATGCGAGCATG ATATTCAACCGTTTCAAATCA	Replaces H13-I12 and I12-H13
5GC-staple	TATAACTAACAAAGAACGCGAGAACGCCAACATGTACAAGAAT ATAAAGTACCAAGCCGT	Replaces J15-J13 and J13-J11
6GC-seam	CCATCAATATTATATAGTAATAAGTCATAATATGCGAGCGATG ATATTCAACCGTTTCAAATCA	Replaces H13-I12 and I12-H13
6GC-staple	TATAACTAACAAAGAACGCGAGAACGCCAACATGTCAGAGAAT ATAAAGTACCAAGCCGT	Replaces J15-J13 and J13-J11
7GC-seam	CCATCAATTTATATCAGTAATAAGCATAATATGCGAGCGAATG ATATTCAACCGTTTCAAATCA	Replaces H13-I12 and I12-H13
7GC-staple	TATAACTAACAAAGAACGCGAGAACGCCAACATGCAGTAGAAT ATAAAGTACCAAGCCGT	Replaces J15-J13 and J13-J11
8GC-seam	CCATCAATTATATCCAGTAATAAGATAATATGCGAGCGATATG ATATTCAACCGTTTCAAATCA	Replaces H13-I12 and I12-H13
8GC-staple	TATAACTAACAAAGAACGCGAGAACGCCAACATCAGTCAGAAT ATAAAGTACCAAGCCGT	Replaces J15-J13 and J13-J11
9GC-seam	CCATCAATATATGCCAGTAATAAGTAATATGCGAGCGATTATG ATATTCAACCGTTTCAAATCA	Replaces H13-I12 and I12-H13
9GC-staple	TATAACTAACAAAGAACGCGAGAACGCCAACACAGTCAAGAAT ATAAAGTACCAAGCCGT	Replaces J15-J13 and J13-J11
10GC-seam	CCATCAATTATAGCCAGTAATAAGAATATGCGAGCGATTAATG ATATTCAACCGTTTCAAATCA	Replaces H13-I12 and I12-H13
10GC-staple	TATAACTAACAAAGAACGCGAGAACGCCAACCAGTCATAGAAT ATAAAGTACCAAGCCGT	Replaces J15-J13 and J13-J11
5TT-seam	CCATCAATGATTATATGTAATAAGGTCATAATATAAGAGCATG ATATTCAACCGTTTCAAATCA	Replaces H13-I12 and I12-H13
5TT-staple	TATAACTAACAAAGAACGCGAGAACGCCAACATGTACAAGAAT ATAAAGTACCAAGCCGT	Replaces J15-J13 and J13-J11
6TT-seam	CCATCAATATTATATAGTAATAAGTCATAATATAAGAGCGATG ATATTCAACCGTTTCAAATCA	Replaces H13-I12 and I12-H13
6TT-staple	TATAACTAACAAAGAACGCGAGAACGCCAACATGTCAGAGAAT ATAAAGTACCAAGCCGT	Replaces J15-J13 and J13-J11
7TT-seam	CCATCAATTTATATCAGTAATAAGCATAATATAAGAGCGAATG ATATTCAACCGTTTCAAATCA	Replaces H13-I12 and I12-H13
7TT-staple	TATAACTAACAAAGAACGCGAGAACGCCAACATGCAGTAGAAT ATAAAGTACCAAGCCGT	Replaces J15-J13 and J13-J11
8TT-seam	CCATCAATTATATCCAGTAATAAGATAATATAAGAGCGATATG ATATTCAACCGTTTCAAATCA	Replaces H13-I12 and I12-H13
8TT-staple	TATAACTAACAAAGAACGCGAGAACGCCAACATCAGTCAGAAT ATAAAGTACCAAGCCGT	Replaces J15-J13 and J13-J11
9TT-seam	CCATCAATATATGCCAGTAATAAGTAATATAAGAGCGATTATG ATATTCAACCGTTTCAAATCA	Replaces H13-I12 and I12-H13
9TT-staple	TATAACTAACAAAGAACGCGAGAACGCCAACACAGTCAAGAAT ATAAAGTACCAAGCCGT	Replaces J15-J13 and J13-J11
10TT-seam	CCATCAATTATAGCCAGTAATAAGAATATAAGAGCGATTAATG ATATTCAACCGTTTCAAATCA	Replaces H13-I12 and I12-H13
10TT-staple	TATAACTAACAAAGAACGCGAGAACGCCAACCAGTCATAGAAT ATAAAGTACCAAGCCGT	Replaces J15-J13 and J13-J11
-5GC-seam	CCATCAATATATGCGAGCGATTATTTAGGCAGCAGTCATAATG ATATTCAACCGTTTCAAATCA	Replaces H13-I12 and I12-H13
-5GC-staple	TATAACTAACAAAGAACGCGAGAACGCCAACATGTAATATAAT ATAAAGTACCAAGCCGT	Replaces J15-J13 and J13-J11
-6GC-seam	CCATCAATAATATGCGAGCGATTATTAGGCAGACAGTCATATG ATATTCAACCGTTTCAAATCA	Replaces H13-I12 and I12-H13
-6GC-staple	TATAACTAACAAAGAACGCGAGAACGCCAACATGTAATTATAT ATAAAGTACCAAGCCGT	Replaces J15-J13 and J13-J11

Continued on next page

Name	Sequence	Role
-7GC-seam	CCATCAATTAATATGCGAGCGATTTTAGGCAGAGCAGTCAATG ATATTCAACCGTTTCAAATCA	Replaces H13-I12 and I12-H13
-7GC-staple	TATAACTAACAAAGAACGCGAGAACGCCAACATGTAATATATT ATAAAGTACCAAGCCGT	Replaces J15-J13 and J13-J11
-8GC-seam	CCATCAATATAATATGCGAGCGATTTAGGCAGAGGCAGTCATG ATATTCAACCGTTTCAAATCA	Replaces H13-I12 and I12-H13
-8GC-staple	TATAACTAACAAAGAACGCGAGAACGCCAACATGTAATTATAT ATAAAGTACCAAGCCGT	Replaces J15-J13 and J13-J11
-8GC-seam	CCATCAATATAATATGCGAGCGATTTAGGCAGAGGCAGTCATG ATATTCAACCGTTTCAAATCA	Replaces H13-I12 and I12-H13
-8GC-staple	TATAACTAACAAAGAACGCGAGAACGCCAACATGTAATTATAT ATAAAGTACCAAGCCGT	Replaces J15-J13 and J13-J11
-9GC-seam	CCATCAATCATAATATGCGAGCGATTAGGCAGAGGCCAGTATG ATATTCAACCGTTTCAAATCA	Replaces H13-I12 and I12-H13
-9GC-staple	TATAACTAACAAAGAACGCGAGAACGCCAACATGTAATTTATA TTAAAGTACCAAGCCGT	Replaces J15-J13 and J13-J11
-10GC-seam	CCATCAATTCATAATATGCGAGCGTTAGGCAGAGGCACAGATG ATATTCAACCGTTTCAAATCA	Replaces H13-I12 and I12-H13
-10GC-staple	TATAACTAACAAAGAACGCGAGAACGCCAACATGTAATATTAT ATAAAGTACCAAGCCGT	Replaces J15-J13 and J13-J11
-5TT-seam	CCATCAATATATAAGAGCGATTATTTAGGCAGCAGTCATAATG ATATTCAACCGTTTCAAATCA	Replaces H13-I12 and I12-H13
-5TT-staple	TATAACTAACAAAGAACGCGAGAACGCCAACATGTAATATAAT ATAAAGTACCAAGCCGT	Replaces J15-J13 and J13-J11
-6TT-seam	CCATCAATAATATAAGAGCGATTATTAGGCAGACAGTCATATG ATATTCAACCGTTTCAAATCA	Replaces H13-I12 and I12-H13
-6TT-staple	TATAACTAACAAAGAACGCGAGAACGCCAACATGTAATTATAT ATAAAGTACCAAGCCGT	Replaces J15-J13 and J13-J11
-7TT-seam	CCATCAATTAATATAAGAGCGATTTTAGGCAGAGCAGTCAATG ATATTCAACCGTTTCAAATCA	Replaces H13-I12 and I12-H13
-7TT-staple	TATAACTAACAAAGAACGCGAGAACGCCAACATGTAATATATT ATAAAGTACCAAGCCGT	Replaces J15-J13 and J13-J11
-8TT-seam	CCATCAATATAATATAAGAGCGATTTAGGCAGAGGCAGTCATG ATATTCAACCGTTTCAAATCA	Replaces H13-I12 and I12-H13
-8TT-staple	TATAACTAACAAAGAACGCGAGAACGCCAACATGTAATTATAT ATAAAGTACCAAGCCGT	Replaces J15-J13 and J13-J11
-9TT-seam	CCATCAATCATAATATAAGAGCGATTAGGCAGAGGCCAGTATG ATATTCAACCGTTTCAAATCA	Replaces H13-I12 and I12-H13
-9TT-staple	TATAACTAACAAAGAACGCGAGAACGCCAACATGTAATTTATA TTAAAGTACCAAGCCGT	Replaces J15-J13 and J13-J11
-10TT-seam	CCATCAATTCATAATATAAGAGCGTTAGGCAGAGGCACAGATG ATATTCAACCGTTTCAAATCA	Replaces H13-I12 and I12-H13
-10TT-staple	TATAACTAACAAAGAACGCGAGAACGCCAACATGTAATATTAT ATAAAGTACCAAGCCGT	Replaces J15-J13 and J13-J11
6GC/1A-seam	CCATCAATATTATATAGTAATAAGCATAATATGACGAGCGATG ATATTCAACCGTTTCAAATCA	Replaces H13-I12 and I12-H13
6GC/1A-staple	TATAACTAACAAAGAACGCGAGAACGCCAACATGCAGTAGAAT ATAAAGTACCAAGCCGT	Replaces J15-J13 and J13-J11
6GC/2A-seam	CCATCAATTTATATCAGTAATAAGATAATATGAACGAGCGATG ATATTCAACCGTTTCAAATCA	Replaces H13-I12 and I12-H13
6GC/2A-staple	TATAACTAACAAAGAACGCGAGAACGCCAACATCAGTCAGAAT ATAAAGTACCAAGCCGT	Replaces J15-J13 and J13-J11
6GC/3A-seam	CCATCAATATTATATAGTAATAAGTAATATGAAACGAGCGATG ATATTCAACCGTTTCAAATCA	Replaces H13-I12 and I12-H13
6GC/3A-staple	TATAACTAACAAAGAACGCGAGAACGCCAACACAGTCAAGAAT ATAAAGTACCAAGCCGT	Replaces J15-J13 and J13-J11
6GC/4A-seam	CCATCAATATTATATAGTAATAAGAATATGAAAACGAGCGATG ATATTCAACCGTTTCAAATCA	Replaces H13-I12 and I12-H13
6GC/4A-staple	TATAACTAACAAAGAACGCGAGAACGCCAACCAGTCATAGAAT ATAAAGTACCAAGCCGT	Replaces J15-J13 and J13-J11
6GC/5A-seam	CCATCAATATTATATAGTAATAAGATATGAAAAACGAGCGATG ATATTCAACCGTTTCAAATCA	Replaces H13-I12 and I12-H13

Continued on next page

Table S4 – *Continued from previous page*

Name	Sequence	Role
6GC/5A-staple	TATAACTAACAAAGAACGCGAGAACGCCAACAGTCATAAGAAT ATAAAGTACCAAGCCGT	Replaces J15-J13 and J13-J11
6GC/6A-seam	CCATCAATATTATATAGTAATAAGTATGAAAAAACGAGCGATG ATATTCAACCGTTTCAAATCA	Replaces H13-I12 and I12-H13
6GC/6A-staple	TATAACTAACAAAGAACGCGAGAACGCCACAGTCATAAAGAAT ATAAAGTACCAAGCCGT	Replaces J15-J13 and J13-J11
6GC/7A-seam	CCATCAATATTATATAGTAATAAGATGAAAAAAACGAGCGATG ATATTCAACCGTTTCAAATCA	Replaces H13-I12 and I12-H13
6GC/7A-staple	TATAACTAACAAAGAACGCGAGAACGCCCAGTCATAATAGAAT ATAAAGTACCAAGCCGT	Replaces J15-J13 and J13-J11
6GC/8A-seam	CCATCAATATTATATAGTAATAAGTGAAAAAAAACGAGCGATG ATATTCAACCGTTTCAAATCA	Replaces H13-I12 and I12-H13
6GC/8A-staple	TATAACTAACAAAGAACGCGAGAACGCCAGTCATAATAAGAAT ATAAAGTACCAAGCCGT	Replaces J15-J13 and J13-J11

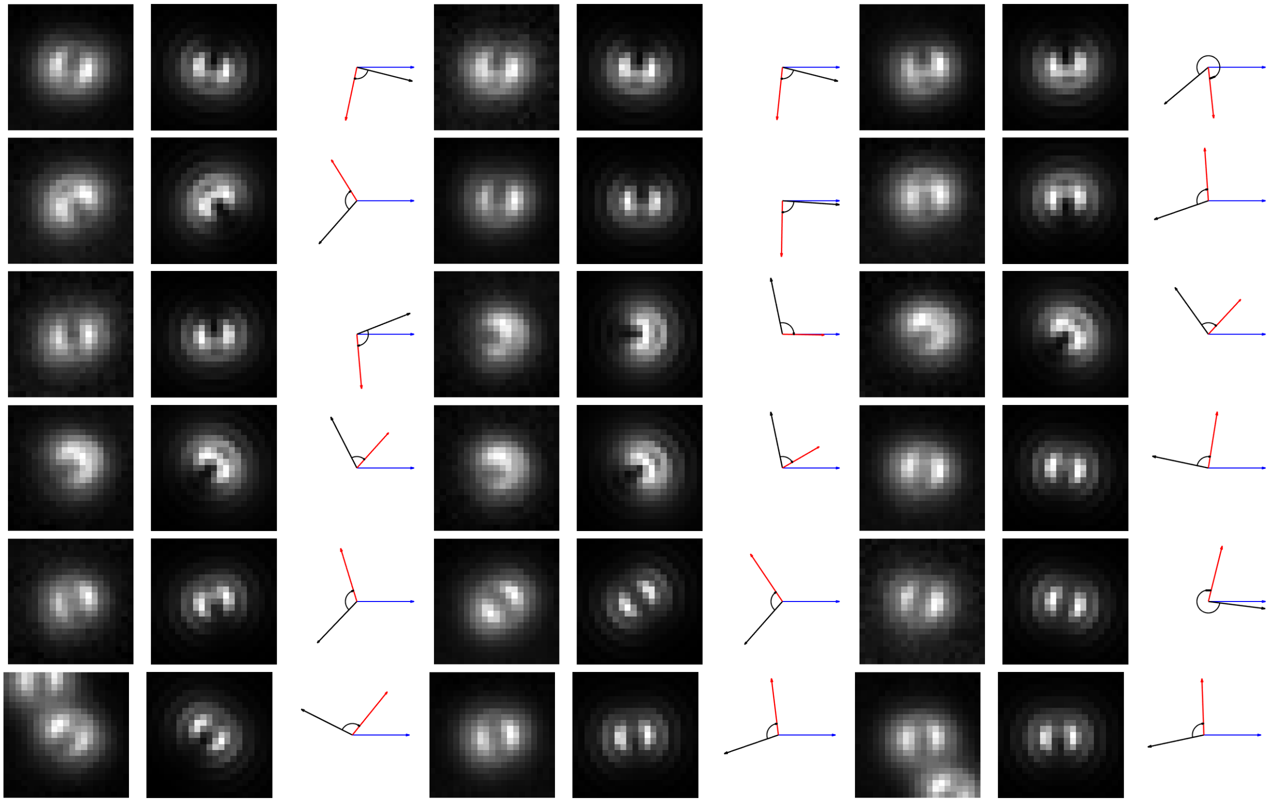

Figure S10: Each set of three sub-figures show the experimental dipole radiation pattern (left), simulated pattern (center) and in-plane orientations (right) in sample **5AA**. X axis, DNA origami, and in-plane dipole orientation are represented by the blue, black and red arrows, respectively. Z axis points towards the image. The in-plane dipole orientation is the projection of half of the double-headed arrow on the plane, and its length is $sin(\boldsymbol{\theta})$. The black curved arrow represents the angle of the dipole relative to the origami ($\boldsymbol{\phi}$). The lengths of the black and blue arrows are 1. The lengths of the black and blue arrows are 1. The defocused distance in the simulations was 550 nm.

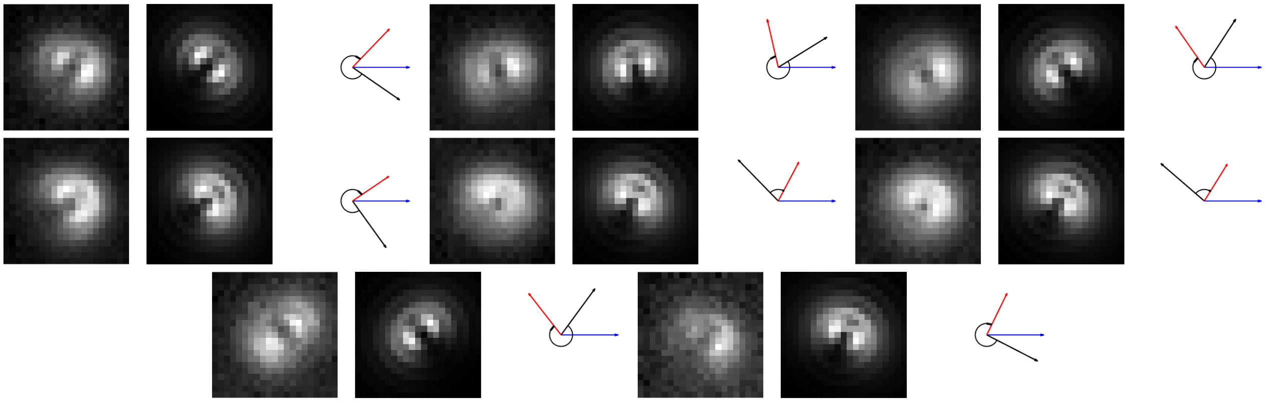

Figure S11: Each set of three sub-figures show the experimental dipole radiation pattern (left), simulated pattern (center) and in-plane orientations (right) in sample **5GC**. X axis, DNA origami, and in-plane dipole orientation are represented by the blue, black and red arrows, respectively. Z axis points towards the image. The in-plane dipole orientation is the projection of half of the double-headed arrow on the plane, and its length is $sin(\boldsymbol{\theta})$. The black curved arrow represents the angle of the dipole relative to the origami ($\boldsymbol{\phi}$). The lengths of the black and blue arrows are 1. The lengths of the black and blue arrows are 1. The defocused distance in the simulations was 600 nm.

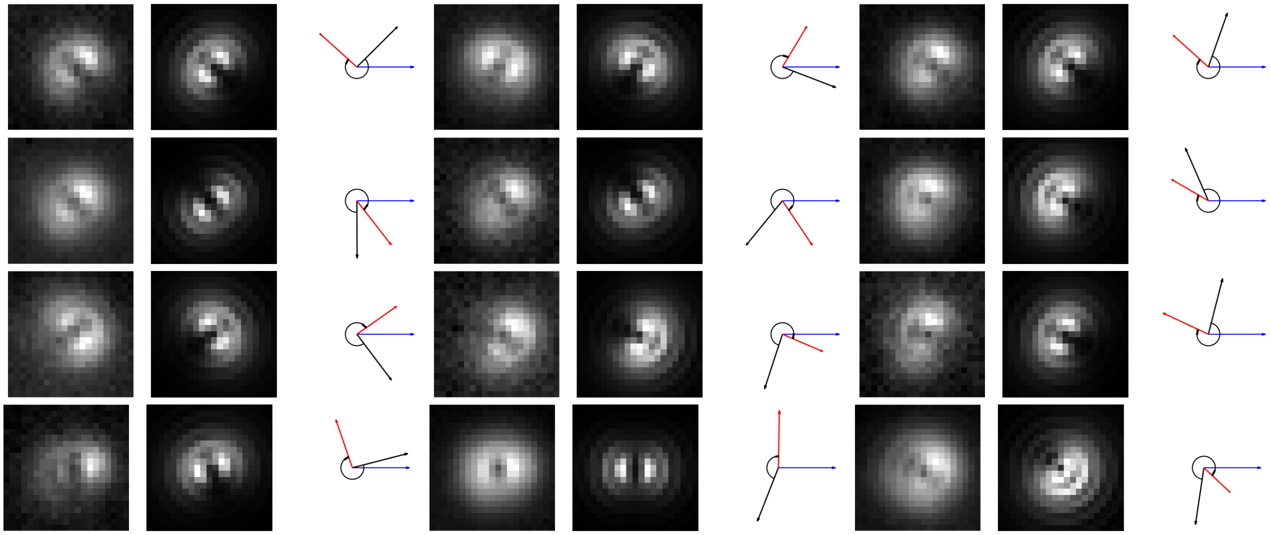

Figure S12: Each set of three sub-figures show the experimental dipole radiation pattern (left), simulated pattern (center) and in-plane orientations (right) in sample **5TT**. X axis, DNA origami, and in-plane dipole orientation are represented by the blue, black and red arrows, respectively. Z axis points towards the image. The in-plane dipole orientation is the projection of half of the double-headed arrow on the plane, and its length is $sin(\boldsymbol{\theta})$. The black curved arrow represents the angle of the dipole relative to the origami ($\boldsymbol{\phi}$). The lengths of the black and blue arrows are 1. The lengths of the black and blue arrows are 1. The defocused distance in the simulations was 600 nm.

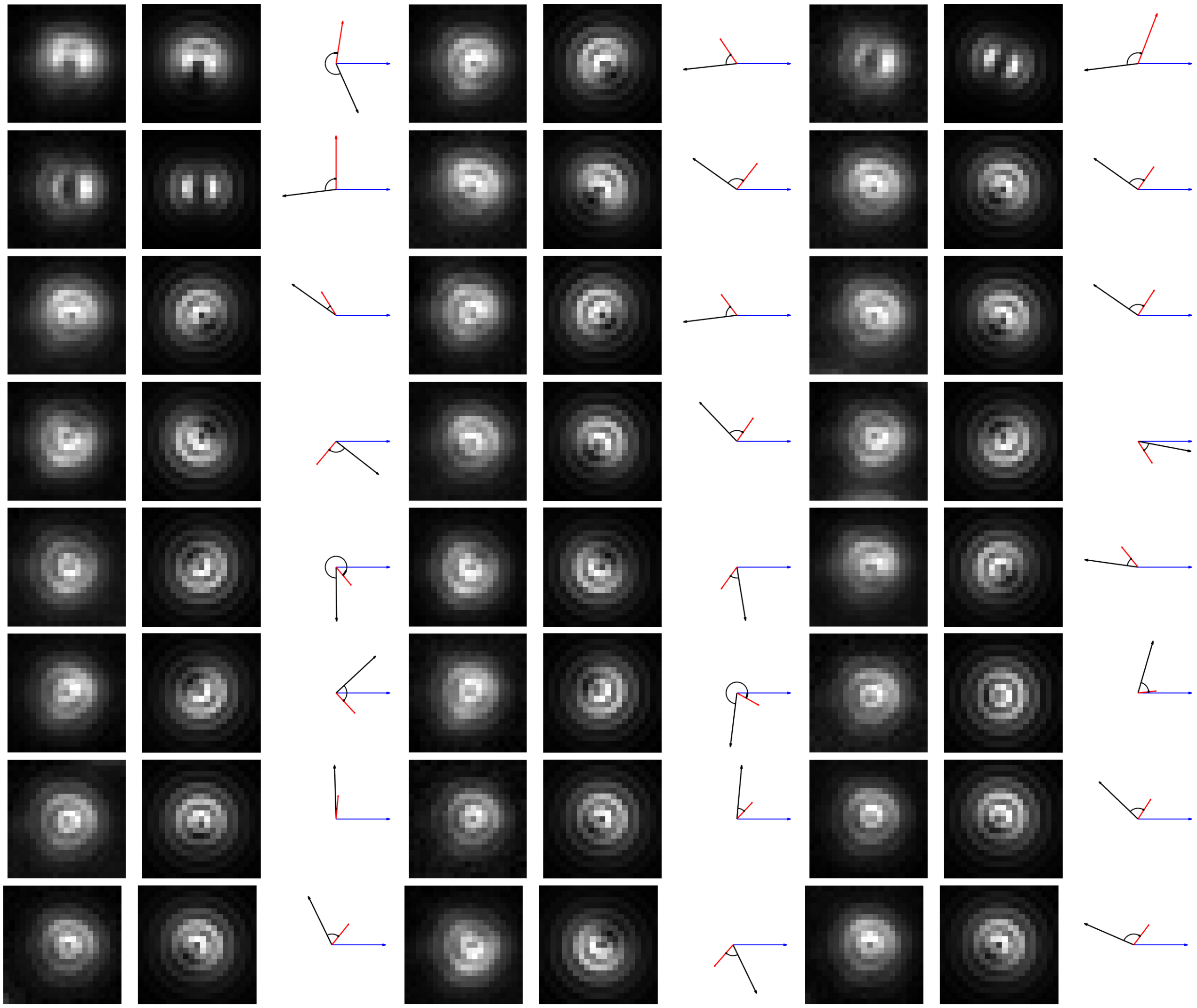

Figure S13: Each set of three sub-figures show the experimental dipole radiation pattern (left), simulated pattern (center) and in-plane orientations (right) in sample **6AA**. X axis, DNA origami, and in-plane dipole orientation are represented by the blue, black and red arrows, respectively. Z axis points towards the image. The in-plane dipole orientation is the projection of half of the double-headed arrow on the plane, and its length is $sin(\boldsymbol{\theta})$. The black curved arrow represents the angle of the dipole relative to the origami ($\boldsymbol{\phi}$). The lengths of the black and blue arrows are 1. The defocused distance in the simulations was 575 nm.

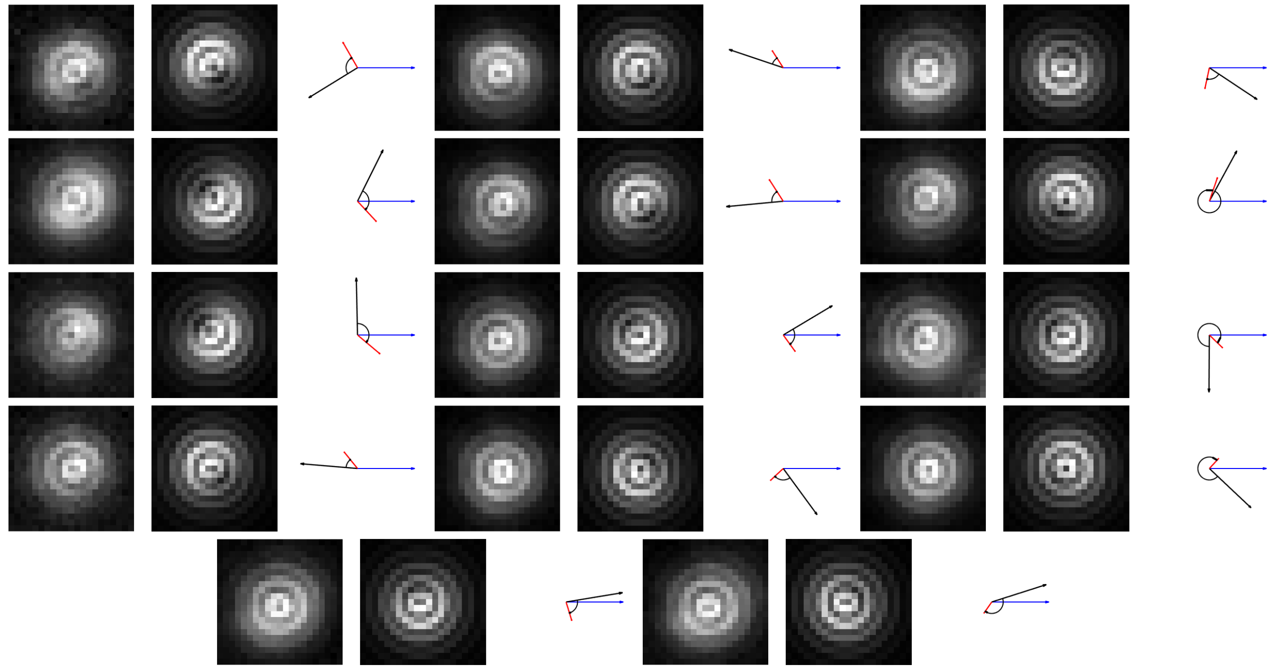

Figure S14: Each set of three sub-figures show the experimental dipole radiation pattern (left), simulated pattern (center) and in-plane orientations (right) in sample **6GC**. X axis, DNA origami, and in-plane dipole orientation are represented by the blue, black and red arrows, respectively. Z axis points towards the image. The in-plane dipole orientation is the projection of half of the double-headed arrow on the plane, and its length is $sin(\boldsymbol{\theta})$. The black curved arrow represents the angle of the dipole relative to the origami ($\boldsymbol{\phi}$). The lengths of the black and blue arrows are 1. The defocused distance in the simulations was 600 nm.

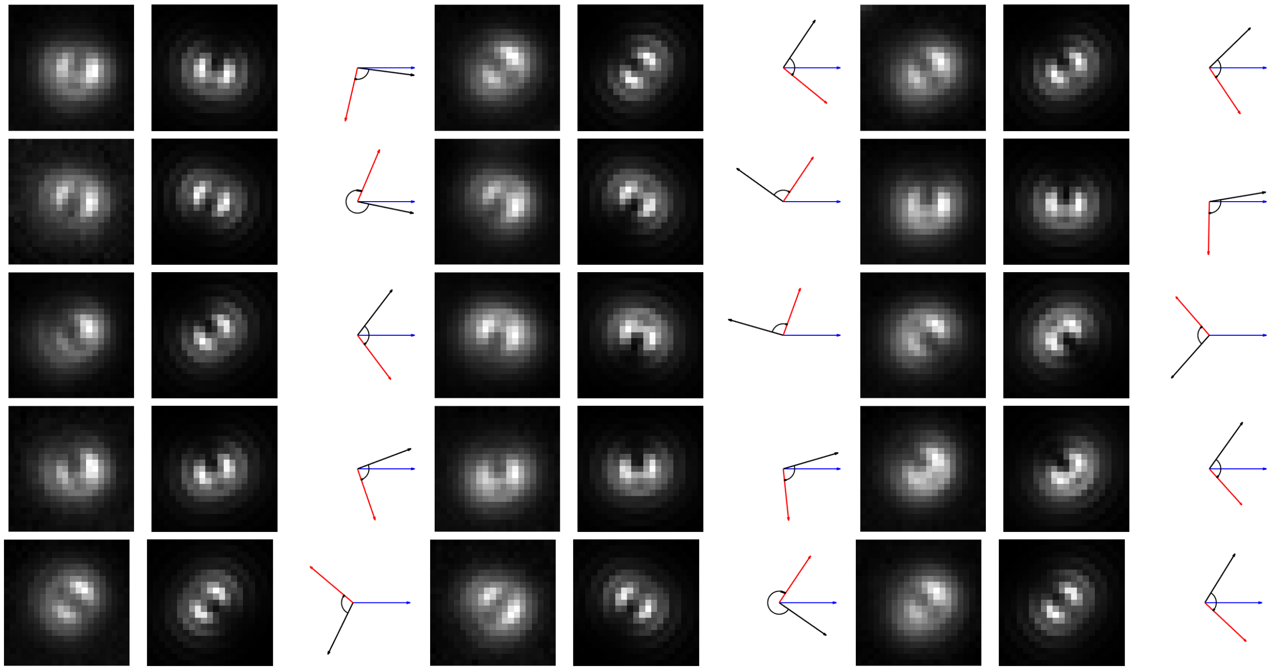

Figure S15: Each set of three sub-figures show the experimental dipole radiation pattern (left), simulated pattern (center) and in-plane orientations (right) in sample **6TT**. X axis, DNA origami, and in-plane dipole orientation are represented by the blue, black and red arrows, respectively. Z axis points towards the image. The in-plane dipole orientation is the projection of half of the double-headed arrow on the plane, and its length is $sin(\boldsymbol{\theta})$. The black curved arrow represents the angle of the dipole relative to the origami ($\boldsymbol{\phi}$). The lengths of the black and blue arrows are 1. The defocused distance in the simulations was 550 nm.

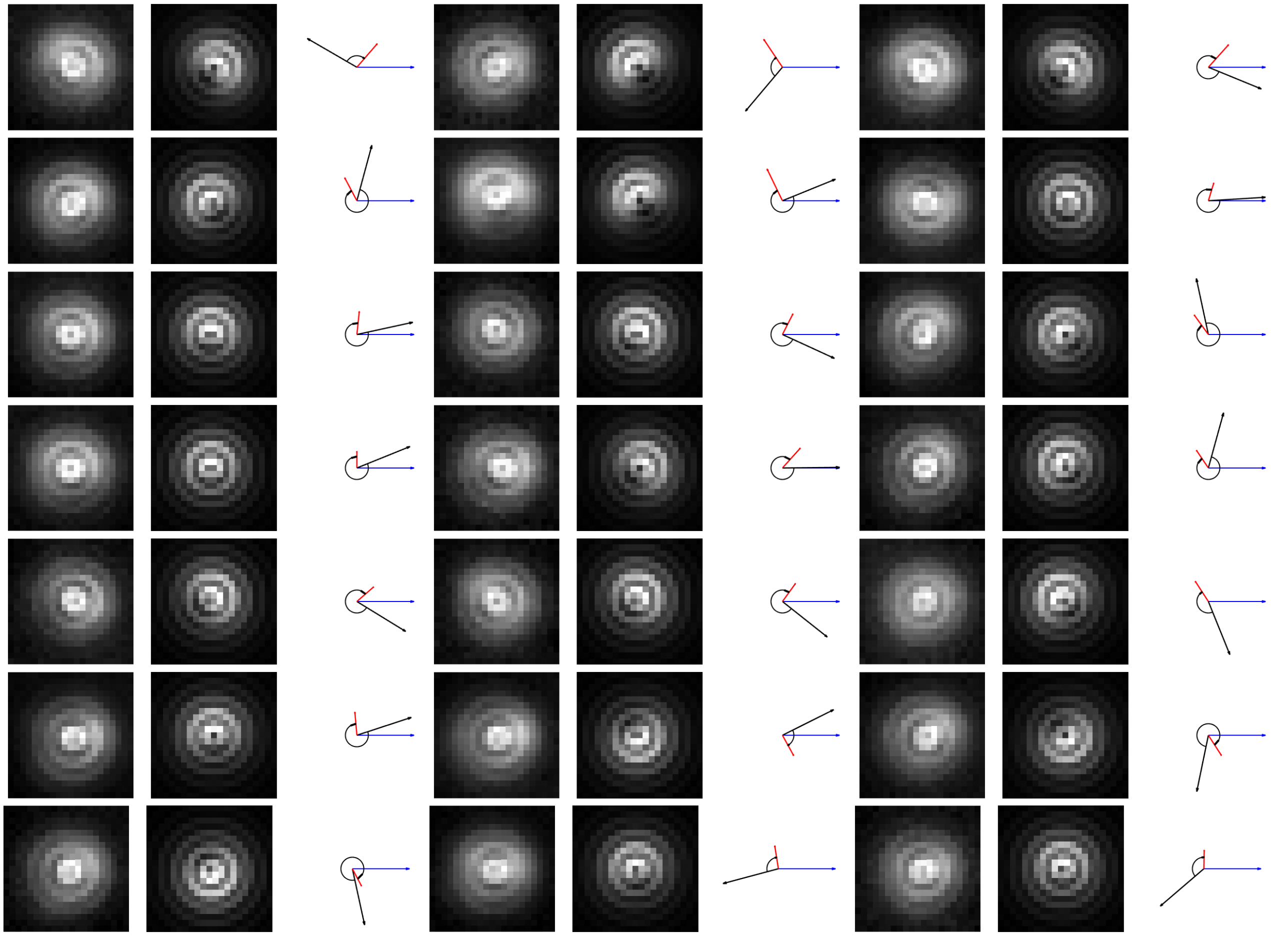

Figure S16: Each set of three sub-figures show the experimental dipole radiation pattern (left), simulated pattern (center) and in-plane orientations (right) in sample **7AA**. X axis, DNA origami, and in-plane dipole orientation are represented by the blue, black and red arrows, respectively. Z axis points towards the image. The in-plane dipole orientation is the projection of half of the double-headed arrow on the plane, and its length is $sin(\boldsymbol{\theta})$. The black curved arrow represents the angle of the dipole relative to the origami ($\boldsymbol{\phi}$). The lengths of the black and blue arrows are 1. The defocused distance in the simulations was 575 nm.

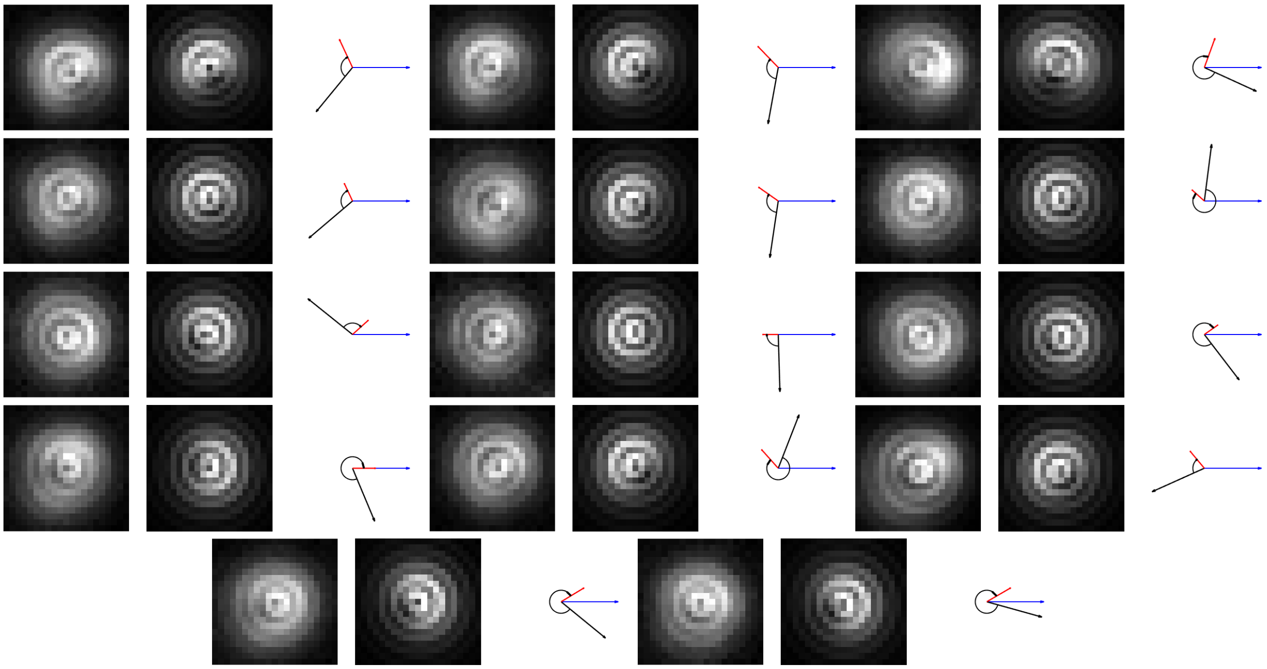

Figure S17: Each set of three sub-figures show the experimental dipole radiation pattern (left), simulated pattern (center) and in-plane orientations (right) in sample **7GC**. X axis, DNA origami, and in-plane dipole orientation are represented by the blue, black and red arrows, respectively. Z axis points towards the image. The in-plane dipole orientation is the projection of half of the double-headed arrow on the plane, and its length is $sin(\boldsymbol{\theta})$. The black curved arrow represents the angle of the dipole relative to the origami ($\boldsymbol{\phi}$). The lengths of the black and blue arrows are 1. The defocused distance in the simulations was 600 nm.

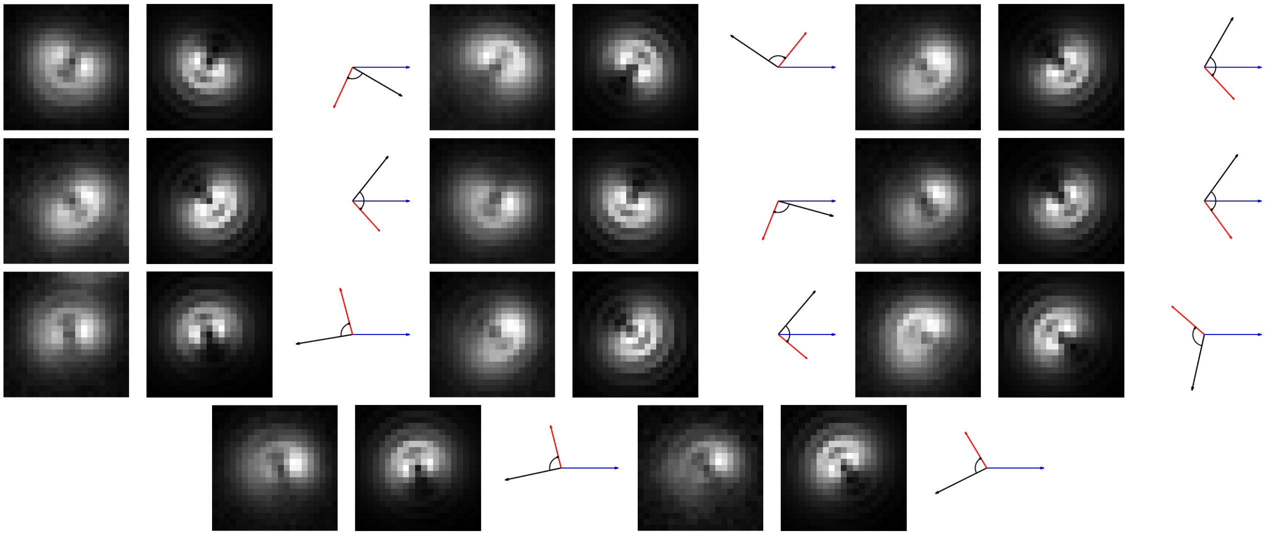

Figure S18: Each set of three sub-figures show the experimental dipole radiation pattern (left), simulated pattern (center) and in-plane orientations (right) in sample **7TT**. X axis, DNA origami, and in-plane dipole orientation are represented by the blue, black and red arrows, respectively. Z axis points towards the image. The in-plane dipole orientation is the projection of half of the double-headed arrow on the plane, and its length is $sin(\boldsymbol{\theta})$. The black curved arrow represents the angle of the dipole relative to the origami ($\boldsymbol{\phi}$). The lengths of the black and blue arrows are 1. The defocused distance in the simulations was 600 nm.

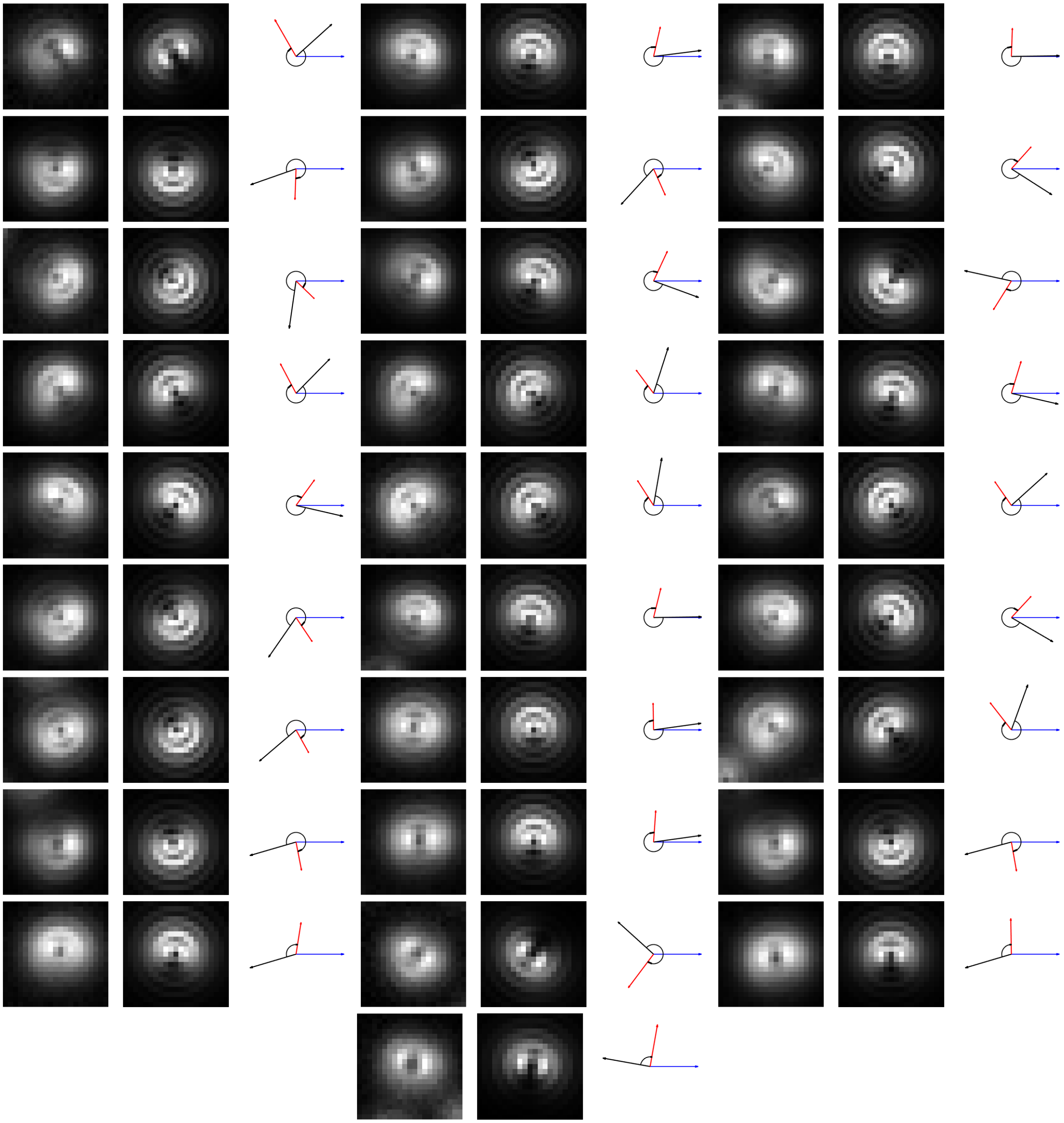

Figure S19: Each set of three sub-figures show the experimental dipole radiation pattern (left), simulated pattern (center) and in-plane orientations (right) in sample **8AA**. X axis, DNA origami, and in-plane dipole orientation are represented by the blue, black and red arrows, respectively. Z axis points towards the image. The in-plane dipole orientation is the projection of half of the double-headed arrow on the plane, and its length is $sin(\boldsymbol{\theta})$. The black curved arrow represents the angle of the dipole relative to the origami ($\boldsymbol{\phi}$). The lengths of the black and blue arrows are 1. The defocused distance in the simulations was 600 nm.

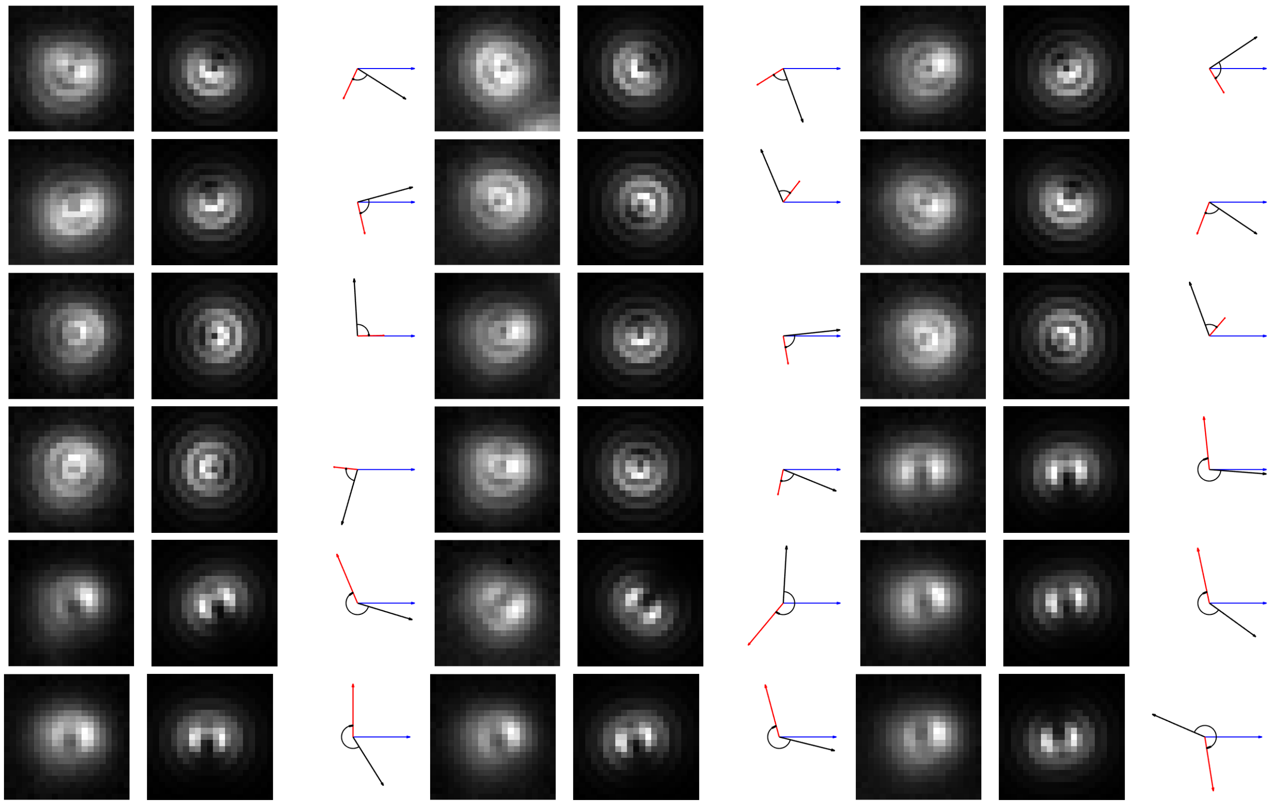

Figure S20: Each set of three sub-figures show the experimental dipole radiation pattern (left), simulated pattern (center) and in-plane orientations (right) in sample **8TT**. X axis, DNA origami, and in-plane dipole orientation are represented by the blue, black and red arrows, respectively. Z axis points towards the image. The in-plane dipole orientation is the projection of half of the double-headed arrow on the plane, and its length is $sin(\boldsymbol{\theta})$. The black curved arrow represents the angle of the dipole relative to the origami ($\boldsymbol{\phi}$). The lengths of the black and blue arrows are 1. The defocused distance in the simulations was 550 nm.

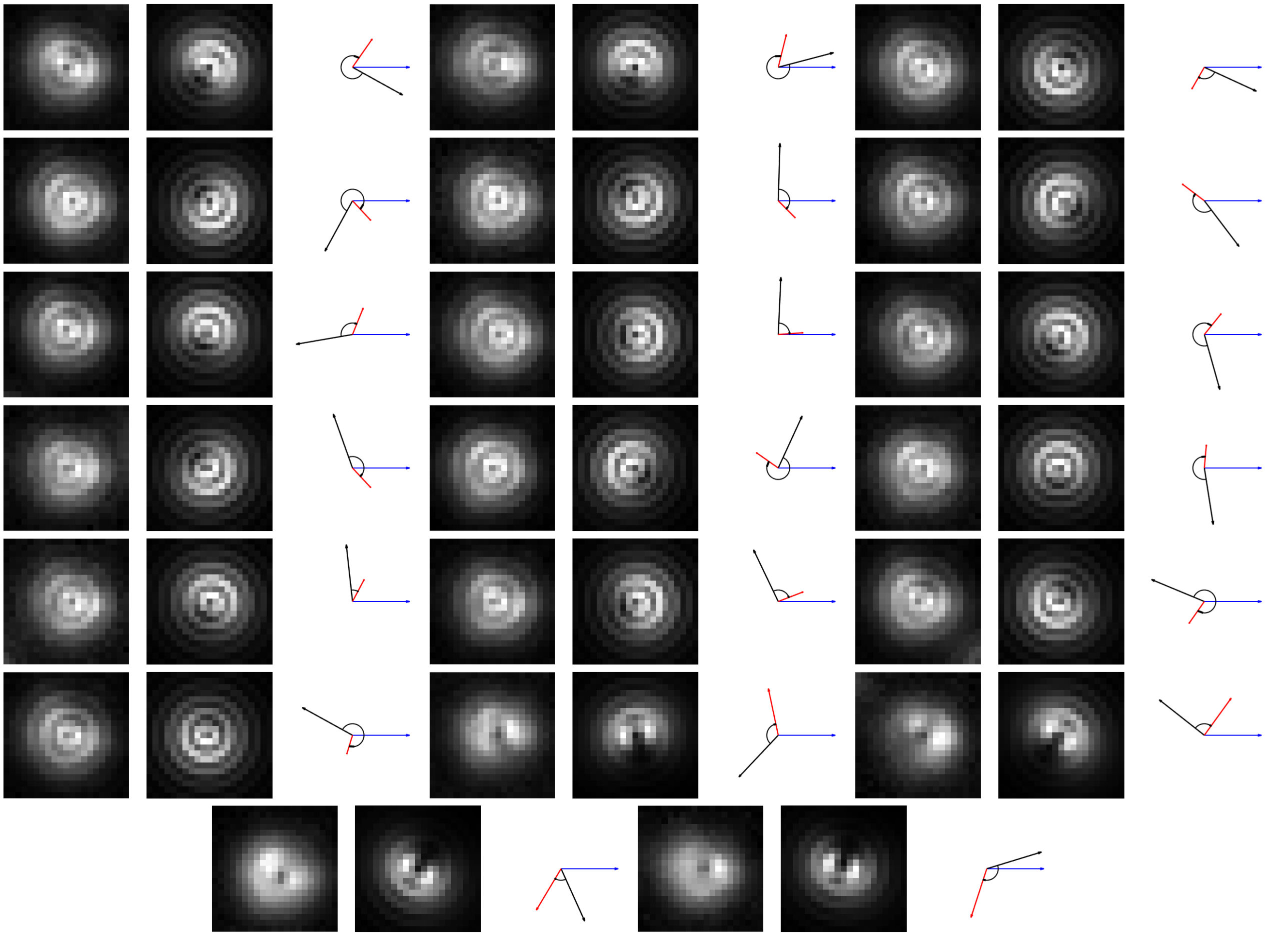

Figure S21: Each set of three sub-figures show the experimental dipole radiation pattern (left), simulated pattern (center) and in-plane orientations (right) in sample **8GC**. X axis, DNA origami, and in-plane dipole orientation are represented by the blue, black and red arrows, respectively. Z axis points towards the image. The in-plane dipole orientation is the projection of half of the double-headed arrow on the plane, and its length is $sin(\boldsymbol{\theta})$. The black curved arrow represents the angle of the dipole relative to the origami ($\boldsymbol{\phi}$). The lengths of the black and blue arrows are 1. The defocused distance in the simulations was 600 nm.

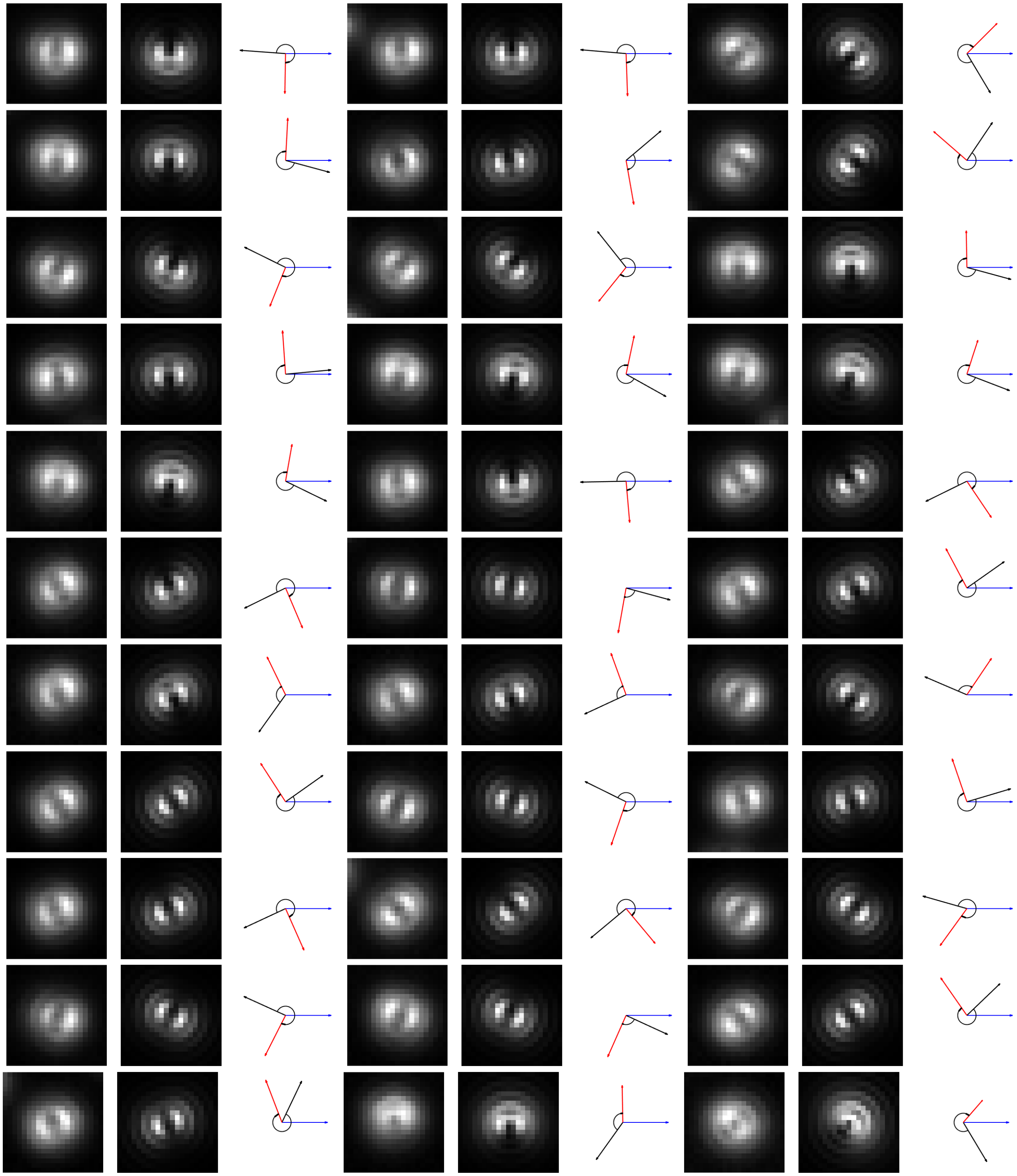

Figure S22: Each set of three sub-figures show the experimental dipole radiation pattern (left), simulated pattern (center) and in-plane orientations (right) in sample **9AA**. X axis, DNA origami, and in-plane dipole orientation are represented by the blue, black and red arrows, respectively. Z axis points towards the image. The in-plane dipole orientation is the projection of half of the double-headed arrow on the plane, and its length is $sin(\boldsymbol{\theta})$. The black curved arrow represents the angle of the dipole relative to the origami ($\boldsymbol{\phi}$). The lengths of the black and blue arrows are 1. The defocused distance in the simulations was 575 nm.

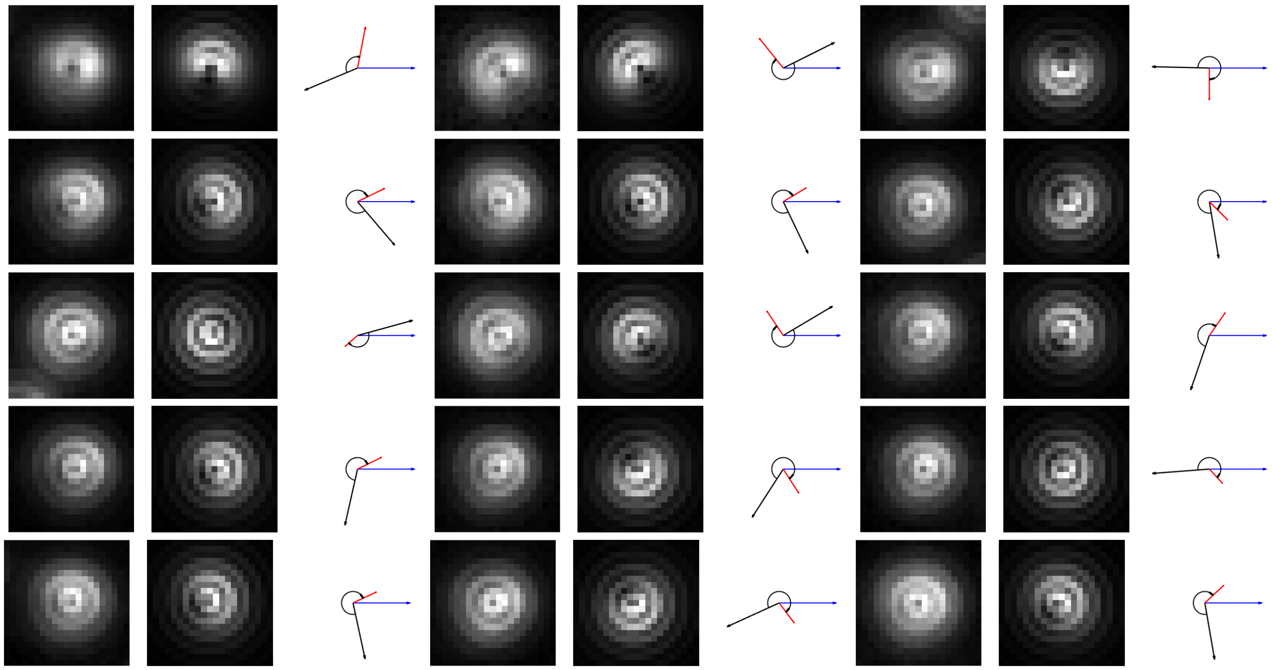

Figure S23: Each set of three sub-figures show the experimental dipole radiation pattern (left), simulated pattern (center) and in-plane orientations (right) in sample **9GC**. X axis, DNA origami, and in-plane dipole orientation are represented by the blue, black and red arrows, respectively. Z axis points towards the image. The in-plane dipole orientation is the projection of half of the double-headed arrow on the plane, and its length is $sin(\boldsymbol{\theta})$. The black curved arrow represents the angle of the dipole relative to the origami ($\boldsymbol{\phi}$). The lengths of the black and blue arrows are 1. The defocused distance in the simulations was 575 nm.

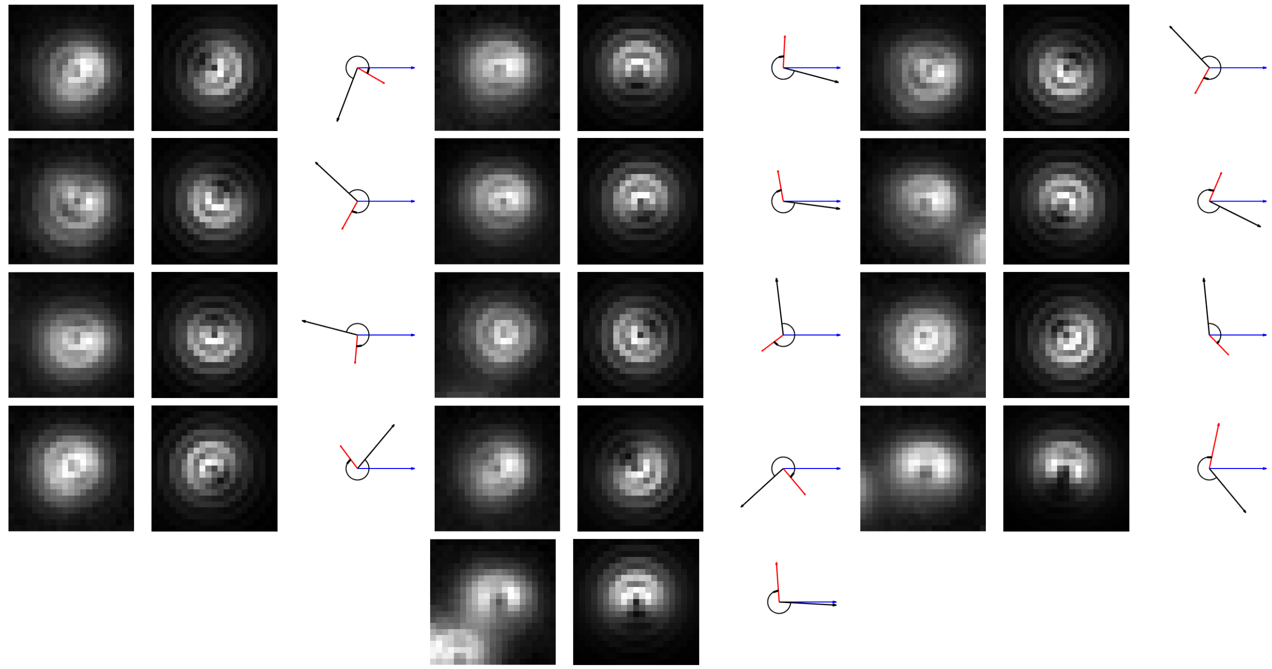

Figure S24: Each set of three sub-figures show the experimental dipole radiation pattern (left), simulated pattern (center) and in-plane orientations (right) in sample **9TT**. X axis, DNA origami, and in-plane dipole orientation are represented by the blue, black and red arrows, respectively. Z axis points towards the image. The in-plane dipole orientation is the projection of half of the double-headed arrow on the plane, and its length is $sin(\boldsymbol{\theta})$. The black curved arrow represents the angle of the dipole relative to the origami ($\boldsymbol{\phi}$). The lengths of the black and blue arrows are 1. The defocused distance in the simulations was 575 nm.

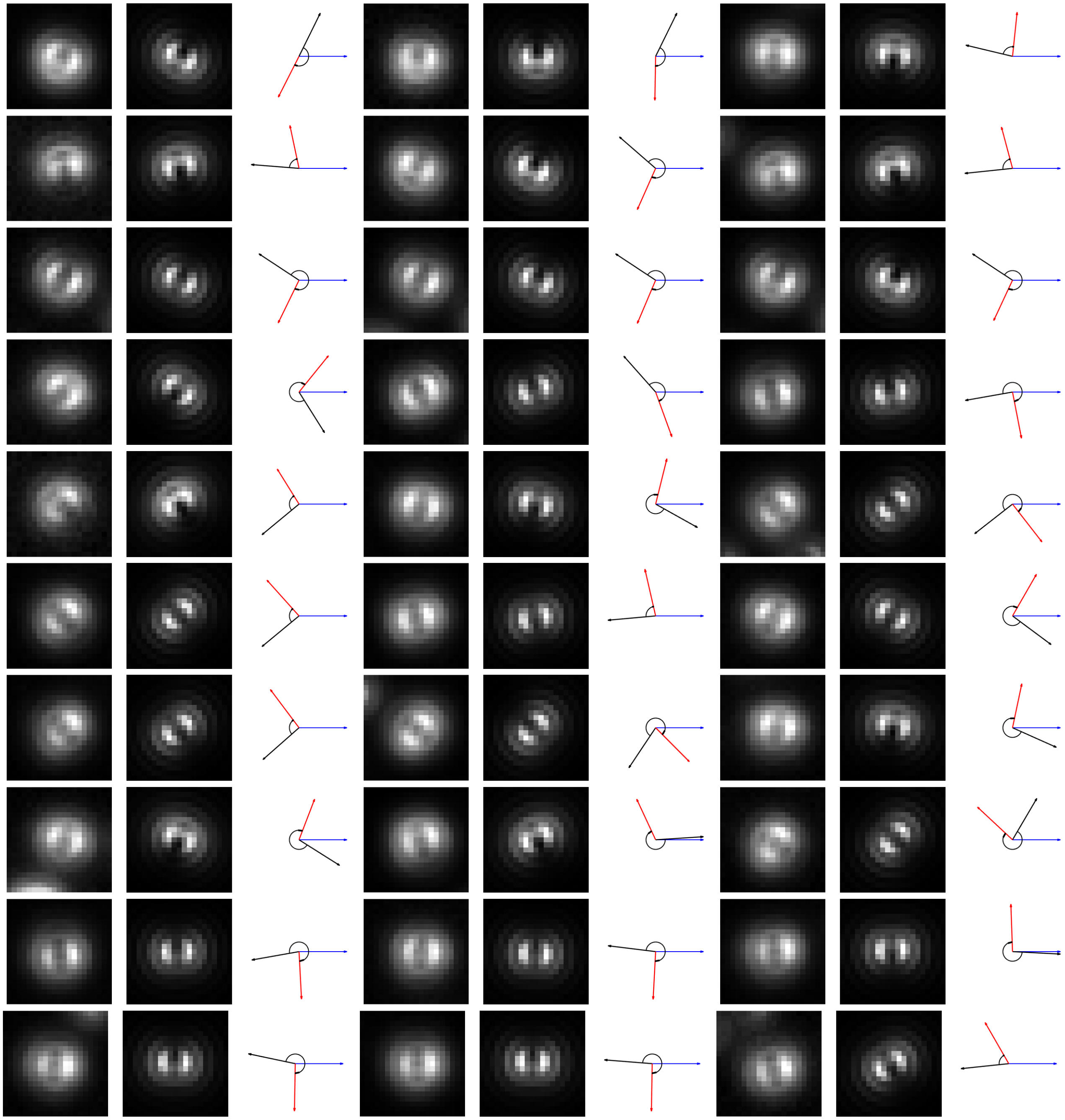

Figure S25: Each set of three sub-figures show the experimental dipole radiation pattern (left), simulated pattern (center) and in-plane orientations (right) in sample **10AA**. X axis, DNA origami, and in-plane dipole orientation are represented by the blue, black and red arrows, respectively. Z axis points towards the image. The in-plane dipole orientation is the projection of half of the double-headed arrow on the plane, and its length is $sin(\boldsymbol{\theta})$. The black curved arrow represents the angle of the dipole relative to the origami ($\boldsymbol{\phi}$). The lengths of the black and blue arrows are 1. The defocused distance in the simulations was 550 nm.

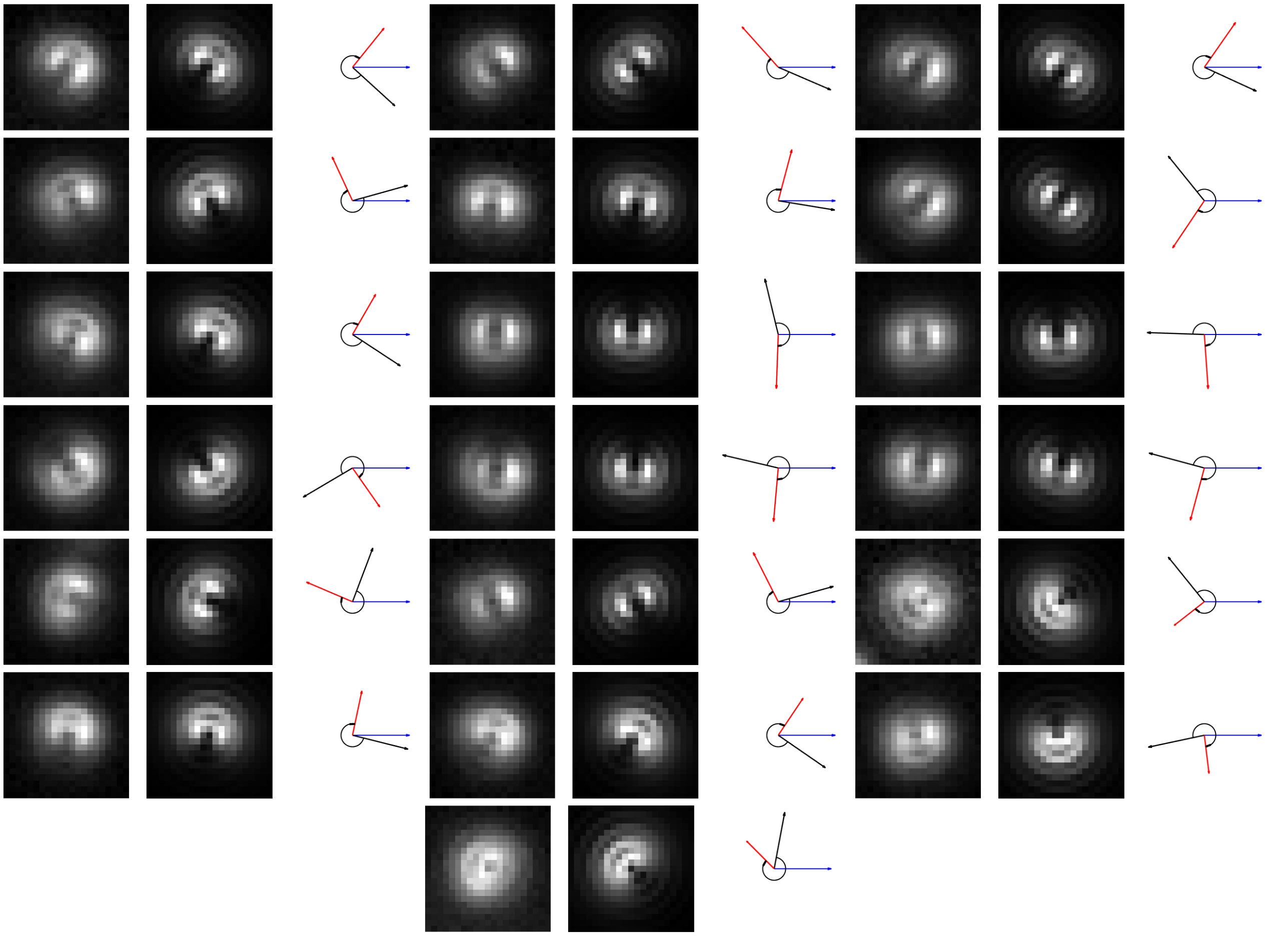

Figure S26: Each set of three sub-figures show the experimental dipole radiation pattern (left), simulated pattern (center) and in-plane orientations (right) in sample **10GC**. X axis, DNA origami, and in-plane dipole orientation are represented by the blue, black and red arrows, respectively. Z axis points towards the image. The in-plane dipole orientation is the projection of half of the double-headed arrow on the plane, and its length is $sin(\boldsymbol{\theta})$. The black curved arrow represents the angle of the dipole relative to the origami ($\boldsymbol{\phi}$). The lengths of the black and blue arrows are 1. The defocused distance in the simulations was 575 nm.

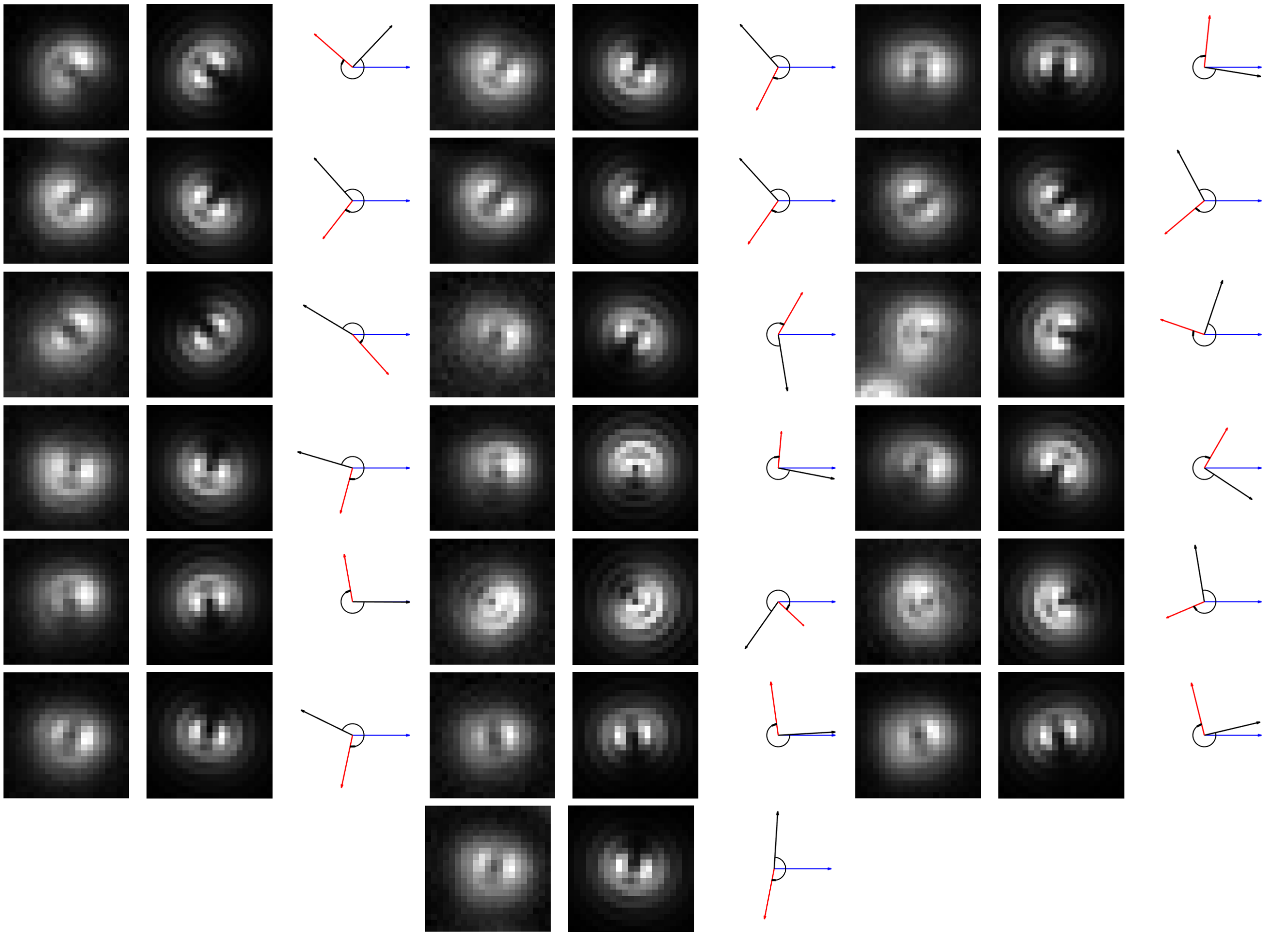

Figure S27: Each set of three sub-figures show the experimental dipole radiation pattern (left), simulated pattern (center) and in-plane orientations (right) in sample **10TT**. X axis, DNA origami, and in-plane dipole orientation are represented by the blue, black and red arrows, respectively. Z axis points towards the image. The in-plane dipole orientation is the projection of half of the double-headed arrow on the plane, and its length is $sin(\boldsymbol{\theta})$. The black curved arrow represents the angle of the dipole relative to the origami ($\boldsymbol{\phi}$). The lengths of the black and blue arrows are 1. The defocused distance in the simulations was 600 nm.

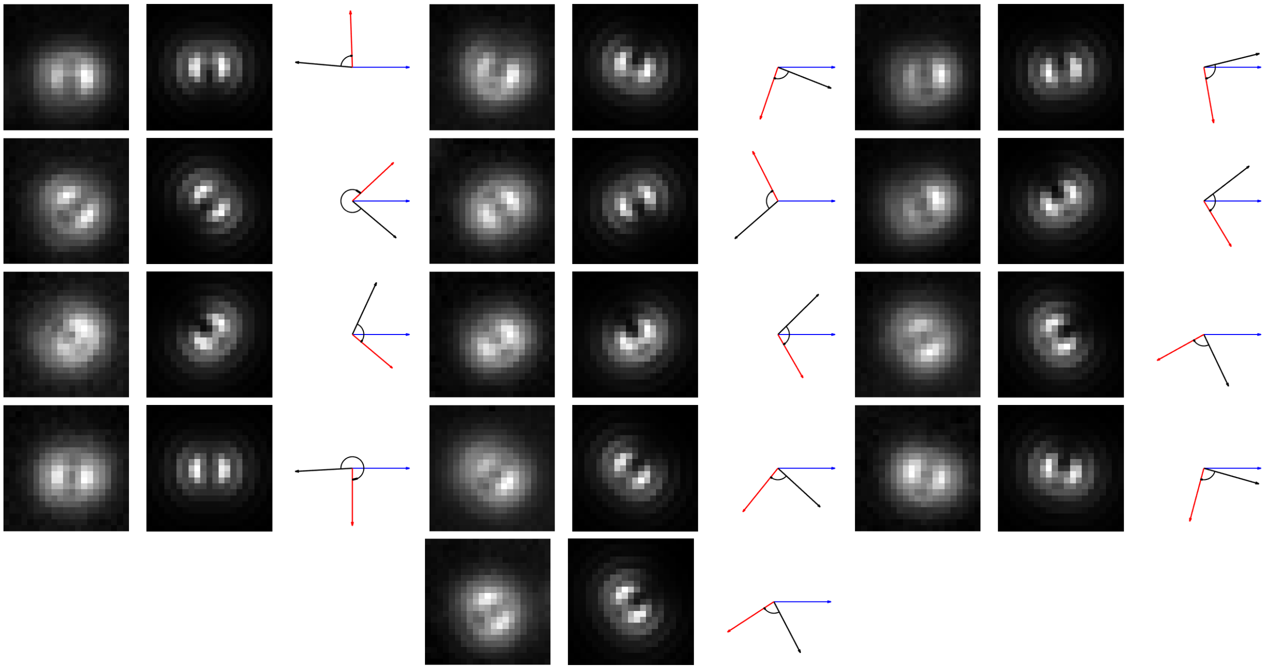

Figure S28: Each set of three sub-figures show the experimental dipole radiation pattern (left), simulated pattern (center) and in-plane orientations (right) in sample **6GC/1A**. X axis, DNA origami, and in-plane dipole orientation are represented by the blue, black and red arrows, respectively. Z axis points towards the image. The in-plane dipole orientation is the projection of half of the double-headed arrow on the plane, and its length is $sin(\boldsymbol{\theta})$. The black curved arrow represents the angle of the dipole relative to the origami ($\boldsymbol{\phi}$). The lengths of the black and blue arrows are 1. The defocused distance in the simulations was 550 nm.

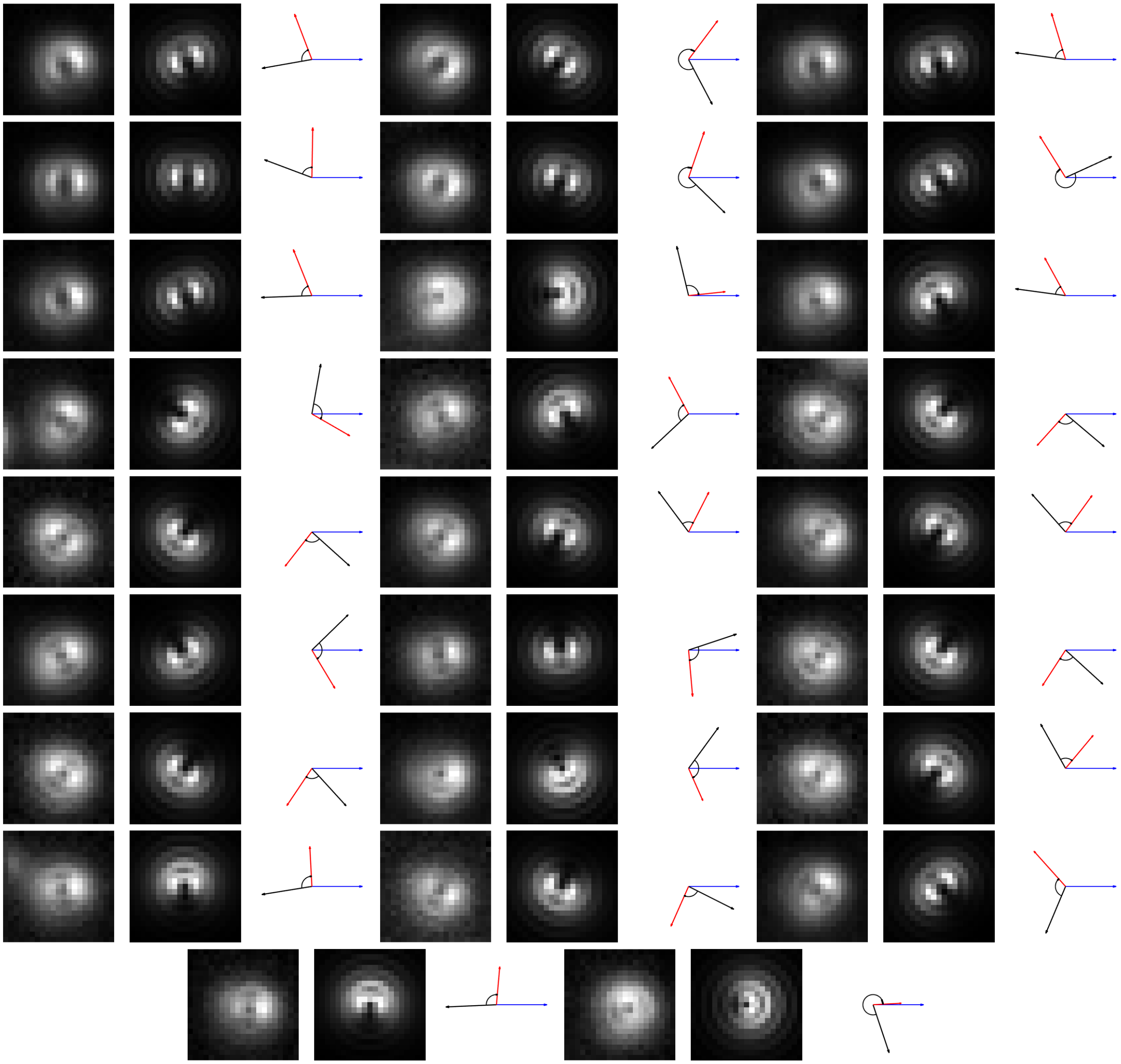

Figure S29: Each set of three sub-figures show the experimental dipole radiation pattern (left), simulated pattern (center) and in-plane orientations (right) in sample **6GC/2A**. X axis, DNA origami, and in-plane dipole orientation are represented by the blue, black and red arrows, respectively. Z axis points towards the image. The in-plane dipole orientation is the projection of half of the double-headed arrow on the plane, and its length is $sin(\boldsymbol{\theta})$. The black curved arrow represents the angle of the dipole relative to the origami ($\boldsymbol{\phi}$). The lengths of the black and blue arrows are 1. The defocused distance in the simulations was 575 nm.

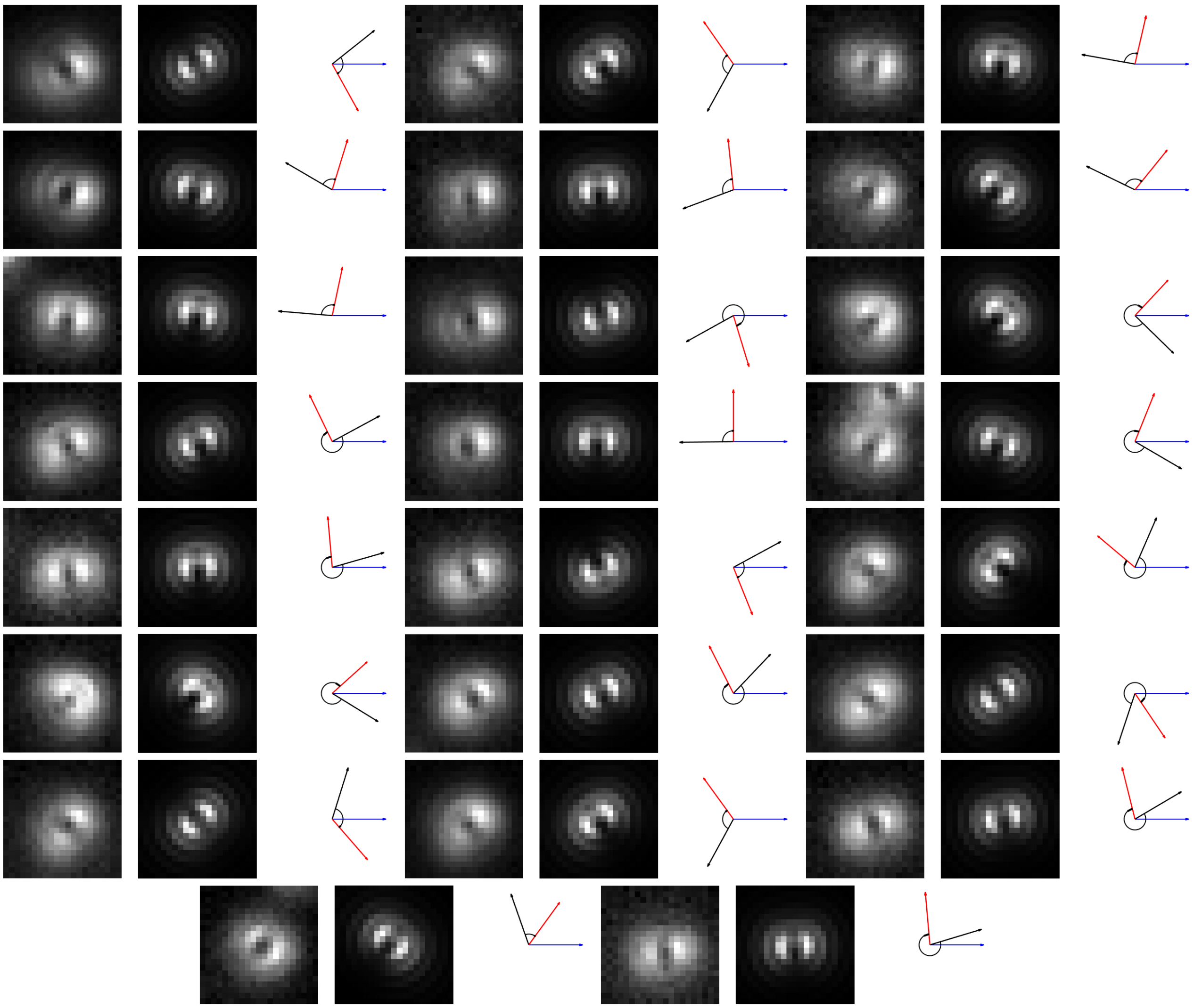

Figure S30: Each set of three sub-figures show the experimental dipole radiation pattern (left), simulated pattern (center) and in-plane orientations (right) in sample **6GC/3A**. X axis, DNA origami, and in-plane dipole orientation are represented by the blue, black and red arrows, respectively. Z axis points towards the image. The in-plane dipole orientation is the projection of half of the double-headed arrow on the plane, and its length is $sin(\boldsymbol{\theta})$. The black curved arrow represents the angle of the dipole relative to the origami ($\boldsymbol{\phi}$). The lengths of the black and blue arrows are 1. The defocused distance in the simulations was 550 nm.

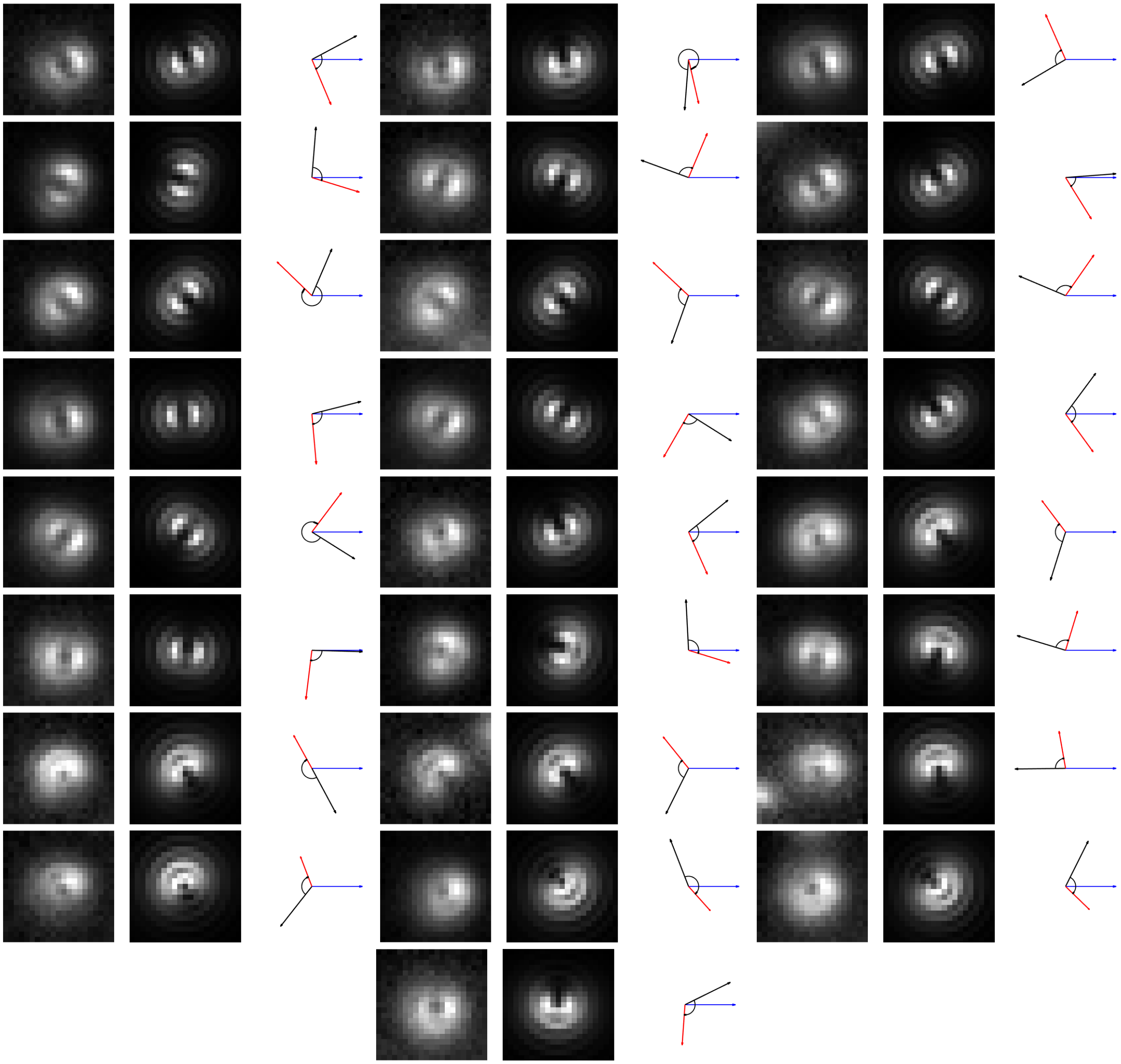

Figure S31: Each set of three sub-figures show the experimental dipole radiation pattern (left), simulated pattern (center) and in-plane orientations (right) in sample **6GC/4A**. X axis, DNA origami, and in-plane dipole orientation are represented by the blue, black and red arrows, respectively. Z axis points towards the image. The in-plane dipole orientation is the projection of half of the double-headed arrow on the plane, and its length is $sin(\boldsymbol{\theta})$. The black curved arrow represents the angle of the dipole relative to the origami ($\boldsymbol{\phi}$). The lengths of the black and blue arrows are 1. The defocused distance in the simulations was 575 nm.

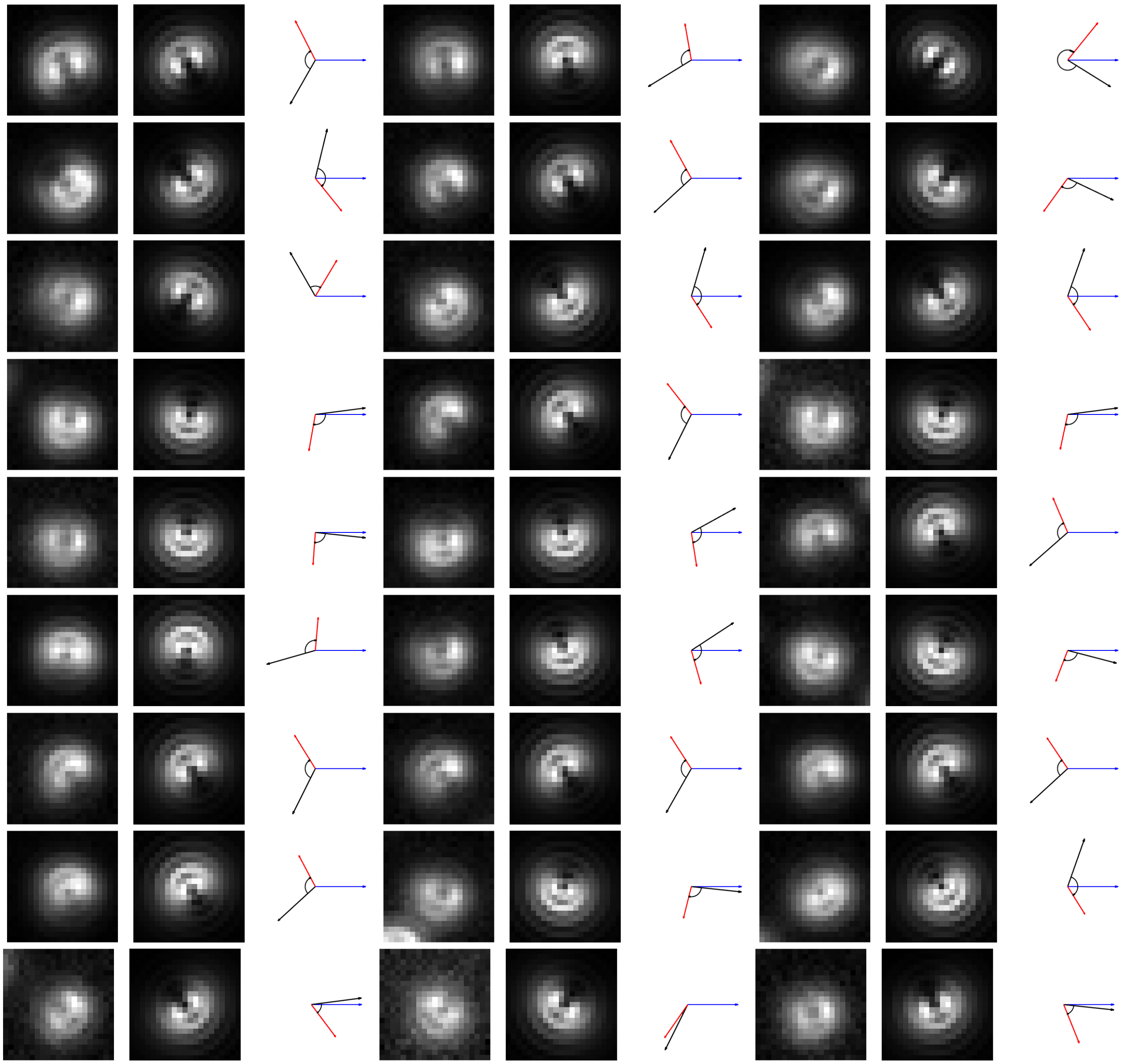

Figure S32: Each set of three sub-figures show the experimental dipole radiation pattern (left), simulated pattern (center) and in-plane orientations (right) in sample **6GC/5A**. X axis, DNA origami, and in-plane dipole orientation are represented by the blue, black and red arrows, respectively. Z axis points towards the image. The in-plane dipole orientation is the projection of half of the double-headed arrow on the plane, and its length is $sin(\boldsymbol{\theta})$. The black curved arrow represents the angle of the dipole relative to the origami ($\boldsymbol{\phi}$). The lengths of the black and blue arrows are 1. The defocused distance in the simulations was 600 nm.

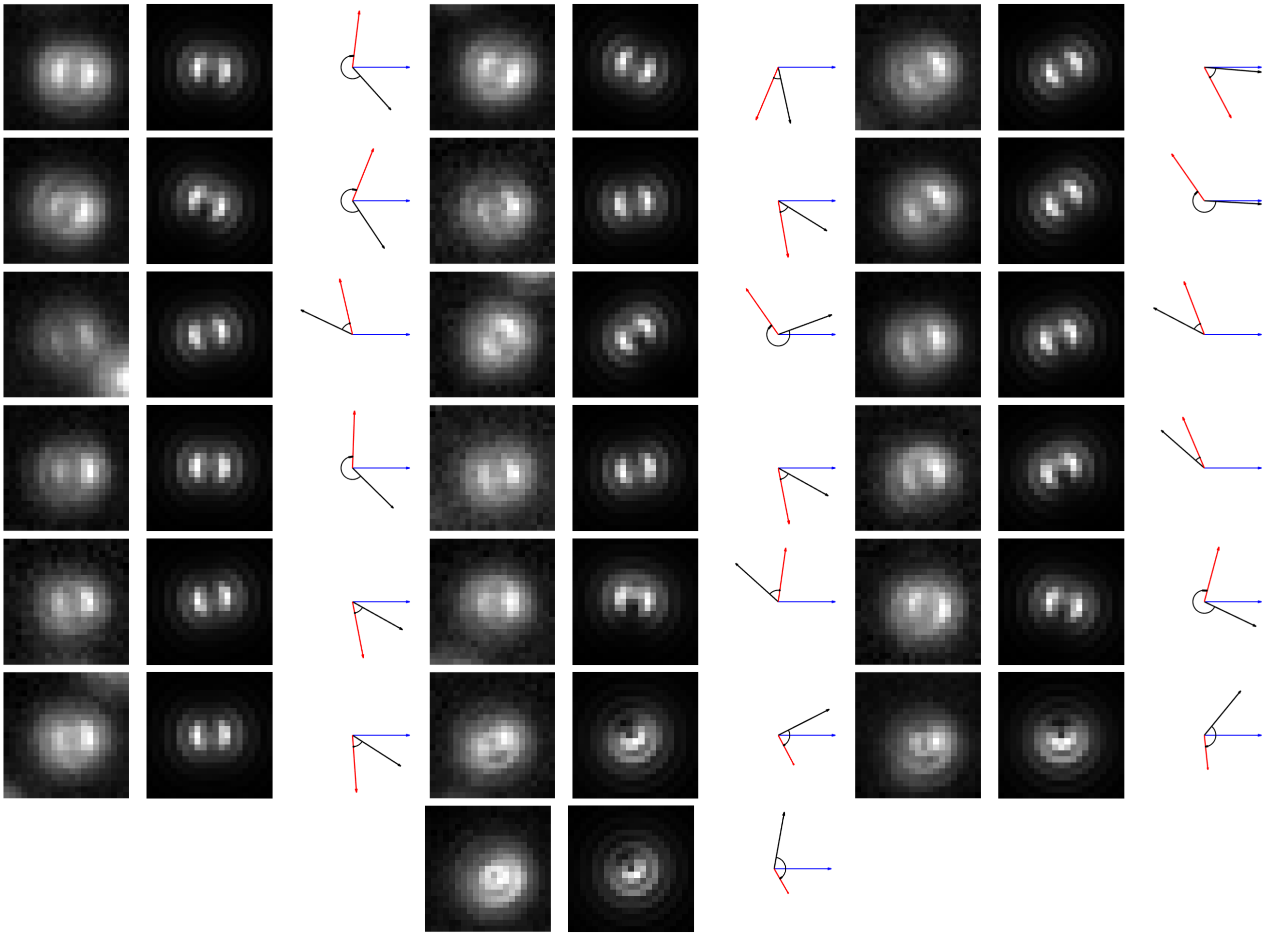

Figure S33: Each set of three sub-figures show the experimental dipole radiation pattern (left), simulated pattern (center) and in-plane orientations (right) in sample **6GC/6A**. X axis, DNA origami, and in-plane dipole orientation are represented by the blue, black and red arrows, respectively. Z axis points towards the image. The in-plane dipole orientation is the projection of half of the double-headed arrow on the plane, and its length is $sin(\boldsymbol{\theta})$. The black curved arrow represents the angle of the dipole relative to the origami ($\boldsymbol{\phi}$). The lengths of the black and blue arrows are 1. The defocused distance in the simulations was 525 nm.

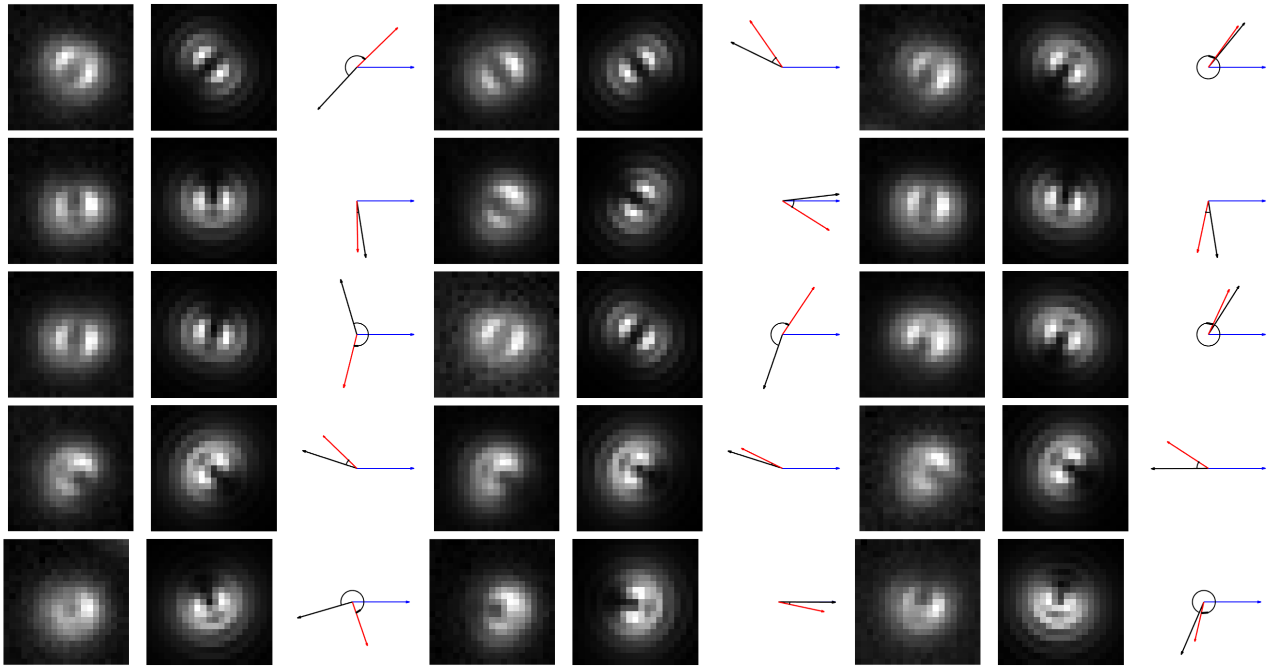

Figure S34: Each set of three sub-figures show the experimental dipole radiation pattern (left), simulated pattern (center) and in-plane orientations (right) in sample **6GC/7A**. X axis, DNA origami, and in-plane dipole orientation are represented by the blue, black and red arrows, respectively. Z axis points towards the image. The in-plane dipole orientation is the projection of half of the double-headed arrow on the plane, and its length is $sin(\boldsymbol{\theta})$. The black curved arrow represents the angle of the dipole relative to the origami ($\boldsymbol{\phi}$). The lengths of the black and blue arrows are 1. The defocused distance in the simulations was 600 nm.

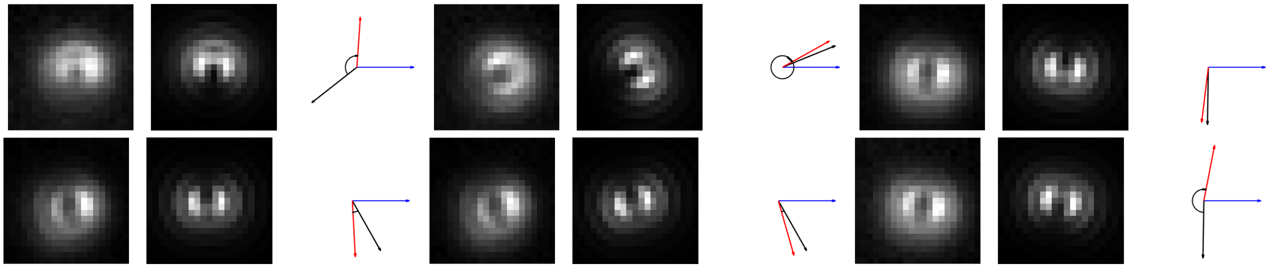

Figure S35: Each set of three sub-figures show the experimental dipole radiation pattern (left), simulated pattern (center) and in-plane orientations (right) in sample **6GC/8A**. X axis, DNA origami, and in-plane dipole orientation are represented by the blue, black and red arrows, respectively. Z axis points towards the image. The in-plane dipole orientation is the projection of half of the double-headed arrow on the plane, and its length is $sin(\boldsymbol{\theta})$. The black curved arrow represents the angle of the dipole relative to the origami ($\boldsymbol{\phi}$). The lengths of the black and blue arrows are 1. The defocused distance in the simulations was 550 nm.

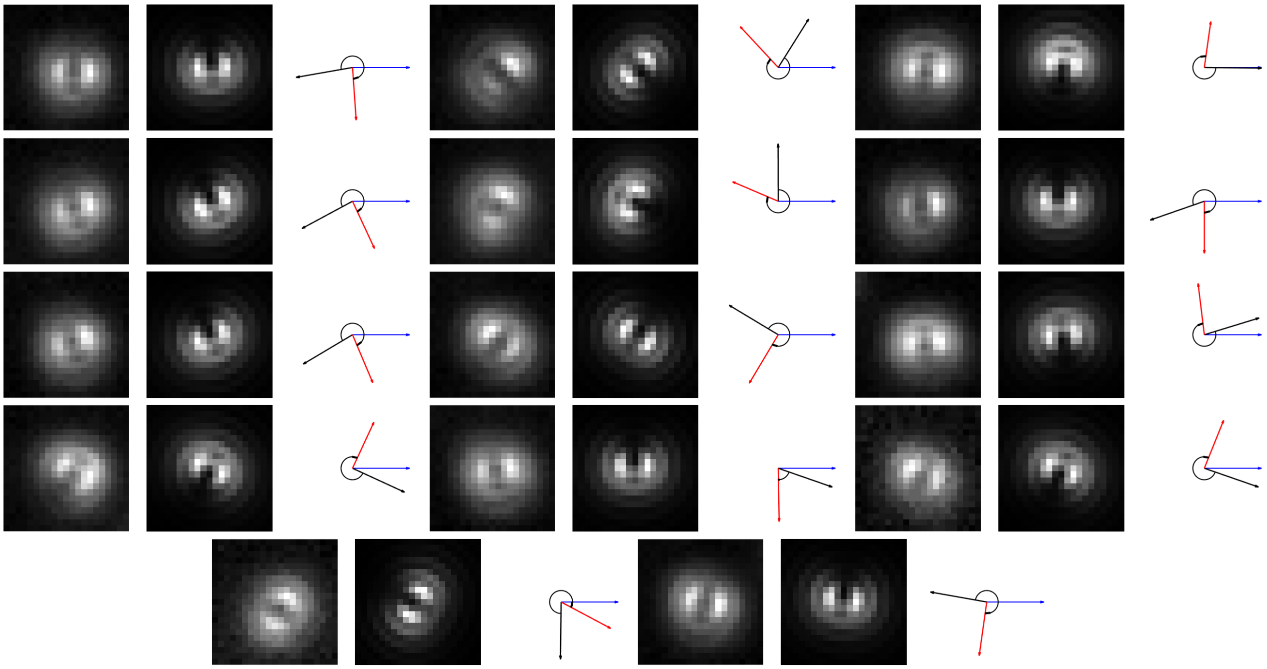

Figure S36: Each set of three sub-figures show the experimental dipole radiation pattern (left), simulated pattern (center) and in-plane orientations (right) in sample **-5GC**. X axis, DNA origami, and in-plane dipole orientation are represented by the blue, black and red arrows, respectively. Z axis points towards the image. The in-plane dipole orientation is the projection of half of the double-headed arrow on the plane, and its length is $sin(\boldsymbol{\theta})$. The black curved arrow represents the angle of the dipole relative to the origami ($\boldsymbol{\phi}$). The lengths of the black and blue arrows are 1. The defocused distance in the simulations was 575 nm.

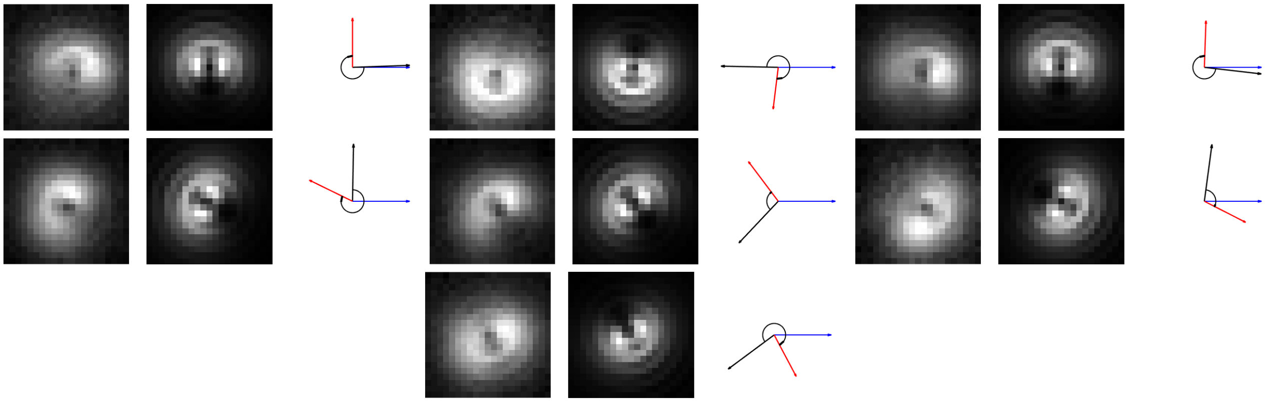

Figure S37: Each set of three sub-figures show the experimental dipole radiation pattern (left), simulated pattern (center) and in-plane orientations (right) in sample **-6GC**. X axis, DNA origami, and in-plane dipole orientation are represented by the blue, black and red arrows, respectively. Z axis points towards the image. The in-plane dipole orientation is the projection of half of the double-headed arrow on the plane, and its length is $sin(\boldsymbol{\theta})$. The black curved arrow represents the angle of the dipole relative to the origami ($\boldsymbol{\phi}$). The lengths of the black and blue arrows are 1. The defocused distance in the simulations was 650 nm.

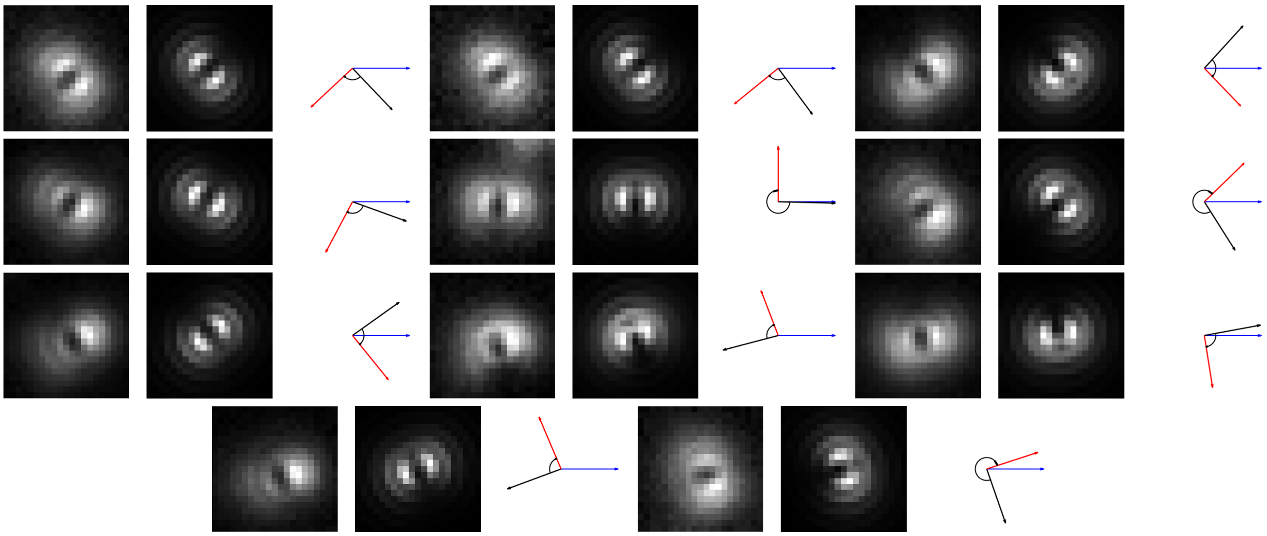

Figure S38: Each set of three sub-figures show the experimental dipole radiation pattern (left), simulated pattern (center) and in-plane orientations (right) in sample **-7GC**. X axis, DNA origami, and in-plane dipole orientation are represented by the blue, black and red arrows, respectively. Z axis points towards the image. The in-plane dipole orientation is the projection of half of the double-headed arrow on the plane, and its length is $sin(\boldsymbol{\theta})$. The black curved arrow represents the angle of the dipole relative to the origami ($\boldsymbol{\phi}$). The lengths of the black and blue arrows are 1. The defocused distance in the simulations was 575 nm.

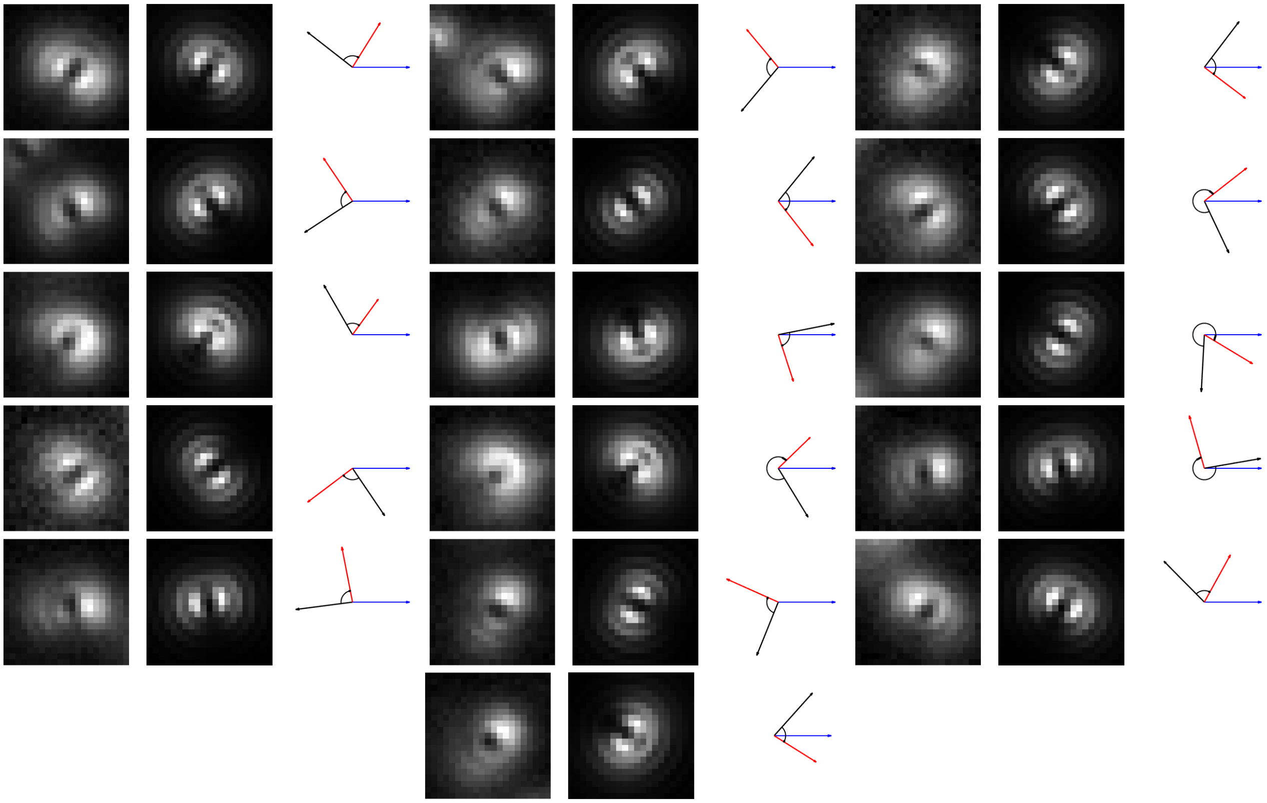

Figure S39: Each set of three sub-figures show the experimental dipole radiation pattern (left), simulated pattern (center) and in-plane orientations (right) in sample **-8GC**. X axis, DNA origami, and in-plane dipole orientation are represented by the blue, black and red arrows, respectively. Z axis points towards the image. The in-plane dipole orientation is the projection of half of the double-headed arrow on the plane, and its length is $sin(\boldsymbol{\theta})$. The black curved arrow represents the angle of the dipole relative to the origami ($\boldsymbol{\phi}$). The lengths of the black and blue arrows are 1. The defocused distance in the simulations was 600 nm.

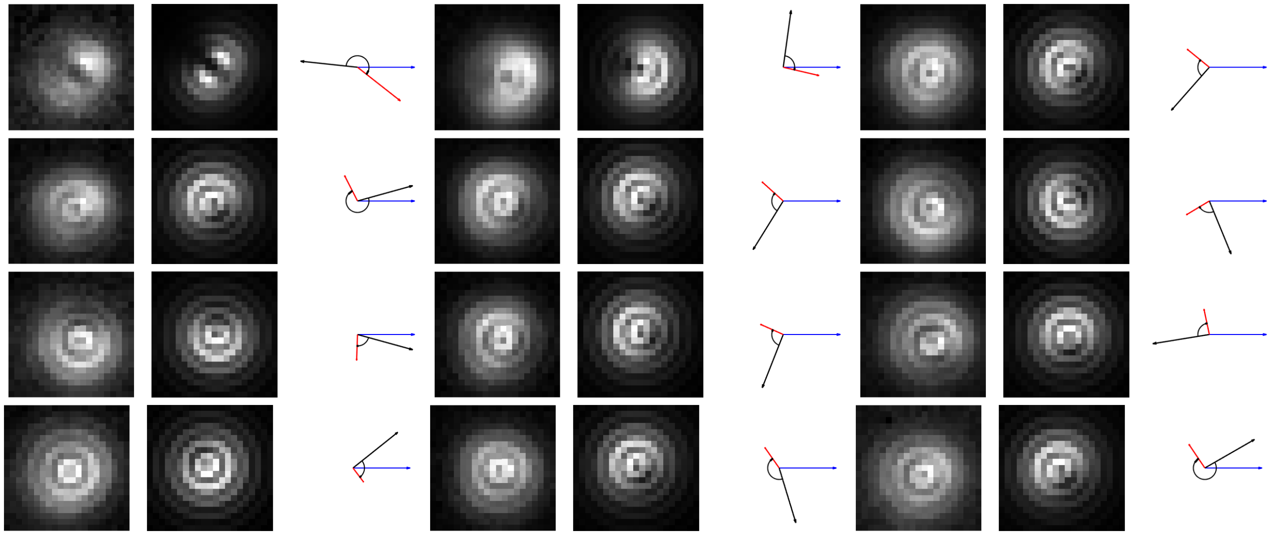

Figure S40: Each set of three sub-figures show the experimental dipole radiation pattern (left), simulated pattern (center) and in-plane orientations (right) in sample **-9GC**. X axis, DNA origami, and in-plane dipole orientation are represented by the blue, black and red arrows, respectively. Z axis points towards the image. The in-plane dipole orientation is the projection of half of the double-headed arrow on the plane, and its length is $sin(\boldsymbol{\theta})$. The black curved arrow represents the angle of the dipole relative to the origami ($\boldsymbol{\phi}$). The lengths of the black and blue arrows are 1. The defocused distance in the simulations was 600 nm.

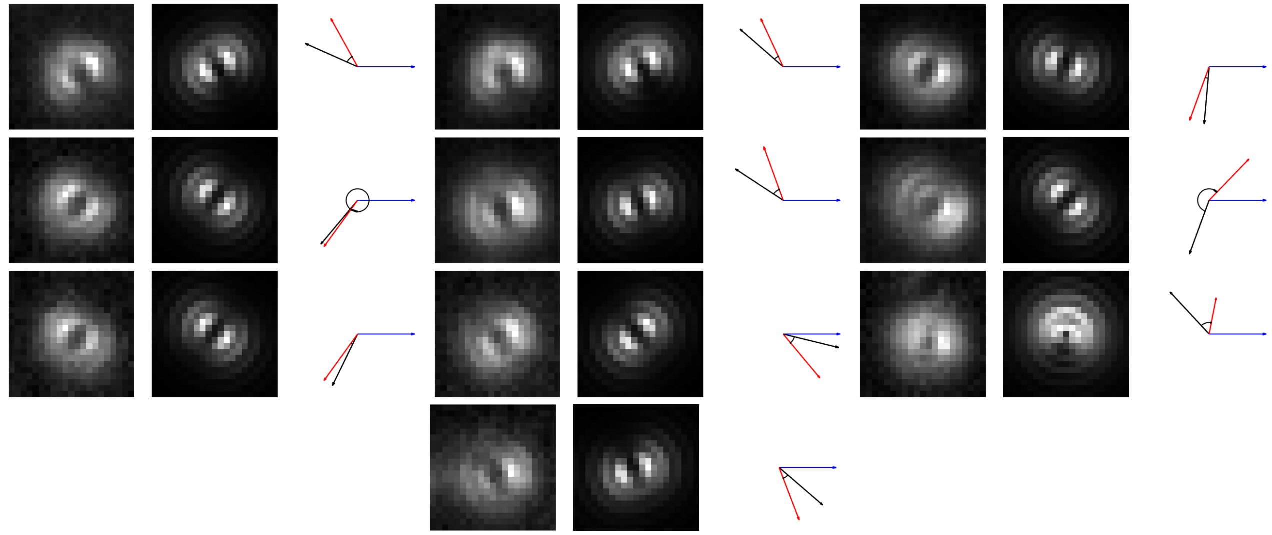

Figure S41: Each set of three sub-figures show the experimental dipole radiation pattern (left), simulated pattern (center) and in-plane orientations (right) in sample **-10GC**. X axis, DNA origami, and in-plane dipole orientation are represented by the blue, black and red arrows, respectively. Z axis points towards the image. The in-plane dipole orientation is the projection of half of the double-headed arrow on the plane, and its length is $sin(\boldsymbol{\theta})$. The black curved arrow represents the angle of the dipole relative to the origami ($\boldsymbol{\phi}$). The lengths of the black and blue arrows are 1. The defocused distance in the simulations was 600 nm.

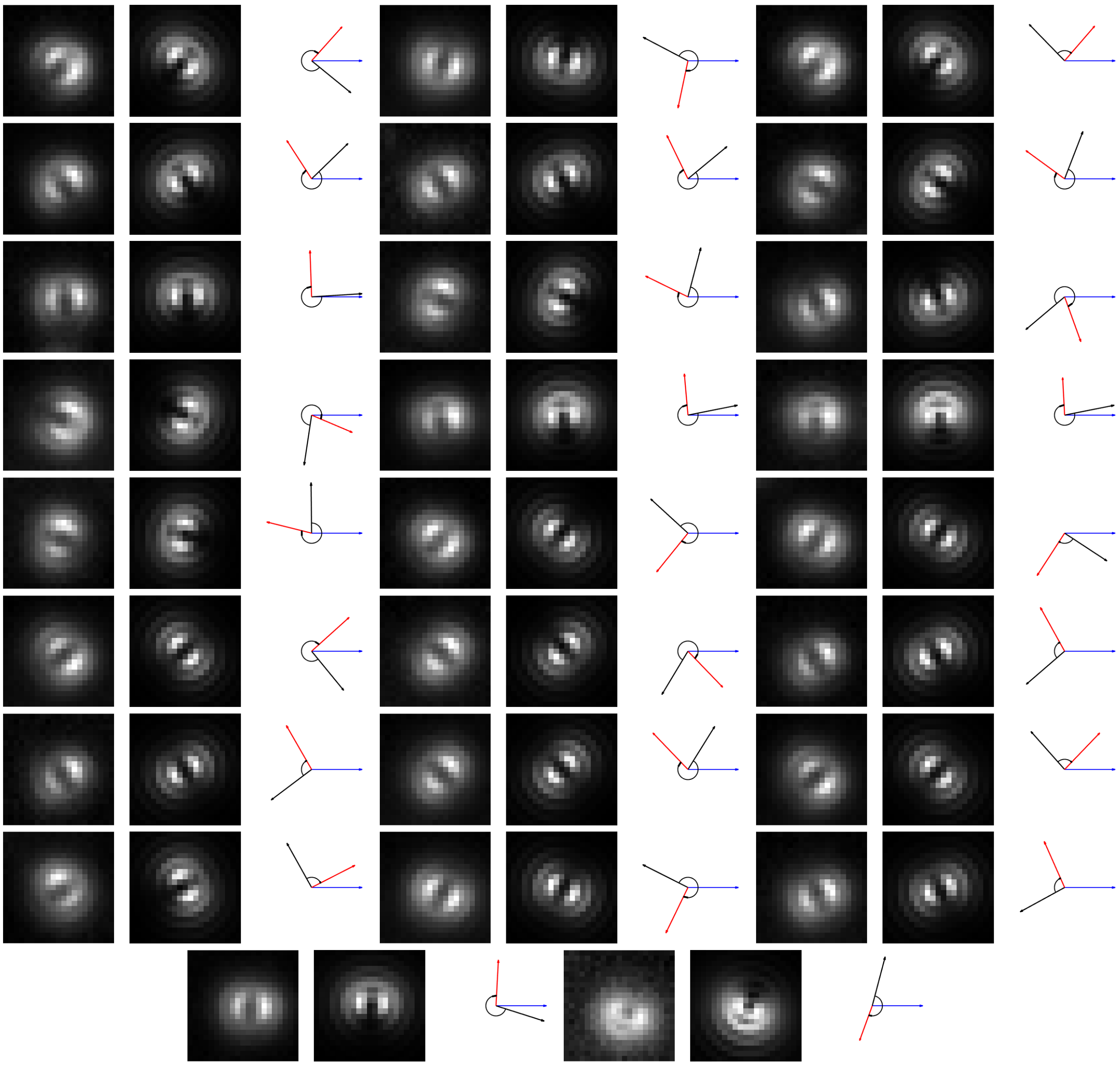

Figure S42: Each set of three sub-figures show the experimental dipole radiation pattern (left), simulated pattern (center) and in-plane orientations (right) in sample **-5TT**. X axis, DNA origami, and in-plane dipole orientation are represented by the blue, black and red arrows, respectively. Z axis points towards the image. The in-plane dipole orientation is the projection of half of the double-headed arrow on the plane, and its length is $sin(\boldsymbol{\theta})$. The black curved arrow represents the angle of the dipole relative to the origami ($\boldsymbol{\phi}$). The lengths of the black and blue arrows are 1. The defocused distance in the simulations was 600 nm.

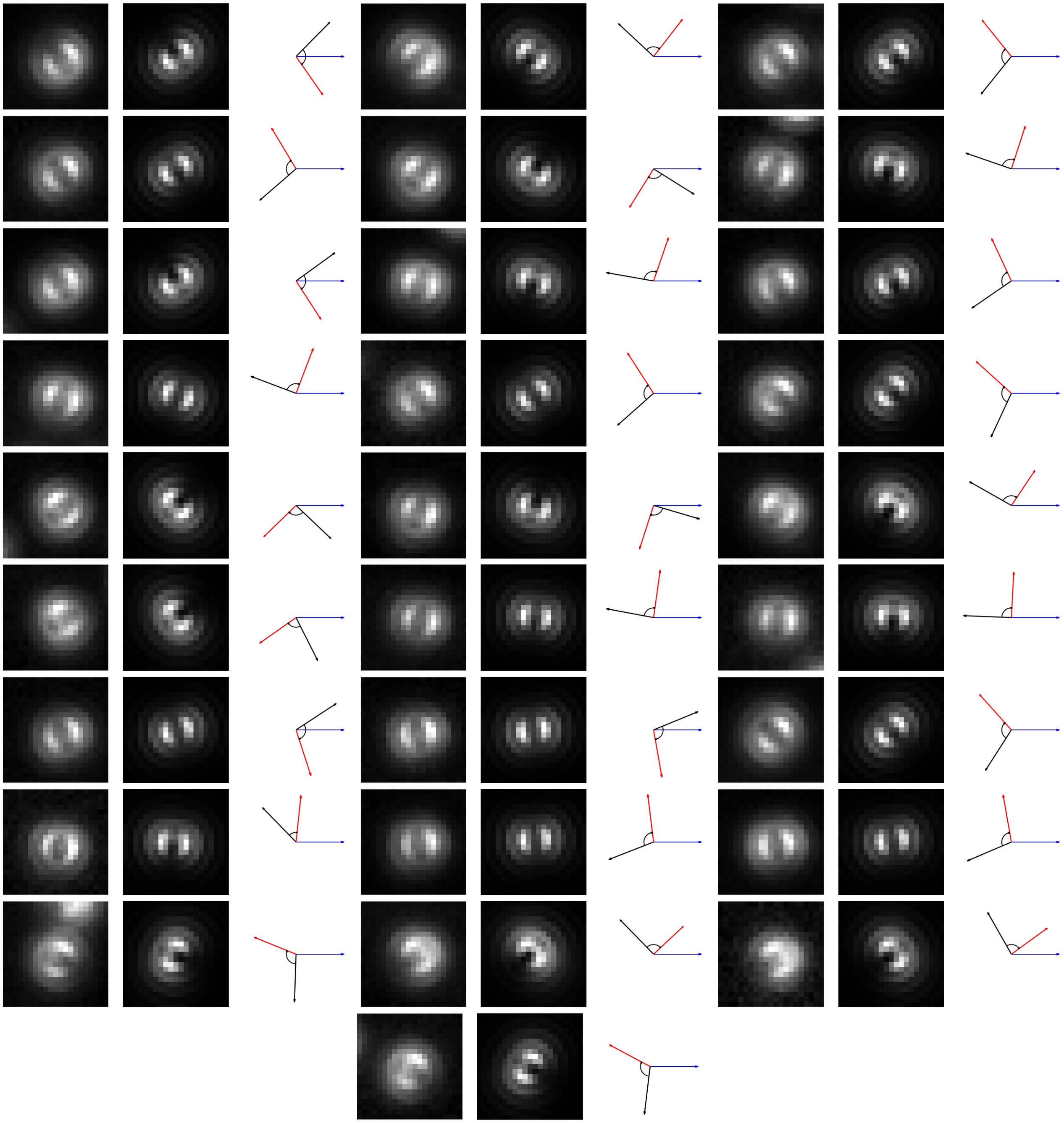

Figure S43: Each set of three sub-figures show the experimental dipole radiation pattern (left), simulated pattern (center) and in-plane orientations (right) in sample **-6TT**. X axis, DNA origami, and in-plane dipole orientation are represented by the blue, black and red arrows, respectively. Z axis points towards the image. The in-plane dipole orientation is the projection of half of the double-headed arrow on the plane, and its length is $sin(\boldsymbol{\theta})$. The black curved arrow represents the angle of the dipole relative to the origami ($\boldsymbol{\phi}$). The lengths of the black and blue arrows are 1. The defocused distance in the simulations was 550 nm.

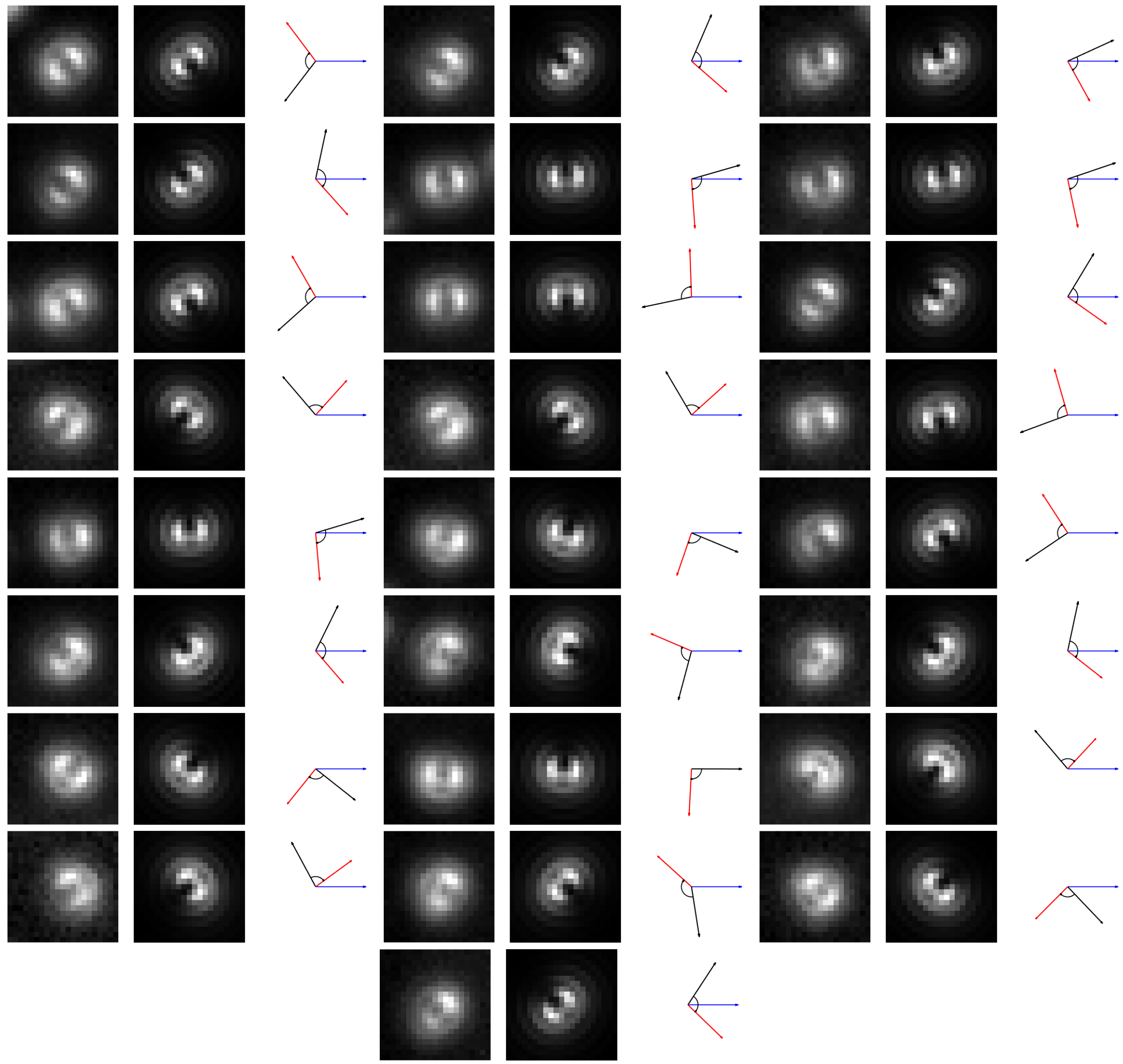

Figure S44: Each set of three sub-figures show the experimental dipole radiation pattern (left), simulated pattern (center) and in-plane orientations (right) in sample **-7TT**. X axis, DNA origami, and in-plane dipole orientation are represented by the blue, black and red arrows, respectively. Z axis points towards the image. The in-plane dipole orientation is the projection of half of the double-headed arrow on the plane, and its length is $sin(\boldsymbol{\theta})$. The black curved arrow represents the angle of the dipole relative to the origami ($\boldsymbol{\phi}$). The lengths of the black and blue arrows are 1. The defocused distance in the simulations was 550 nm.

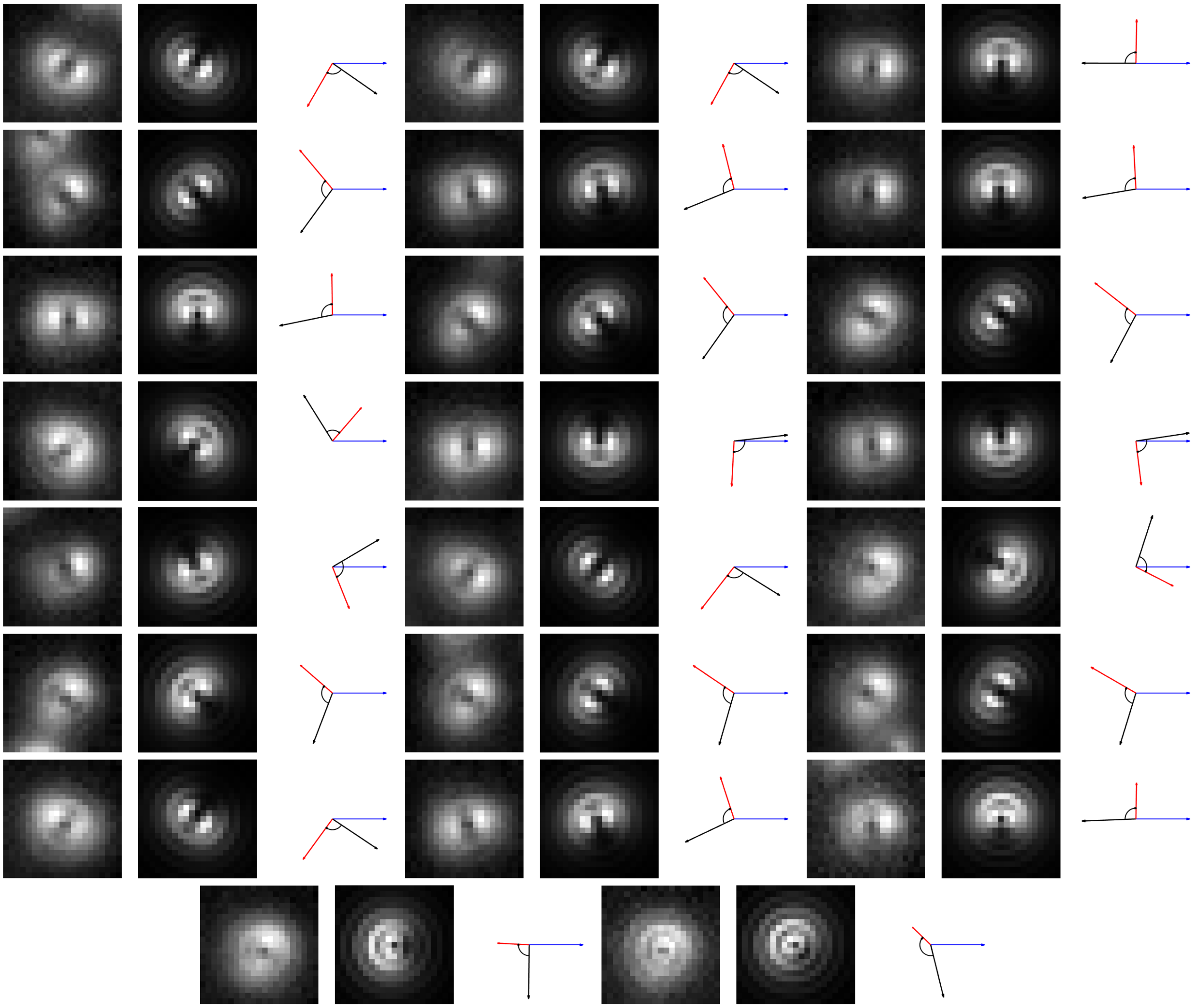

Figure S45: Each set of three sub-figures show the experimental dipole radiation pattern (left), simulated pattern (center) and in-plane orientations (right) in sample **-8TT**. X axis, DNA origami, and in-plane dipole orientation are represented by the blue, black and red arrows, respectively. Z axis points towards the image. The in-plane dipole orientation is the projection of half of the double-headed arrow on the plane, and its length is $sin(\boldsymbol{\theta})$. The black curved arrow represents the angle of the dipole relative to the origami ($\boldsymbol{\phi}$). The lengths of the black and blue arrows are 1. The defocused distance in the simulations was 600 nm.

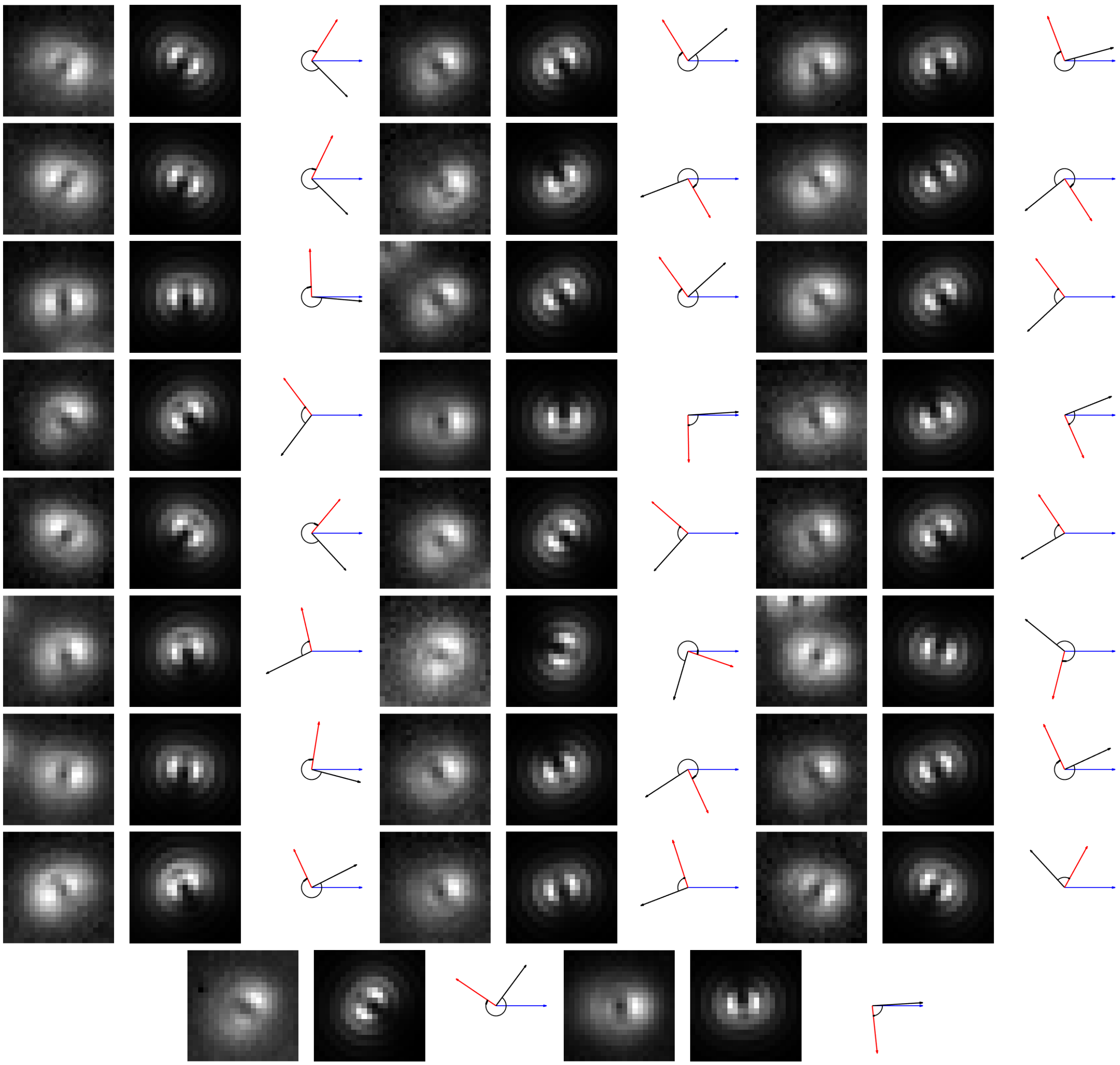

Figure S46: Each set of three sub-figures show the experimental dipole radiation pattern (left), simulated pattern (center) and in-plane orientations (right) in sample **-9TT**. X axis, DNA origami, and in-plane dipole orientation are represented by the blue, black and red arrows, respectively. Z axis points towards the image. The in-plane dipole orientation is the projection of half of the double-headed arrow on the plane, and its length is $sin(\boldsymbol{\theta})$. The black curved arrow represents the angle of the dipole relative to the origami ($\boldsymbol{\phi}$). The lengths of the black and blue arrows are 1. The defocused distance in the simulations was 575 nm.

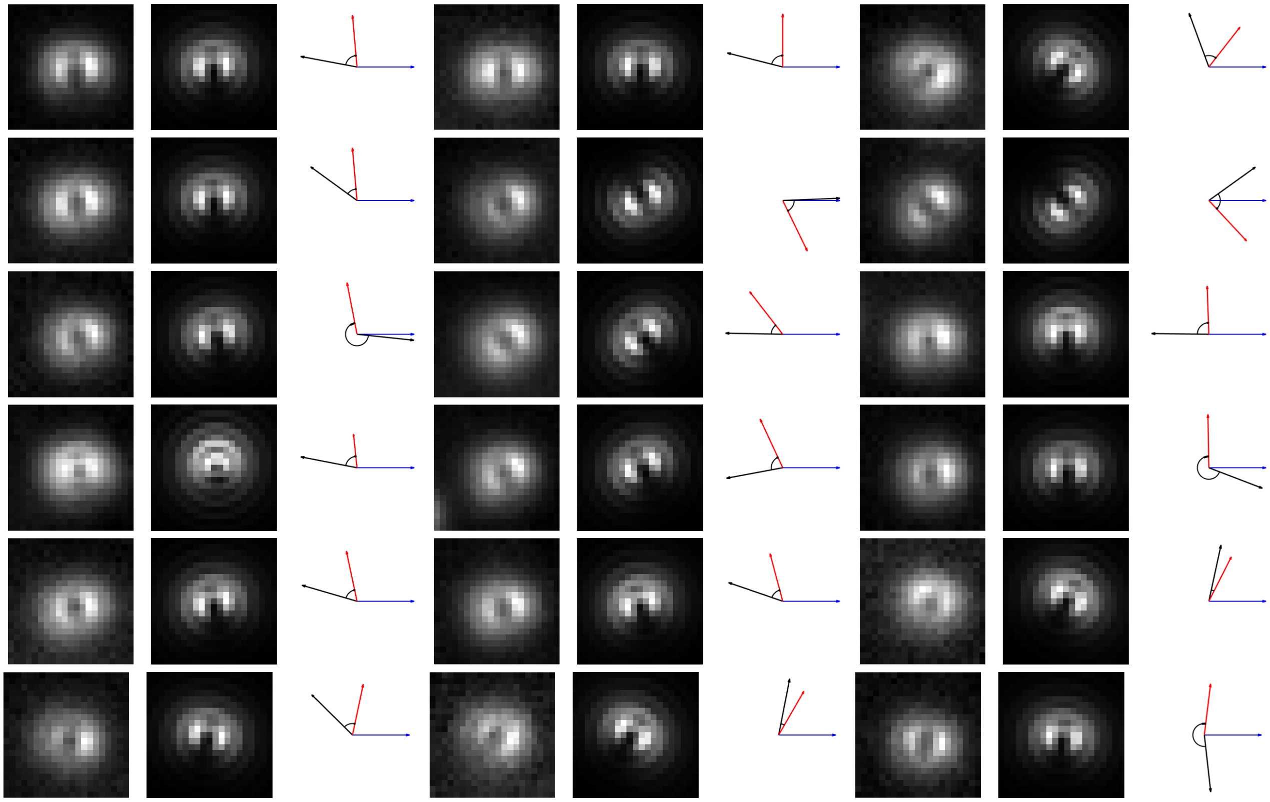

Figure S47: Each set of three sub-figures show the experimental dipole radiation pattern (left), simulated pattern (center) and in-plane orientations (right) in sample **-10TT**. X axis, DNA origami, and in-plane dipole orientation are represented by the blue, black and red arrows, respectively. Z axis points towards the image. The in-plane dipole orientation is the projection of half of the double-headed arrow on the plane, and its length is $sin(\boldsymbol{\theta})$. The black curved arrow represents the angle of the dipole relative to the origami ($\boldsymbol{\phi}$). The lengths of the black and blue arrows are 1. The defocused distance in the simulations was 575 nm.

References

[1] W. K. Olson, A. A. Gorin, X. J. Lu, L. M. Hock, V. B. Zhurkin, *Proceedings of the National Academy of Sciences of the United States of America* **1998**, *95* 11163.

[2] J. T. Kent, *Journal of the Royal Statistical Society: Series B (Methodological)* **1982**, *44* 71.

[3] N. I. Fisher, T. Lewis, B. J. J. Embleton, *Statistical Analysis of Spherical Data*, Cambridge University Press, paperback edition (with errata), **1993**.

[4] P. Leong, S. Carlile, *Journal of Neuroscience Methods* **1998**, *80* 191.

[5] G. D. Dickinson, G. M. Mortuza, W. Clay, L. Piantanida, C. M. Green, C. Watson, E. J. Hayden, T. Andersen, W. Kuang, E. Graugnard, R. Zadegan, W. L. Hughes, *Nature Communications* **2021**, *12* 1.
